# Interactions between tick and transmitted pathogens evolved to minimise competition through nested and coherent networks

**DOI:** 10.1038/srep10361

**Published:** 2015-05-20

**Authors:** Agustín Estrada-Peña, José de la Fuente, Richard S. Ostfeld, Alejandro Cabezas-Cruz

**Affiliations:** 1 Department of Animal Pathology, Faculty of Veterinary Medicine, University of Zaragoza, Spain; 2 SaBio, Instituto de Investigación en Recursos Cinegéticos, IREC (CSIC, UCLM, JCCM), 13005 Ciudad Real, Spain, and Department of Veterinary Pathobiology, Center for Veterinary Health Sciences, Oklahoma State University, Stillwater, OK 74078, USA; 3 Cary Institute of Ecosystem Studies. Millbrook, NY 12545-0129, USA; 4 Center for Infection and Immunity of Lille (CIIL), INSERM U1019 – CNRS UMR 8204, Université Lille Nord de France, Institut Pasteur de Lille, Lille, France

## Abstract

Natural foci of ticks, pathogens, and vertebrate reservoirs display complex relationships that are key to the circulation of pathogens and infection dynamics through the landscape. However, knowledge of the interaction networks involved in transmission of tick-borne pathogens are limited because empirical studies are commonly incomplete or performed at small spatial scales. Here, we applied the methodology of ecological networks to quantify >14,000 interactions among ticks, vertebrates, and pathogens in the western Palearctic. These natural networks are highly structured, modular, coherent, and nested to some degree. We found that the large number of vertebrates in the network contributes to its robustness and persistence. Its structure reduces interspecific competition and allows ample but modular circulation of transmitted pathogens among vertebrates. Accounting for domesticated hosts collapses the network’s modular structure, linking groups of hosts that were previously unconnected and increasing the circulation of pathogens. This framework indicates that ticks and vertebrates interact along the shared environmental gradient, while pathogens are linked to groups of phylogenetically close reservoirs.

Ticks, their hosts, and the pathogens they support constitute a remarkable community of interacting species. Ticks and transmitted pathogens are important in human and animal health nearly worldwide^1^, they are examples of the ecological complexity of parasitic associations in a landscape^2^, and are the paradigm for a continuum ranging from generalist to specialist parasitic associations. Given the ubiquity of host-tick interactions, understanding the factors that generate, maintain, and constrain these associations is of primary interest, with implications for applied ecology and the spread of infectious diseases. Foci of ticks and transmitted pathogens are regulated by both a set of suitable environmental conditions and the availability of vertebrate hosts, which are the blood source for these arthropods and the reservoirs for pathogen circulation^3^. Each triplet of partners (tick, vertebrate, pathogen) interacts and contributes in different ways to maintaining active foci of infection and disease. These interactions result in a finely tuned and spatially variable combination of components of the ecosystem, driving the amplification or diminution of tick-transmitted disease risk^4^,^5^,^6^. Studies of these patterns at various levels of complexity previously explained empirical relationships among specific sets of vertebrate hosts, ticks, and pathogens^7^,^8^. However, unravelling the core mechanisms that underlie these vertebrate-tick-pathogen phenomena might require a more comprehensive understanding of interaction webs and their consequences for the circulation of pathogens that affect human and animal health.

Network approaches have been increasingly used for investigating ecological interactions among species^9^,^10^. These techniques provide helpful frameworks for understanding structural patterns and the functional and complementary roles of species in ecosystems^11^,^12^. Parasitic networks have largely been understudied in these systems, and there have been few attempts to use the topology of the free-living host community to describe parasite dynamics^13^,^14^,^15^. On the other hand, meta-analysis of massive data sets is helpful for assessing ecological, epidemiological, and evolutionary patterns among vertebrate hosts, vectors, and transmitted pathogens, a strategy that can yield important information^16^,^17^,^18^. Species-level data are key when examining large sets of data about tick-pathogen-vertebrate interactions, since it has been established that supraspecific taxonomic levels may lead to incorrect conclusions^19^.

Here, we use data meta-analysis to infer potential inter-species interactions among ticks, transmitted pathogens, and vertebrate reservoirs, construct the network of ecological relationships, and infer conclusions about its robustness. Our goal is to characterise a large community without requiring detailed knowledge of the nature and strengths of the interactions among partners, which is only rarely available at the adequate spatial and temporal scales. We compiled published data spanning the period 1990–2012 on taxonomic associations among ticks, vertebrates, and transmitted pathogens in the western Palearctic, which was defined as countries included within the borders marked by Scandinavia in the north, the Azores in the Atlantic, North African countries in the south, and the Ural Mountains and Turkey in the east. A total of 14,219 records of pairs of ticks and vertebrates, pathogens and ticks, or pathogens and vertebrates were assembled and converted into a network structure for further analysis.

Networks consist of nodes and links that can be used to represent a given system in terms of its components (nodes) and the relations between those components (edges). In the same way that food webs are descriptions of who eats whom in an ecosystem^20^, our application is a description of who is a parasite of whom, and who is a carrier of whom regarding tick-transmitted pathogens, their vectors, and their reservoirs. To represent the network, each node symbolizes a species, and the resulting edge between two nodes represents a relationship, for example “pathogen A detected in tick B,” “pathogen A detected in vertebrate C,” or “tick B recorded on vertebrate C.” The network is therefore directed; each edge links a pathogen “to” a host or a vector. In our application, a “record” is a combination of either pathogen/arthropod or pathogen/reservoir in one site. We computed a set of centrality measures to quantify the properties of the network. Centrality measures are affected by the sampling effort on each species; thus, accurate estimates of centrality must control for variation in sampling. We used the number of citations (number of reports) as an estimate of sampling effort for each species, which were weighted (see Methods) to address the issue of reporting bias^17^. Our purpose is to capture how vertebrates support the persistence of the ticks and pathogens in a network of clusters defined by their species composition in the target region. We produced measures of centrality to understand the specific contribution of each partner to the network coherence. An additional aim was to study the changes in the structure of the network after inclusion of data from domesticated animals. We further used methods of bipartite networks (ticks-vertebrates, pathogens-vertebrates) to evaluate how ticks and pathogens are linked to the vertebrates, explicitly addressing the question of whether the groups of vertebrates sharing the tick and pathogen faunal composition are phylogenetically or environmentally related.

## Results

### Generalist ticks are linked to the same groups of wild vertebrates

**Figure 1:**
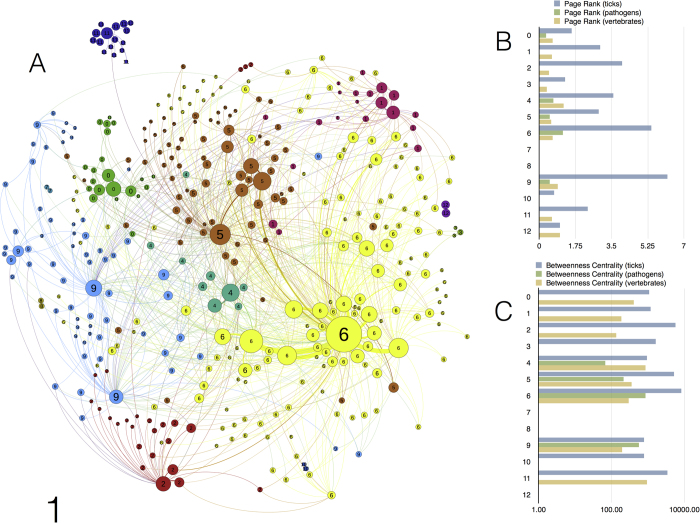
The network of ticks, vertebrates and pathogens without domesticated animals (**a**) The 13 clusters found by the modularity algorithm, represented according to the ForceAtlas2 scheme, randomly coloured, and numbered from 0 to 12 (the agglomerative algorithm include minimum values of 0). Each circle is a partner of the network, a vertebrate, tick, or pathogen (complete taxonomic information is provided in Supplementary Figure 1). The size of each circle is proportional to its NBC. Each line is a link between two nodes, and its colour is the same as that of the cluster. The width of each line is proportional to the weighted degree, a measure of the strength of the link between two nodes. (**b**) Values of the PR index for the ticks, pathogens, and vertebrates of the 13 clusters. (**c**) Values of the NBC for the ticks, pathogens, and vertebrates of the 13 clusters.

The network including only non-domesticated vertebrates has a total of 404 unique nodes at the species level (293 vertebrates, 59 pathogens, and 48 ticks) and 984 edges among nodes. Ticks, non-domesticated vertebrates, and circulating pathogens in the western Palearctic constitute a highly modular network with low density (graph density 0.006; range 0–1). The Louvaine clustering algorithm^21^ identified 13 clusters, which are randomly coloured and numbered according to the ForceAtlas2 algorithm^22^ in Fig. 1 (see Supplementary Fig. 1 for complete taxonomic information). Nestedness in ecological networks is the tendency for specialists to interact with a subset of species that also interact with more generalist species. Clusters 4, 5, and 6 are significantly nested (delineating subnetworks) in comparison to randomised matrices (p < 0.0001) with values of nestedness of 0.75, 076, and 0.81, respectively, calculated using the NODF^23^. These clusters include the more generalist species of ticks that interact with most vertebrates. Clusters 0 and 9 share only some vertebrates with Clusters 4–6, and analyses suggest they are not nested within them (Fig. 1 and Supplementary Fig. 1). Other clusters detected by the modularity algorithm are only marginally connected to the main network or are totally separated. Clusters 1 and 3 contain species of ticks specific to birds that are highly restricted to hosts that are in turn of importance in the context of the network. Cluster 2 is a marginal group of ticks that is slightly associated with some hosts that represent important nodes in the network. The ticks in Cluster 11 are restricted to bats. Clusters 7, 8, 10, and 12 each consist of one species of tick and are related to one species of vertebrate. These last six marginal clusters of ticks do not have pathogens in the network structure. Values of nestedness are always less than 0.2 for clusters other than 4 to 6. Tables 1 and 2 provide taxonomic details of the ticks, pathogens, and vertebrates (at Order and Family levels) included in each cluster of the network. Supplementary Table 1 provides complete specific information of all the hosts for each cluster.

Other details about the significance of the various taxa of pathogens and ticks in the network appear in Table 3. We calculated^24^ the Node Betweenness Centrality (NBC) and the PageRank (PR) of each node as measures of the importance of these taxa in the network. In parasitic networks, host NBC is related to the number of parasites infecting a host^24^ and to the parasite’s host range and transmission ability at the level of the entire network^25^. The PR assigns a universal rank to nodes based on the importance of the other nodes to which it is linked. A node has a high PR if the sum of the ranks of the organisms linked to that node is high. For example, a tick may have a high PR if it transmits prominent pathogens and feeds on vertebrates that are well connected in the network. The PR index thus measures the importance of a node in a network, not only because the node links a high number of species of other organisms, but also due to the relative importance of the organisms linked. As demonstrated elsewhere^24^, NBC and PR provide complementary information. In- and out-degree indices for each genus of pathogens or vectors were calculated. A high out-degree means that a node depends on a relatively higher number of other nodes in the network. A high in-degree means that the node is more supportive of the network.

Our results show that both indices of centrality of ticks are tightly linked to these indices of vertebrates. Genera of ticks with the highest values of degree, PR, and NBC are those with the largest range of vertebrate hosts (generalist ticks) that also have high centrality values. Generalist ticks are the most central to the network because they are linked to the same groups of vertebrates that support the majority of tick species. The tick genus *Ixodes*, which has the highest PR, is associated with vertebrates with the largest values of PR, BNC, and degree and supports the community of pathogens with the highest centrality in the network. The ecological translation is that generalist ticks and associated pathogens exploit species of vertebrates that are most central to the network, allowing the circulation of these pathogens among poorly linked clusters of partners. Pathogens with the highest centrality are not necessarily associated with vertebrates or ticks with similarly high centrality values. Pathogens of the genera *Anaplasma*, *Rickettsia*, and *Babesia* are associated with vertebrates and ticks with high centrality values. However, *Borrelia*, the pathogen with the highest centrality in the complete network, circulates among partners with medium values of these indices. TBEv, which is a virus associated with the tick vectors among which *Borrelia* circulates, has only a marginal importance in the network, reflecting its ecological restriction to a few vertebrate reservoirs. The protozoan *Theileria* has a very low centrality in the network of non-domesticated vertebrates. As a rule, all genera of pathogens with high NBC values circulate as a consequence of the high connectivity of their vectors, independent of their PR.

**Figure 2:**
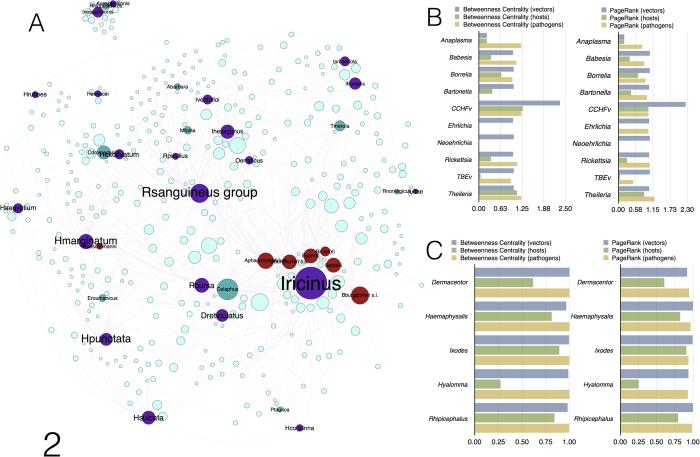
Data about bridge species that support the connections among subnetworks (**a**) Localization of the bridge species in the network structure. Ticks, blue; vertebrates, grey; pathogens, brown. (**b**) Changes in the NBC and PR of pathogens after the removal of vertebrates that act as bridge species. (**c**) Changes in the NBC and PR of ticks after the removal of vertebrates that act as bridge species. In (**b**) and (**c**), histograms reflect the rates of change (1 = no change).

We investigated the existence of “bridge species,” species with special significance to network cohesiveness, using an algorithm originally developed for webs of plants and pollinators^26^. Bridges are nodes that, if removed, result in the collapse of the network into unconnected groups. This analysis revealed the high cohesiveness of the network, because as many as 36 species of partners (Fig. 2) are necessary to disconnect the network. Only nine species of vertebrates act as bridges among clusters. These hosts may be “generalists”, like *Cervus elaphus* (Artiodactyla), *Turdus merula* (Passeriformes) or *Rattus norvegicus* (Rodentia) sharing species of pathogens and ticks that cluster in different groups; other might be specialists hosts, like *Podarcis taurica* (Reptilia), *Alectoris barbara* (Aves), *Camelus dromedarius* (Artiodactyla) or *Rhinolophus hipposideros* (Chiroptera) sharing one species of a tick or pathogen otherwise restricted to another cluster. Twenty species of ticks and seven species of pathogens also bridge clusters. In ecological terms, this finding indicates that clusters are connected by generalist ticks, which function as links among several groups of otherwise unconnected vertebrates. Of particular importance in this context is the bacterial genus *Borrelia*, of which four species bridge clusters. The ecological explanation of this finding is that *Borrelia* is restricted to groups of vertebrates on which its main vector (*Ixodes* ticks) circulates, re-structuring its large cluster into smaller, highly connected subnetworks. The removal of bridge vertebrates produces a drop in the centrality values of pathogens but only slightly affects the values for generalist ticks, which have an ample range of supporting vertebrates (Fig. 2).

### Domesticated hosts collapse the natural structure of the network

**Figure 3:**
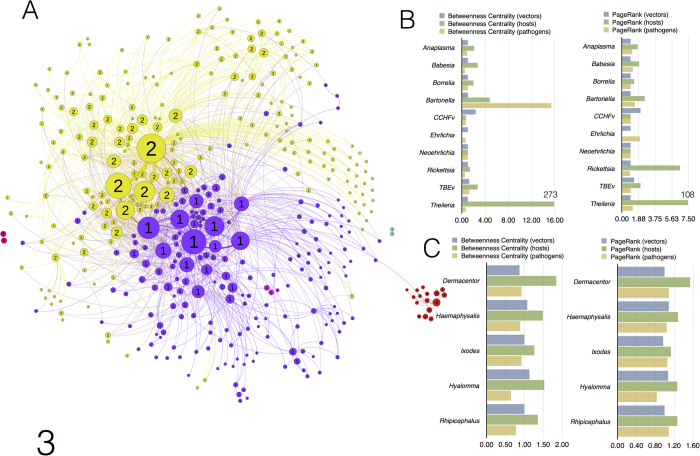
The network of ticks, vertebrates and pathogens with records from domesticated animals (**a**) Changes in the structure modularity of the network of ticks, pathogens, and vertebrates in the western Palearctic after the inclusion of domestic vertebrates. These changes were detected by the modularity algorithm, are represented according to the ForceAtlas2 scheme, and are randomly coloured. Complete taxonomical information appears in Supplementary Figure 2. (**b**) Changes in the NBC and the PR of pathogens after the inclusion of domestic vertebrates in the network. (**c**) Changes in the NBC and the PR of ticks after the inclusion of domestic vertebrates. In (**b**) and (**c**), histograms reflect domestic rates of change (1 = no change). Rates of change of the genus *Theileria* are not drawn to the same scale as the other pathogen genera. Change values are included in the charts.

The addition of domesticated vertebrates to the network produced 420 unique nodes and 1,167 edges. The structure of the network changed dramatically after we recalculated indices of centrality; the modular structure of the network collapsed into only six clusters (Fig. 3). Four of them are formed by monoxenous parasites that are poorly connected with the rest of the network: *Ixodes lividus* on *Riparia riparia*, *Argas reflexus* on *Columba livia*, *Argas persicus* and their hosts, and the ticks on bats, which remain unaffected by the addition of new records. The two larger clusters derive from the fusion of all other partners in the new network. One of the new clusters is formed by ticks of the genera *Dermacentor*, *Haemaphysalis*, *Hyalomma*, and *Rhipicephalus*, together with several species of microorganisms. The second cluster is dominated by all species of *Ixodes*, plus one species each of *Dermacentor*, *Haemaphysalis*, and *Rhipicephalus*. This collapse in the number of clusters results from the inclusion of 16 new hosts that act as super-spreaders of all species of generalist ticks, resulting in a 118% increase in the number of links among network partners and a 1.2–1.8-fold increase in the centrality of vertebrates. This relatively small increase in the number of vertebrates yields dramatic consequences in terms of values of graph density (0.119, 19.8 times higher density). Domesticated hosts also reduce the length of links between nodes of vertebrates and ticks (34% shorter), perhaps serving a measure of the “proximity” of nodes that are satellites of these super-spreaders. In ecological terms, the inclusion of domesticated hosts generates a massive aggregation of the natural network, enabling better circulation of ticks and pathogens and disrupting the natural disconnects among clusters. In the presence of domesticated hosts, ticks and carried pathogens can now contact groups of wild vertebrates that were previously inaccessible.

The centrality indices of pathogens change dramatically when domesticated hosts are included in the network, increasing by 1.9–6.2-fold (excluding *Theileria*). The epidemiological significance of this finding is that domesticated vertebrates acting as bridges among the former clusters of ticks increase the circulation of tick-transmitted pathogens that were previously restricted to a natural set of species. Interestingly, after the inclusion of domesticated hosts, the values for degree, NBC, and PR increased by 23-fold, 253-fold, and 21-fold, respectively, for vertebrates associated with the pathogen *Theileria*. We conclude that *Theileria* does not circulate in networks of ticks and wild vertebrates in the target region, and seems to be restricted only to domesticated ungulates. Its presence in wild ungulates may be a consequence of the co-existence of domesticated vertebrates that connect the pathogen to wild ungulates.

### Generalist ticks share a habitat with their natural hosts, while pathogens infect hosts that are phylogenetically related

It is of interest to address whether species of ticks and pathogens are restricted to vertebrates linked by phylogenetic relationships, or whether they share groups of vertebrates with similar environmental preferences regardless of phylogenetic relationships. The structure of the network was translated to dendrograms of vertebrates and ordered according to their genetic or “environmental” distances. While the concept of genetic distance is straightforward, environmental distance is related to the way in which vertebrates use and share the habitat; the amount of overlap between pairs of vertebrates in the n-dimensional niche of environmental variables is a measurable distance^27^, similar to the genetic distance between two species according to their DNA sequences. Mean pairwise distances and mean nearest taxon distances of the vertebrates in the network were calculated and related to the observed patterns of ticks and pathogens. Standardised effect size compares the relatedness of the pattern to the pattern expected under null models of community randomization. Positive standardised effect sizes and high quantiles indicate molecular or environmental evenness. Negative standardised effect sizes and low quantiles indicate clustering according to molecular or environmental traits.

Cytochrome b nucleotide sequences from 239 species of vertebrates included in the network (Supplementary Table 2) were used to determine molecular relationships. The Newick tree calculated via the Maximum Likelihood method appears in Supplementary Table 3. Environmental distances between vertebrates were calculated with a set of remotely sensed variables^28^, including temperature and the Normalised Difference Vegetation Index from more than 3 million records for 276 species of hosts available from the Global Biodiversity Information Facility (GBIF). The list of hosts for which distribution data were available is included as Supplementary Table 4. Schoener’s D^29^ was used to calculate the portion of shared habitat to generate the tree of environmental distances (Supplementary Table 5).

The species of ticks in Clusters 1 and 6 are environmentally associated with vertebrates (Table 4, Supplementary figures 3 and 4). Cluster 5, which contains a heterogeneous group of taxa, shows a clear relationship with vertebrate hosts based on molecular distances. Cluster 11 includes only parasites of bats, to which they are tied according to phylogenetic relationships. All species of ticks that are restricted to particular phylogenetic groups of hosts are separated from the main flow of the network, appearing as isolated clusters. Interestingly, most species of pathogens are tied to phylogenetically related groups of vertebrate hosts. The relatedness of the species of ticks is more variable, with some species clearly associated with groups of vertebrates that share habitat and others associated with groups of phylogenetically related vertebrates. It is of interest that species of ticks included in Cluster 9 (which is associated with inconsistent results overall) exhibit variable dependence on phylogenetic or environmental links with vertebrates. Cluster 9 thus seems to be a heterogeneous group of ticks that occupy intermediate positions in the network that are not well defined. Additional information about phylogenetic or environmental associations computed separately for each species of ticks and pathogens appears in Supplementary Table 6.

## Discussion

A central goal in ecology is to uncover the basic determinants of trophic interactions among members of natural communities^30^. The present investigation demonstrates that interactions among ticks, vertebrate hosts, and circulating pathogens may be inferred via a data-mining approach using methods for analysing network topology. We applied this strategy to data from the western Palearctic in order to infer key elements of the structure of these communities by visualising interactions among species. Several indices were employed to quantify and summarise presumed relationships among partners, explaining network features and revealing connectivity among partners against an ecological background. Our application is primarily intended to elucidate eco-epidemiological interactions in terms of the circulation of ticks and transmitted pathogens in natural networks. This ecological facet has been commonly neglected when considering the spread and persistence of foci of tick-transmitted pathogens.

Our analyses revealed a structured network with high connectivity among a large number of partners. The network can be further deconstructed into nested clusters, among which some partners act as bridges, providing coherence and improving the circulation of generalist ticks. Nestedness is known to reduce species competition and to enhance the number of coexisting species, which is suitable for a high turnover of tick-transmitted pathogens. Ticks are environmentally associated with vertebrates, and pathogens secondarily segregate according to phylogenetic relationships with their vertebrate hosts. In our network, partners are not organised along a single dimension, but rather are environmentally adapted and then phylogenetically restricted. This general finding cannot be applied to species of ticks that are adapted to a very restricted environment occupied only by highly specialised vertebrates such as bats.

Network cohesiveness is based on a large number of vertebrates, each with high values of centrality, which promote the circulation of ticks and associated pathogens. Clusters of tightly interacting species that drive the modularity of the network yield stable parasite-vertebrate links. The existence of a large number of species linking most clusters supports the hypothesis that these clusters are solid biotic constructs that ensure high circulation of both ticks and pathogens. Clusters of tightly interacting species that drive nestedness and modularity in the network yield stable trophic links, and exploiting these stable links may ensure successful completion of the parasite life cycle. This notion was previously mentioned for food webs and parasite diversity^31^,^32^. An ecological consequence of network cohesiveness is that the associations of ticks, vertebrates, and pathogens may become more robust to perturbation. Notably, population models have demonstrated that if a pathogen enters a particular compartment, the spread of that pathogen may be enhanced within clusters of tightly interacting species^33^. An important consideration is that the majority of ticks and transmitted pathogens fall within densely linked substructures in the network (species with high centrality scores; clusters that are more tightly linked to each other than to species in other parts of the network). Although they can be stable, networks targeting a few hosts (monoxenous ticks) tend to be less resilient than networks with randomly assigned interactions, since they are highly dependent on the occurrence of key hosts. Nested interactions among ticks and vertebrates might drive an optimisation that maximises the persistence of natural communities and reduces competition. Discussions on compartmentalisation in ecological networks began in the 1960s, and the presence of distinct compartments in food webs has been directly correlated with measures of system robustness^33^,^34^,^35^. Most field-derived data reveal that these networks are highly cohesive, with several small groups of species connecting to a single dense core that plays a central role in determining network structure^36^,^37^.

Results from the present investigation support the hypothesis that the intrusion of domesticated hosts into natural foci of ticks and pathogens collapses the network compartments into which ticks and their pathogens are naturally segregated, increasing circulation throughout the network. While the specificity of pathogens for their natural vectors persists, domesticated hosts act as “mixers” of otherwise highly partitioned and consolidated networks of ticks and pathogens. The disruptive effects of domesticated vertebrates are evident; only monoxenous groups of ticks and transmitted pathogens remained isolated following the collapse produced by such intrusion. Domesticated hosts can act as super-spreaders of ticks and the pathogens they carry, linking strata of the network that would be unreachable under natural conditions.

Some species of ticks are strongly tied to specific groups of vertebrates, like the ticks that are bat parasites, *Ixodes lividus* on *Riparia riparia*, and some species of *Argas* that affect only a few species of birds^38^. Each of these groups constitutes separate subnetworks that are, in most cases, unconnected to other clusters and isolated from the eco-epidemiological mainstream. These findings are related to a long-standing question regarding the ecology of parasites: do ticks infest groups of phylogenetically or environmentally related hosts? This question has been addressed in a variety of ways for different groups of parasites and hosts^39^,^40^. Our results reveal that there is not a single answer to this question. More than 70% of ticks and pathogens share hosts that have environmental but not phylogenetic similarities. Two species of ticks that are parasites of birds, *Ixodes frontalis* and *Ixodes arboricola*, are paradigmatic. The first is a parasite of birds frequenting the ground and the second is found on tree hole-nesting birds^41^. However, our results support the hypothesis that the ecological strategies of these two ticks are different from each other. The former is a parasite of phylogenetically related birds that share the same habitat, while the latter is a parasite of birds that only have common environmental o behavioral preferences in using tree holes. Our analyses also indicate that both species of ticks are unrelated in the network of hosts, even though both are parasites of birds. Previous empirical studies in both the field and the laboratory supported other findings described here regarding the widely occurring *Borrelia burgdorferi* complex^42^,^43^. The species of bacteria in this complex are somewhat segregated among diverse hosts that share a phylogenetic background but also exhibit some degree of environmental relatedness. The close phylogenetic relationships of the vertebrates in which *Borrelia burgdorferi* s.l. circulates do not support environmental segregation of bacterial species, which are known to be linked to various groups of vertebrates^42^,^43^. However, these bacteria are transmitted by ticks of the *Ixodes ricinus* complex of ticks, ensuring their circulation across several cluster of otherwise unrelated vertebrates and supporting a high centrality in the network. The bacteria are able to reach groups of phylogenetically unrelated vertebrates because their vector is environmentally linked to a cluster of reservoirs. Overall, the network of pathogens is not supported by vertebrates with prominent positions in the network, but by a multitude of vectors that highly interconnect the pathogens and vertebrates.

Inferring when, where, and to whom parasites are transmitted are key questions in disease ecology. Here, applying the methods of network analysis to a large dataset of associations of ticks, pathogens, and vertebrates revealed rich patterns of adaptability to a large range of vertebrates, mainly because most ticks and vertebrates share large portions of the environmental niche. They therefore “overlap” in the habitat, with the exceptions of a few tick species tied to hosts via strict phylogenetic relationships. The main conclusion is that this overlap ensures high cohesiveness and persistence of the network through the simultaneous availability of several hosts, improving the circulation of pathogens. Our analysis, however, does not consider the effects of vertebrates that do not serve as hosts, and in fact can serve as “sinks” for either ticks or pathogens or both^44^. In contrast, most pathogens are associated with hosts sharing phylogenetic affinities. Our investigation also revealed that domesticated hosts negatively impact these natural networks, increasing the circulation of ticks, making it possible for pathogens to reach newly available biotic niches if a new compatible host-parasite-pathogen association is formed from which parasites can be “spilled back” to native hosts.

The overall approach should apply broadly, but the specific network herein is specific to the western Palearctic. This region displays faunal homogeneity, although some species that are common in neighbouring regions are scarce within the study area. These species are not reliably localised in the structure of the network; they are underrepresented in the data-mining results, resulting in an incomplete set of biotic interactions. The most obvious case is the tick *Ixodes persulcatus*, which occurs over a large range from Ukraine to Japan but is rarely collected west of Ukraine^38^; it is therefore underrepresented in our geographical background, even if it has been collected in western Palearctic. This incidence may impact network structure, but it does not affect the goals of our investigation. Because data mining is based on literature searches, there is uncertainty as to whether the complete set of interactions among hosts, ticks, and pathogens has been sufficiently well recorded, and whether the lack of host-tick associations simply derives from a lack of records or from a true absence of interaction. Records published in the grey literature may be missing, or an unknown number of records may be inaccurate, affecting the network structure determined here. However, the computed indices were weighted following algorithms detailed in the Methods section^17^. Therefore, the number of records of each species, which is commonly a consequence of collection pressure, does not influence the weighted network. These biotic interactions may be incomplete, but the network structure is reliable.

The framework implemented here revealed and unambiguously quantified several basic ecological properties of these systems that were previously unaddressed and that could be easily adapted to similar problems. For example, these methods could be employed to evaluate the coherence of associations according to spatial gradients or genotypic variants of both ticks and pathogens, adding a further level of complexity to the network. Such additional information could reveal hidden associations among strains of pathogens and clusters of ticks or vertebrates, together with the biogeographical background at which they operate.

## Methods

Data on pairs of systematic associations among ticks and vertebrates, pathogens in vertebrates, and pathogens in ticks were compiled from a literature review focused on the western Palearctic. Both Scopus and Web of Science were systematically surveyed using a combination of keywords, as follows. Keywords included the name of the genera of ticks reported from western Palearctic (e.g. *Argas*, *Boophilus*, *Dermacentor*, *Haemaphysalis*, *Hyalomma*, *Ixodes*, *Ornithodoros*, and *Rhipicephalus*) combined with a logical “OR” with the names of the genera of pathogens reported from the same territory (e.g. *Anaplasma*, *Babesia*, *Borrelia*, *Ehrlichia*, *Haemolivia*, *Hepatozoon*, *Neoehrlichia*, *Rickettsia*, *Theileria*, Crimean-Congo hemorrhagic fever virus, Tick-borne encephalitis virus) combined with a logical “OR” with the names of European or northern African countries. We could not establish *a priori* the range of vertebrates to include as keywords in the bibliographical search, nor a reliable method to eliminate publications not dealing with relationships among ticks, pathogens or vertebrates. Therefore we preliminary selected and manually eliminated the publications without mention to these relationships.

A “record” is a combination of pathogens/ticks/vertebrates at one site, that we call herein “partners”. These combinations are always dyadic, involving a pathogen detected in a tick, a pathogen detected in a vertebrate, or a tick collected on a vertebrate. A set of rules was established to remove unreliable or unnecessary information: (1) records lacking a specific determination of pathogen, tick, and/or vertebrate (at the species level) were not included, leading to the exclusion of serological data; (2) every organism reported as detected from ticks while feeding on hosts via molecular analysis was rejected because molecular techniques applied to feeding ticks probably detect nucleic acids of the pathogen in the remnants of the host blood ingested by the ticks: therefore the pathogen cannot be reliably assumed to have been transmitted to the tick (3) data from humans were not included since they are accidental findings; and (4) species of ticks in the group *Ornithodoros erraticus* were included as the complex *sensu lato*, due to the lack of consensus about the species (therefore without reliability about its determination in published reports). Records for *Theileria annae* were not included for the same reasons. The literature review was completed in May 2013. If the same combination of partners was collected in the same site several times (for example through seasonal collections), it was included only once. The same combinations of any partners at different sites were accumulated to produce the weight of the edge linking that couple of partners.

Networks represent system components (nodes) and the relations between those components (links). Each node represents a species, and the resulting link between two nodes represents a relationship. The network thus is directed; each edge links a pathogen “to” a vertebrate or a vector. Host-parasite data are sensitive to sampling effort. Consequently, the computation of individual centralities is largely influenced by the intensity of sampling and reporting. To ensure that our findings are robust, we used an approach employed in similar studies^17^ of controlling for variation in sampling effort, including sampling effort in the computation of centrality estimates by up-weighting the least sampled species and down-weighting the most sampled species. Specifically, we regressed the weight of each edge against the number of citations of the least sampled species (vertebrate, tick, pathogen) in each edge. This regression was highly significant (standardized beta = 0.49 ± 0.002, t = 35.25, P < 0.0001, R^2^ = 0.34; linear regression). Afterwards, we additively rescaled the residuals to be greater than zero. The residuals would reflect the number of links relative to sampling effort, under the assumption that the measure of sampling effort should be from the lesser studied species. We replaced the original weights of the edges (number of parasites shared per pair of vertebrate species) by the rescaled residuals, and then computed all the centrality estimates.

Several indices were used to measure network properties. We measured nestedness using the NODF^23^. The weighted degree is a simple measure of the number of edges leaving (or arriving at) a given node. It provides an estimation of how many nodes are connected to every single node, but does not evaluate the importance of such nodes in the context of the network. Centrality measures of ecological networks imply that there are some high-ranking nodes in the network that have significantly higher than average connectivity and/or have links that stretch far beyond their local network neighbourhoods^45^. The Node Betweenness Centrality (NBC) indicates how often a node is found on the shortest path between two nodes in the network^45^ incorporating the cost of flow pathways as a consequence of the weights of the edges. The implicit meaning of NBC in our application is the importance of a node in the flow of other network components (how likely that node is to be the most direct route between two other nodes in the network). The PageRank (PR) is another index of centrality that assigns a universal rank to nodes based on the importance of the other nodes to which it is linked^45^. Therefore, the NBC and PR are complementary measures for capturing the importance of each node in the linkage of other nodes throughout the network.

Real-world networks have been shown to separate into logical clusters in which nodes are tightly connected to each other but only loosely connected to nodes outside of their module^46^. This modularity separates the complete network into compartments that can be observed as naturally segregated niches in which a subset of species of vertebrates, ticks, and pathogens have a statistically higher affinity among them than with other species in the network. We calculated modularity twice: with the dataset including only wild hosts and with the dataset including domesticated hosts, using the Louvaine clustering algorithm^21^. We wanted to specifically evaluate whether domesticated hosts have a measurable effect on the centrality indices describing the network. All computations were carried out with the network analysis package igraph [Csardi, G., & Nepusz, T. The igraph software package for complex network research, *InterJournal Complex Systems* **1695** (2006) http://igraph.org, accessed January 2015] for the R development framework [Core Team. R: A language and environment for statistical computing. R Foundation for Statistical Computing, Vienna, Austria. URL http://www.R-project.org/, accessed February, 2015]. The network was visualised using the ForceAtlas2 algorithm^22^. We evaluated the existence of bridge species using the package bipartite [Dormann, C.F. How to be a specialist? Quantifying specialisation in pollination networks. *Network Biol.* **1**, 1–20 (2011)] for R and excluding records about domesticated animals because the results in the calculation of modularity already showed a collapse of the natural network.

We examined the biological diversity of ticks and pathogens in an explicitly evolutionary context to address whether ticks and transmitted pathogens are associated with hosts due to the phylogenetic relationships among them or because they share similar environmental conditions and therefore overlap in suitable spaces. We quantified the phylogenetic and environmental signals of the vertebrates to determine how trait variation is related to these signals. When signal is high, closely related species exhibit similar traits; this similarity decreases as the evolutionary distance between species increases. We calculated a distance matrix based on phylogenetic affinities and another based on environmental relatedness. Cytochrome b nucleotide sequences from 239 vertebrates in the network were collected from GenBank (Supplementary Table 2). Not all vertebrate species in the network were available in GenBank. The sequences were aligned with MAFFT (v7) configured to maximise accuracy^47^. After alignment, regions with gaps were removed and the phylogenetic tree was reconstructed using maximum likelihood methods^48^,^49^. The reliability of the internal branches of this tree was assessed using the approximate likelihood ratio test^49^ (SH-like).

The environmental distances between vertebrates were produced by calculating the distance in the shared niche as delimited by a set of environmental variables^27^ using correlative modelling on a set of occurrences for each species. First, we obtained a list of unique records with reliable coordinates for each species of vertebrates in the network through the GBIF (Supplementary Table 3), using the package dismo [C.F. How to be a specialist? Quantifying specialisation in pollination networks. *Network Biol.* **1**, 1–20 (2011), accessed December, 2014] for R. We obtained ~3,500,000 records with coordinates for 276 hosts. Record collection was completed in June 2014. Then, we selected 10 environmental variables, the coefficients of a harmonic regression performed on the monthly information on land-surface temperature and the Normalised Difference Vegetation Index as recorded at a spatial resolution of 0.05° by the MODIS series of satellites for the period 2000–2012. Details of the calculation of the original series of data, the rationale behind the use of the coefficients of a harmonic regression, and the R script used to calculate the coefficients were provided elsewhere^27^. The superior performance of harmonic regression over other environmental data for correlative modelling has also been discussed previously^28^. The purpose of correlative modelling in our application is to compute the distances between species of vertebrates in the environmental volume, which is a measure of the “environmental relatedness” of the vertebrates.

We computed correlative modelling using the maximum entropy approach for correlative modelling. We used the lineal and quadratic features, with a maximum number of 10,000 background points, 10 replicates per species modeled, and 70% of points for training purposes, using crossvalidate for comparing the resulting models. The regularization multiplier was set to 1. The distance between pairs of species of vertebrates was calculated as the response of the species along the 10 environmental variables, using Schoener’s D index^29^ on the raw output from MaxEnt. We obtained matrices of distances for molecular or environmental relatedness. We calculated mean pairwise distances and mean nearest taxon distances from each distance matrix (phylogenetic distances between hosts using cytochrome b sequences or distances between the environmental niches of the hosts). Null models that randomised the tips of the trees were used to compare relatedness among vertebrates and traits in the community of ticks or pathogens. Standardised effect sizes of the phylogenetic community structure were calculated for mean pairwise distances and mean nearest taxon distances by comparing the observed phylogenetic relatedness to the pattern expected under a null model of phylogeny or community randomisation. Standardised effect sizes describe the difference between phylogenetic distances in the observed communities versus null communities generated via randomisation, divided by the standard deviation of the phylogenetic distances in the null data.

## Author Contributions

A.E.P. designed the work and prepared the figures. A.E.P. and A.C.C. obtained the results. All authors wrote the manuscript and approved the final version.

## Additional Information

**How to cite this article**: Estrada-Peña, A. *et al.* Interactions between tick and transmitted pathogens evolved to minimise competition through nested and coherent networks. *Sci. Rep.* **5**, 10361; doi: 10.1038/srep10361 (2015).

## Supplementary Material

Supplementary Information

## Figures and Tables

**Table 1:** **General details of the taxonomic composition of ticks and pathogens of the network in** Figure 1 **(without domestic vertebrates).** The NBC and the PR of ticks, vertebrates, and pathogens are presented. Complete information on host families, genera, and species is provided in supplementary table 1.

**Cluster**	**Species of ticks**	**Species of pathogens**	**NBC (ticks)**	**NBC (pathogens)**	**NBC (hosts)**	**PR (ticks)**	**PR (pathogens)**	**PR (hosts)**
**0**	*Hy. anatolicum, Hy. dromedarii, Hy. excavatum, Hy. impeltatum, H. erinacei, O. tholozani*	*R. mongolotimomae*	1066.18	0	410.78	1.58	0.33	0.64
**1**	*I. arboricola, I. frontalis*	None	1160.97	NA	186.19	2.94	NA	0.63
**2**	*H. sulcata, I. laguri*	None	5674.99	NA	135.34	4.02	NA	0.46
**3**	*I. uriae*	None	1639.86	NA	0	1.26	0	0.36
**4**	*D. marginatus, Hy. lusitanicum*	*R. raoultii, R. slovaca, C. burnettii*	953.56	65.83	867.8	3.58	0.70	1.17
**5**	*I. canisuga, I. crenulatus, I. hexagonus, I. rugicollis, I. ventalloi, O. erraticus, R. pusillus, R. sanguineus group*	*A. bovis, A. ovis, A. platys, B. hispanica, E. canis, H. canis, R. conorii, R. massiliae, R. sibirica,*	5174.59	213.63	353.16	2.87	0.52	0.58
**6**	*D. reticulatus, H. concinna, I. acuminatus, I. apronophorus, I. eldaricus, I. persulcatus, I. redikorzevi, I. ricinus, I. trinaguliceps, R. bursa, R. rossicus*	*A. marginale, A. phagocytophilum, B. bovis, B. canis, B. capreoli, B. divergens, B. EU1, Ba. birtelsii, Ba. clarridgeiae, Ba. grahamii, Ba. henselae, Ba. schoenbuchensis, Ba. vinsonii, Bo. afzelii, Bo. bavariensis, Bo. burgdorferi, Bo. garinii, Bo. lusitaniae, Bo. miyamotoi, Bo. spielmanii, Bo. turdi, Bo. valaisiana, E. walkerii, N. mikurensis, R. heilongjiangensis, R. helvetica, R. monacensis, TBEv, T. annulata, T. ovis.*	8303.79	854.40	293.73	5.41	1.14	0.65
**7**	*A. persicus*	None	NA	NA	NA	NA	NA	NA
**8**	*A. reflexus*	None	NA	NA	NA	NA	NA	NA
**9**	*H. inermis, H. punctata, Hy. aegyptium, Hy. marginatum, Hy. rufipes*	*B. bigemina, B. caballi, CCHFv, He. mauritanica, R. africae, T. buffeli, T. equi*	761.70	571.3	198.3	6.21	0.50	0.91
**10**	*H. parva*	*R. hoogstraali*	786.00	0	0.94	0.72	0	0.51
**11**	*A. vespertilionis, I. simplex, I. vespertilionis*	None	3359.33	0	938.62	2.36	0	0.63
**12**	*I. lividus*	None	0	0	0	1	0	1

**Table 2:** **Complete information on the characteristics of the network for genera of pathogens.** For each genus, data are provided on the number of species recorded and the species of ticks and hosts for which data have been recorded. Information on Degree, NBC, and PR are also included.

**Genus of pathogen**	**# Species (pathogens)**	**# Species (hosts)**	**In-Degree (hosts)**	**NBC (hosts)**	**PR (hosts)**	**# Species (vectors)**	**In - Out-Degree (vectors)**	**NBC (vectors)**	**PR (vectors)**	**Out-Degree (pathogens)**	**NBC (pathogens)**	**PR (pathogens)**
*Anaplasma*	5	17	193	26893	31	15	631 - 165	182918	115	38	7093	6
*Babesia*	9	11	135	19106	21	10	136 - 315	147181	84	54	6184	9
*Borrelia*	11	41	249	27820	44	6	58 - 183	88319	47	98	16296	18
*Bartonella*	6	3	28	3151	5	2	60 - 162	85695	42	8	0	2
*CCHFv*	1	1	11	1821	2	4	83 - 289	61376	33	5	631	1
*Ehrlichia*	3	0	NA	NA	NA	6	107 - 295	142096	75	7	870	1
*Neoehrlichia*	1	4	67	6081	10	1	42 - 151	82576	38	5	2	1
*Rickettsia*	13	7	107	17596	17	16	158 - 436	176134	110	40	3744	9
*TBEv*	1	7	119	18514	18	2	52 - 153	82760	40	11	390	2
*Theileria*	4	1	7	159	1	6	106 - 284	118642	71	19	784	3

**Table 3:** **Complete information on the characteristics of the network for genera of ticks.** For each genus, data are provided on the number of pathogens that have been recorded and the number of species of vertebrates for which data have been recorded. Information on Degree, NBC, and PR are also included.

**Genus of tick**	**# of species (vectors)**	**# of species (hosts)**	**In-Degree (hosts)**	**NBC (hosts)**	**PR (hosts)**	**# of species (pathogens)**	**In - Out-Degree (vectors)**	**NBC (vectors)**	**PR (vectors)**	**Out-Degree (pathogens)**	**BNC (pathogens)**	**PR (pathogens)**
*Argas*	3	5	8	11418	4	0	0 - 5	1166	3	NA	NA	NA
*Dermacentor*	2	28	262	36542	43	22	29 - 36	4795	10	218	31860	36
*Haemaphysalis*	5	79	365	51982	69	17	20 - 97	28073	23	152	25959	27
*Ixodes*	21	184	622	81478	130	40	59 - 296	105234	73	283	37702	49
*Hyalomma*	8	93	353	48113	71	13	25 - 123	30409	29	130	20893	22
*Rhipicephalus*	5	71	343	61133	64	24	43 - 91	44860	24	166	24534	28

**Table 4:** **Relatedness of the clusters of ticks and pathogens (without domestic vertebrates) to vertebrate dendrograms calculated according to molecular distances or to environmental distances.** The number of species of vertebrates in each cluster available for calculation is indicated (ntaxa). Positive values in the column mpd.obs.z and high quantiles (mpd.obs.p > 0.95) indicate phylogenetic evenness. Negative values in the column mpd.obs.z and low quantiles (mpd.obs.p < 0.05) indicate clustering to molecular or environmental features shared with the hosts. NA, not available (fewer than two species available).

	**Molecular distances**			**Environmental distances**		
	**ntaxa**	**mpd.obs.z**	**mpd.obs.p**	**ntaxa**	**mpd.obs.z**	**mpd.obs.p**
Cluster 0	15	-0.53	0.32	16	1.80	0.98
Cluster 1	33	-1.24	0.09	37	-4.28	0.01
Cluster 2	26	0.30	0.73	25	0.05	0.55
Cluster 3	3	-0.77	0.17	3	-1.53	0.08
Cluster 4	21	-1.96	0.02	22	-1.20	0.15
Cluster 5	54	-3.39	0.01	62	3.45	1.00
Cluster 6	133	1.46	0.93	145	-7.16	0.01
Cluster 9	85	-4.75	0.01	79	-3.53	0.01
Cluster 10	1	NA	NA	0	NA	NA
Cluster 11	11	-2.03	0.01	0	NA	NA
Cluster 12	0	NA	NA	1	NA	NA
