# Supplementary material for: Interactions between tick and transmitted pathogens evolved to minimise competition through nested and coherent networks
Source: Sci Rep. 2015 May 20;5:10361. doi: 10.1038/srep10361 (PMC4438610; doi:10.1038/srep10361)
Supplement: Supplementary Information [file srep10361-s1.pdf]

Supplementary Material

Interactions between ticks and transmitted pathogens evolved to minimise competition through nested and coherent networks.

Agustín Estrada-Peña, José de la Fuente, Richard S. Ostfeld, Alejandro Cabezas-Cruz

Supplementary Figure 1: Complete taxonomic information on the species of ticks, vertebrates, and hosts forming the network of partners in the western Palearctic, excluding domesticated vertebrates. The file is zoomable without loss of resolution, in order to render legible the complete names of organisms. The size of each circle is proportional to its NBC, and the size of the label is proportional to its PR. Clusters are coloured randomly, and each link between partners (lines) has the same colour as the cluster. The width of each link is proportional to its weighted degree, a measure of the strength of the relationship between two partners.

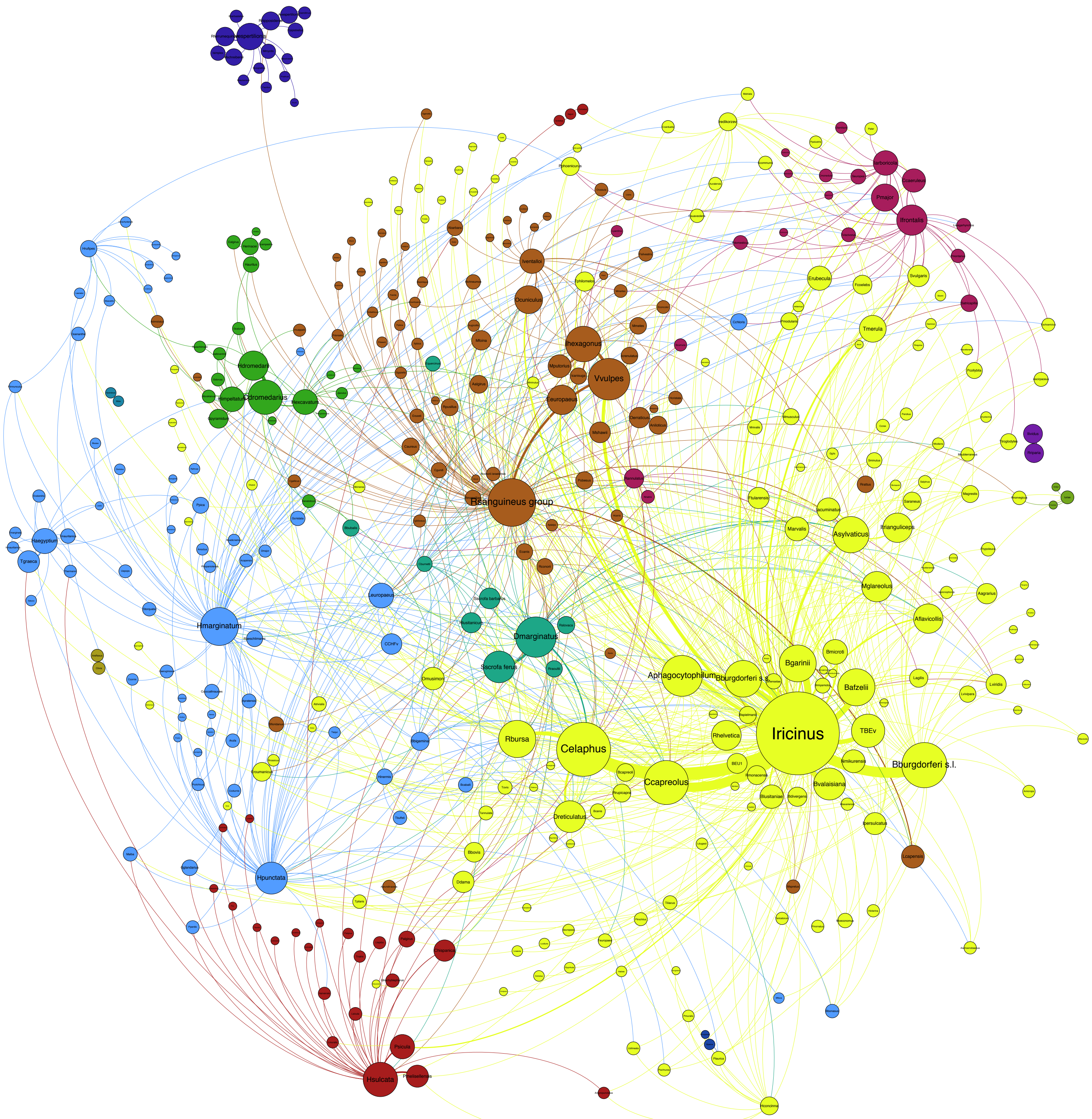

Iricinus
Rsanguineus group
Celaphus
Ccapreolus
Hmarginatum
Hpunctata
Hsulcata
Dmarginatus
Sscrofa ferus
Bburgdorferi s.l.
Bburgdorferi s.s.
Aphagocytophilum
Bgarinii
Bafzelii
TBEv
Bvalaisiana
Rbursa
Vvulpes
Ehexagonus
Ocuniculus
Eeuropaeus
Ifrontalis
Pmajor
Cdromedarius
Hdromedarii
Haegyptium
Tgraeca
Asylvaticus
Mglareolus
Aflavicollis
Rhelvetica
Dreticulatus
Ddama
Pocula
Eeuropaeus
CCHFv
Ivertalloi
Mshawi
Tmerula
Erubecula
Marvalis
Lopenalsi

Supplementary Figure 2: Complete taxonomic information on the species of ticks, vertebrates, and hosts forming the network of partners in the western Palearctic, including domesticated vertebrates. The file is zoomable. Sizes, colours, and links are as in Supplementary Figure 1.

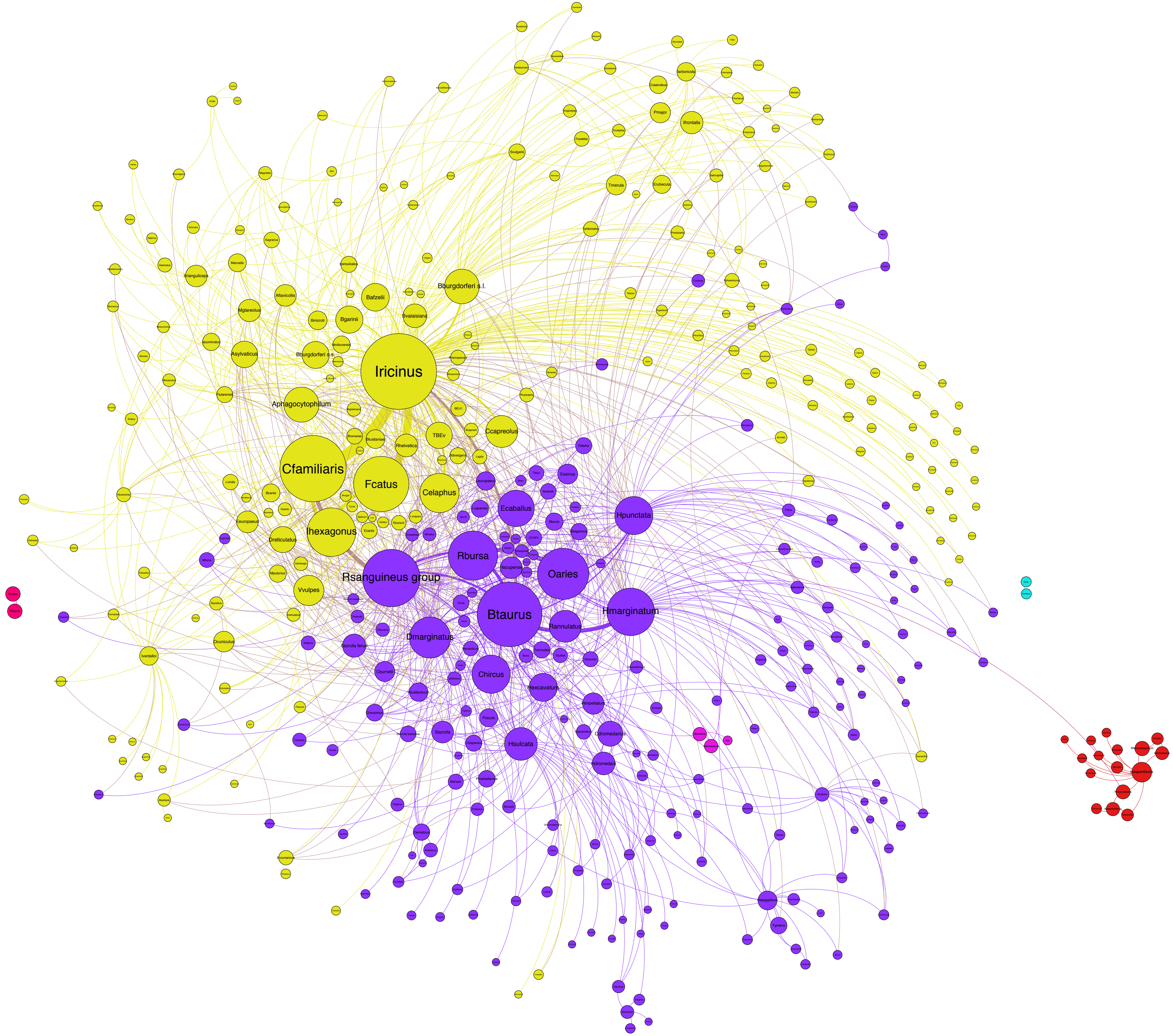

Iricinus
Cfamiliaris
Fcatus
Celaphus
Ihexagonus
Aphagocytophilum
Ccapreolus
Bburgdorferi s.l.
Bafzelii
Bgarinii
Bvalaisiana
TBEv
Rhelvetica
Dreticulatus
Vvulpes
Asylvaticus
Aflavicollis
Ocuniculus
Rsanguineus group
Rbursa
Btaurus
Oaries
Hmarginatum
Hpunctata
Ecaballus
Dmarginatus
Chircus
Hexcavatum
Hsulcata
Rannulatus
Cdromedarius
Pmajor
Ifrontalis
Tmerula

Supplementary Figure 3: Dendrograms of the cytochrome b sequences available in GenBank for vertebrate species in the network of ticks, pathogens, and vertebrates in the western Palearctic, overlaid with the species of ticks and pathogens recorded for each species (blue dots). These dendrograms complement the information in Table 2, and are produced separately for each cluster of species detected in the main network. The figures are zoomable without loss of resolution.

Cluster 0

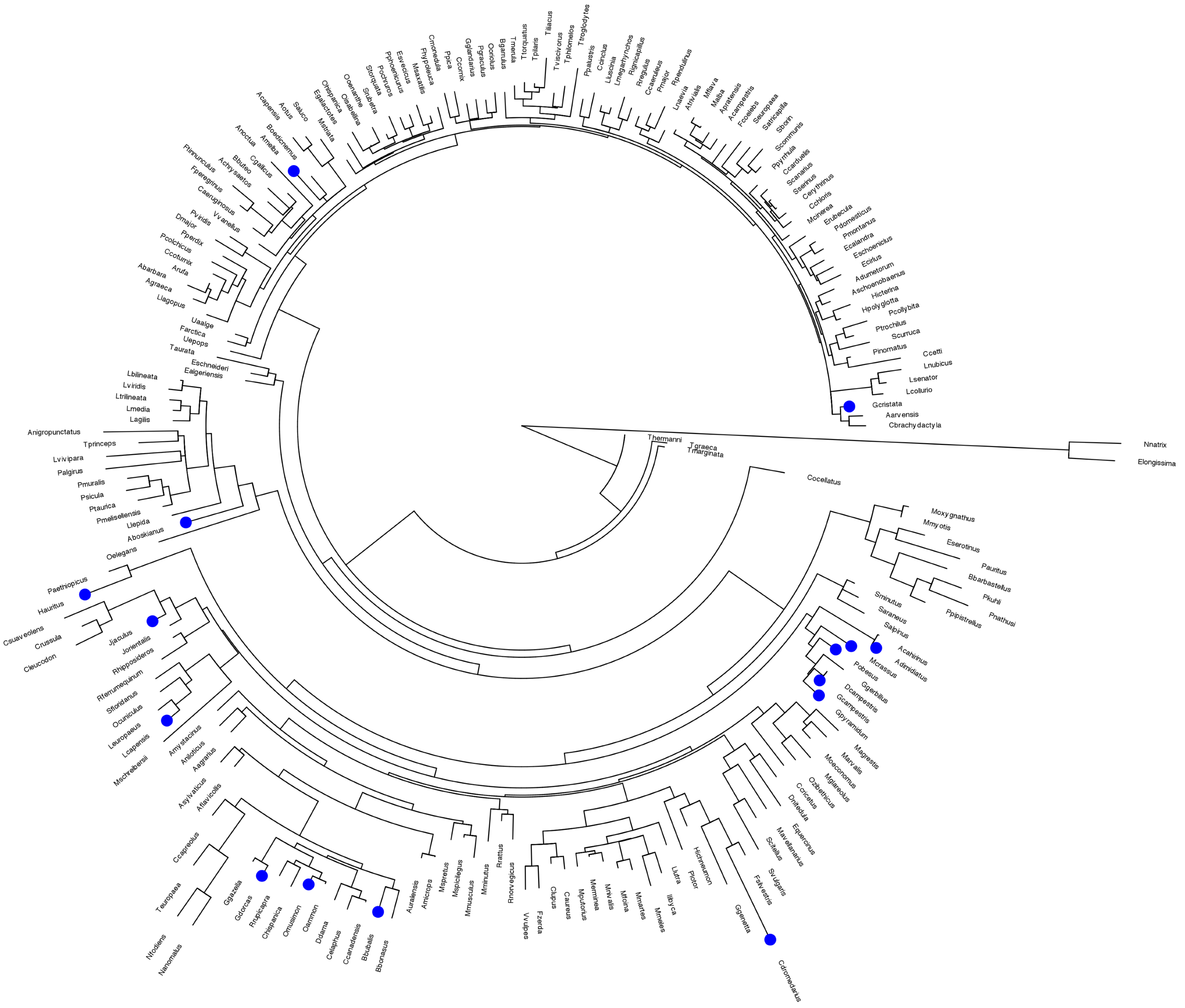

Nnatrix
Elongissima
Thermanni
Tgraeca
Tmarginata
Cocellatus
Moxygnathus
Mmyotis
Eserotinus
Pauritus
Bbarbastellus
Pkuhli
Pnathusi
Ppipistrellus
Sminutus
Saraneus
Salpinus
Acahirinus
Admidiatus
Mcrassus
Pobesus
Ggerbillus
Ocampestris
Gcampestris
Gpyramidum
Magrestis
Marvalis
Moeconomus
Mglareolus
Ozibethicus
Ccricetus
Dnitedula
Equercinus
Mavellanarius
Sciteilus
Svulgaris
Fsilvestris
Ggenetta
Cdromedarius
Hichneumon
Ptorquatus
Llibyca
Lilutra
Mmeles
Mmartes
Mfoina
Mnivalis
Mermimea
Mputorius
Caureus
Clupus
Fzerda
Vvulpes
Rnorvegicus
Rrattus
Mmiinutus
Mmusculus
Mspicilegus
Mspretus
Amicrops
Auralensis
Bbonasus
Bbubalis
Ccanadensis
Celaphus
Ddama
Oammon
Omusimon
Chispanica
Rrupicapra
Gdorcas
Ggazella
Nnanomalus
Nfodiens
Teuropaea
Ccapreolus
Aflavicollis
Asylvaticus
Aagrarius
Aniloticus
Amystacinus
Mschreibersii
Lcapensis
Leuropaeus
Ocuniculus
Sfloridanus
Rferrumequinum
Rhipposideros
Jorientalis
Jjaculus
Cleucodon
Crussula
Csuaveolens
Hauritus
Paethiopicus
Oelegans
Aboskianus
Llepida
Pmelisellensis
Ptaurica
Psicula
Pmuralis
Palgirus
Lvivipara
Tprinceps
Anigropunctatus
Lagilis
Lmedia
Ltrilineata
Lviridis
Lbilineata
Ealgeriensis
Eschneideri
Taurata
Uepops
Farctica
Uaalge
Llagopus
Agraeca
Abarbara
Arufa
Ccoturnix
Pcolchicus
Pperdix
Dmajor
Pviridis
Vvanellus
Caeruginosus
Fperegrinus
Ftinnunculus
Achrysaetos
Bbuteo
Cgallicus
Ameiba
Anoctua
Boedicnemus
Acapensis
Aotus
Saluco
Egalactotes
Mstriata
Ohispanica
Oleucura
Oseebohmi
Oenanthe
Storquata
Srubetra
Pochruros
Pphoenicurus
Esvecicus
Phypoleuca
Msaxatilis
Cmonedula
Ppica
Ccorax
Ggrlandarius
Pgraculus
Ooriolus
Bgarrulus
Tmerula
Ttorquatus
Tpilaris
Tiliacus
Tviscivorus
Tphilomelos
Ttroglodytes
Ppalustris
Ccinclus
Lluscinia
Lmegarhynchos
Rignicapillus
Rregulus
Ccaeruleus
Pmajor
Rpendulinus
Lnaevia
Atrivialis
Mflava
Malba
Apratensis
Acampestris
Fcoelebs
Seuropaea
Satricapilla
Sborin
Scommunis
Pyrrhula
Ccarduelis
Scanarius
Sserinus
Cerythrinus
Cchloris
Mcinerea
Erubecula
Pdomesticus
Pmontanus
Ecalandra
Eschoeniclus
Ecirlus
Adumetorum
Aschoenobaenus
Hicterina
Hpolyglotta
Pcollybita
Ptrochilus
Scurruca
Pinornatus
Ccetti
Lnubicus
Lsenator
Lcollurio
Gcristata
Aarvensis
Cbrachydactyla

Cluster 1

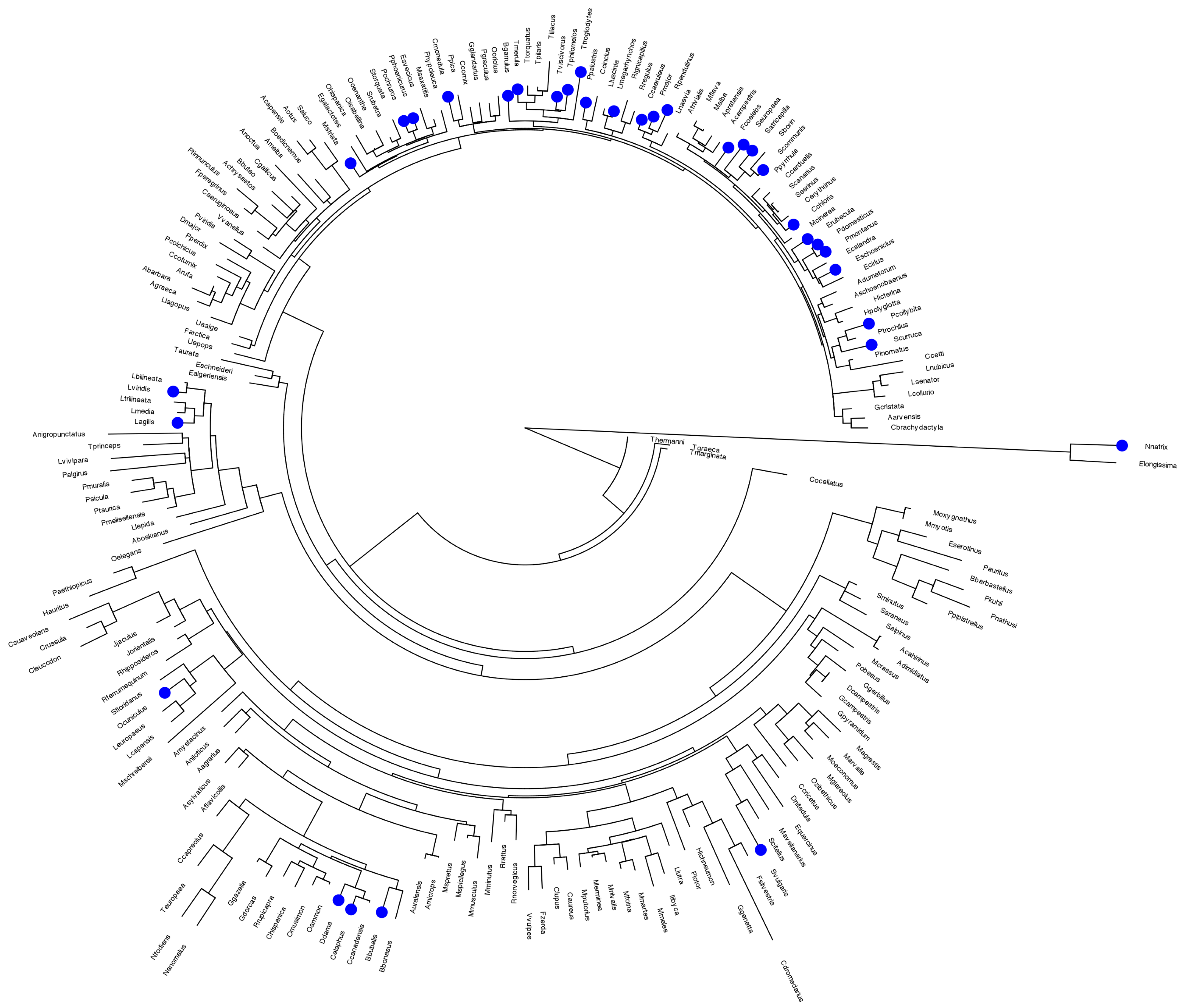
Nnatrix
Elongissima
Thermanni
Tgraeca
Tmarginata
Cocellatus
Moxygnathus
Mmyotis
Eserotinus
Pauritus
Bbarbastellus
Pkuhli
Pnathusi
Ppipistrellus
Sminutus
Saraneus
Salpinus
Acahirinus
Admidiatus
Mcrassus
Pobesus
Ggerbillus
Ocampestris
Gcampestris
Gpyramidum
Magrestis
Marvalis
Moeconomus
Mglareolus
Ozibethicus
Ccricetus
Dnitedula
Equercinus
Mavellanarius
Sciteilus
Svulgaris
Fsilvestris
Ggenetta
Cdromedarius
Hichneumon
Plotor
Llutra
Ilibyca
Mmeles
Mmartes
Mfoina
Mnivalis
Mermineа
Mputorius
Caureus
Clupus
Fzerda
Vvulpes
Rnorvegicus
Rrattus
Mminutus
Mmusculus
Mspicilegus
Mspretus
Amicrops
Auralensis
Bbonasus
Bbubalis
Ccanadensis
Celaphus
Ddama
Oammon
Omusimon
Chispanica
Rrupicapra
Gdorcas
Ggazella
Nanomalus
Nfodiens
Teuropaea
Ccapreolus
Aflavicollis
Asylvaticus
Aagrarius
Aniloticus
Amystacinus
Mschreibersii
Lcapensis
Leuropaeus
Ocuniculus
Sfloridanus
Rferrumequinum
Rhipposideros
Jorientalis
Jjaculus
Cleucodon
Crussula
Csuaveolens
Hauritus
Paethiopicus
Oelegans
Aboskianus
Llepida
Pmelisellensis
Ptaurica
Psicula
Pmuralis
Palgirus
Lvivipara
Tprinceps
Anigropunctatus
Lagilis
Lmedia
Ltrilineata
Lviridis
Lbilineata
Ealgeriensis
Eschneideri
Taurata
Uepops
Farctica
Uaalge
Llagopus
Agraeca
Abarbara
Arufa
Ccoturnix
Pcolchicus
Pperdix
Dmajor
Pviridis
Vvanellus
Caeruginosus
Fperegrinus
Ftinnunculus
Achrysaetos
Bbuteo
Cgallicus
Anoctua
Ameiba
Boedicnemus
Acapensis
Aotus
Saluco
Egalactotes
Mstriata
Ohispanica
Oleucura
Oenanthe
Ssubetra
Storquata
Pochruros
Pphoenicurus
Esvecicus
Msaxatilis
Fhypoleuca
Cmonedula
Ppica
Ccorax
Gglandarius
Pgraculus
Ooriolus
Bgarrulus
Tmerula
Ttorquatus
Tpilaris
Tiliacus
Tviscivorus
Tphilomelos
Ttroglodytes
Ppalustris
Ccinclus
Lluscinia
Lmegarhynchos
Rignicapillus
Rregulus
Ccaeruleus
Pmajor
Rpendulinus
Lnaevia
Atrivialis
Mflava
Malba
Apratensis
Acampestris
Fcoelebs
Seuropaea
Satricapilla
Sborin
Scommunis
Ppyrrhula
Ccarduelis
Scanarius
Sserinus
Cerythrinus
Cchloris
Mcinerea
Erubecula
Pdomesticus
Pmontanus
Ecalandra
Eschoeniclus
Ecirlus
Adumetorum
Aschoenobaenus
Hicterina
Hpolyglotta
Pcollybita
Ptrochilus
Scurruca
Pinornatus
Ccetti
Lnubicus
Lsenator
Lcollurio
Gcristata
Aarvensis
Cbrachydactyla

Cluster 2

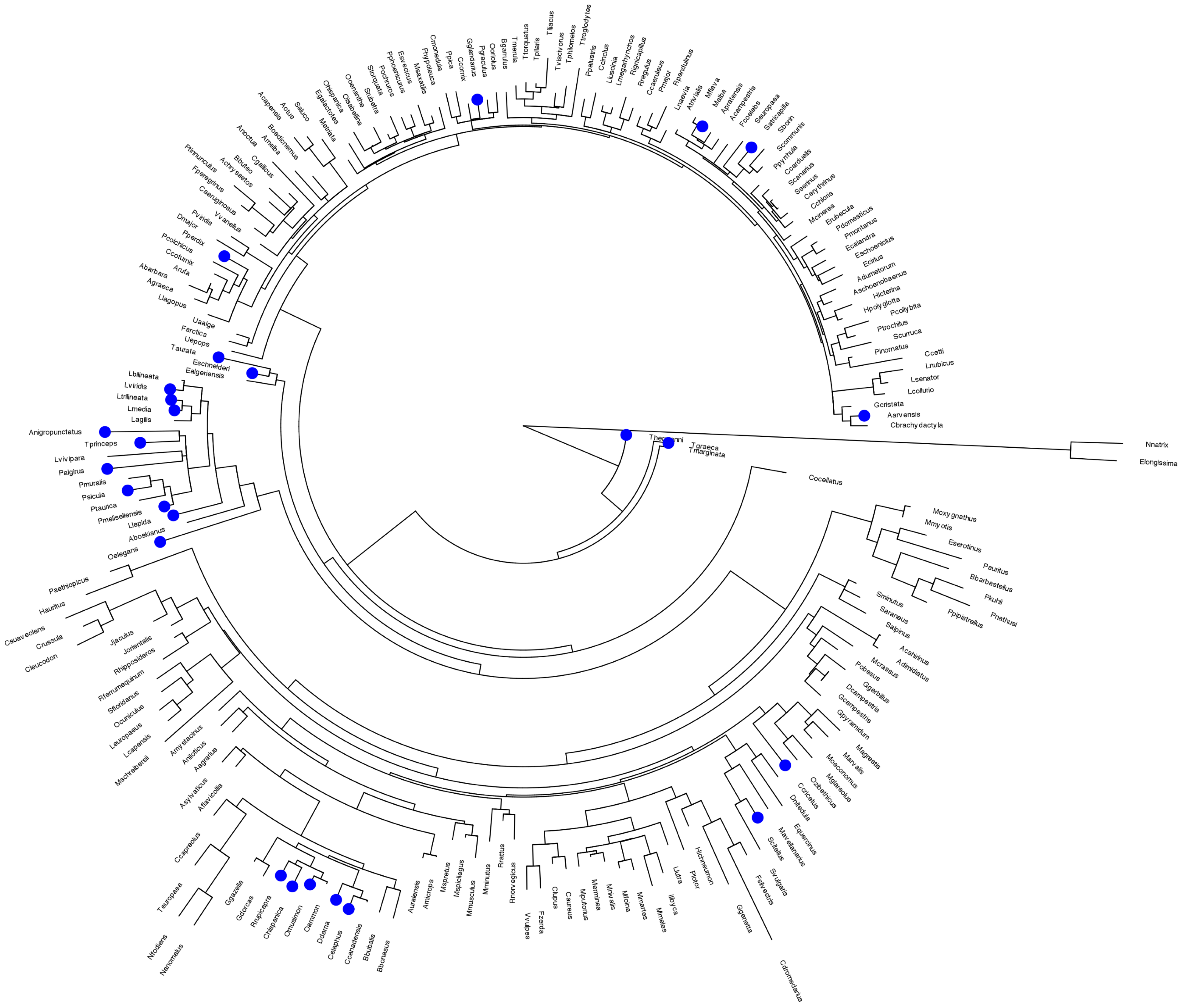
Nnatrix
Elongissima
Cocellatus
Moxygnathus
Mmyotis
Eserotinus
Pauritus
Bbarbastellus
Pkuhli
Pnathusi
Ppipistrellus
Sminutus
Saraneus
Salpinus
Acahirinus
Admidiatus
Mcrassus
Pobesus
Ggerbillus
Ocampestris
Gcampestris
Gpyramidum
Magrestis
Marvalis
Moeconomus
Mglareolus
Ozibethicus
Ccricetus
Dnitedula
Equercinus
Mavellanarius
Scitellus
Svulgaris
Fsilvestris
Ggenetta
Cdromedarius
Hichneumon
Lllutra
Ilibyca
Mmeles
Mmartes
Mfoina
Mnivalis
Merminea
Mputorius
Caureus
Clupus
Fzerda
Vvulpes
Rnorvegicus
Rrattus
Mminutus
Mmusculus
Mspicilegus
Mspretus
Amicrops
Auralensis
Bbonasus
Bbubalis
Ccanadensis
Celaphus
Ddama
Oammon
Omusimon
Chispanica
Rrupicapra
Gdorcas
Ggazella
Nanomalus
Nfodiens
Teuropaea
Ccapreolus
Aflavicollis
Asylvaticus
Aagrarius
Aniloticus
Amystacinus
Mschreibersii
Lcapensis
Leuropaeus
Ocuniculus
Sfloridanus
Rferrumequinum
Rhipposideros
Jorientalis
Jjaculus
Cleucodon
Crussula
Csuaveolens
Hauritus
Paethiopicus
Oelegans
Aboskianus
Llepida
Pmelisellensis
Ptaurica
Psicula
Pmuralis
Palgirus
Lvivipara
Tprinceps
Anigropunctatus
Lagilis
Lmedia
Ltrilineata
Lviridis
Lbilineata
Ealgeriensis
Eschneideri
Taurata
Uepops
Farctica
Uaalge
Llagopus
Agraeca
Abarbara
Arufa
Ccoturnix
Pcolchicus
Pperdix
Dmajor
Pviridis
Vvanellus
Caeruginosus
Fperegrinus
Ftinnunculus
Achrysaetos
Bbuteo
Cgallicus
Anoctua
Boedicnemus
Aotus
Saluco
Mstriata
Oenanthe
Storquata
Srubetra
Pochruros
Pphoenicurus
Esvecica
Msaxatilis
Phypoleuca
Cmonedula
Ppica
Ccorax
Gglandarius
Pgraculus
Ooriolus
Bgarrulus
Tmerula
Ttorquatus
Tpilaris
Tiliacus
Tviscivorus
Tphilomelos
Ttroglodytes
Ppalustris
Ccinclus
Lluscinia
Lmegarhynchos
Rignicapillus
Rregulus
Ccaeruleus
Pmajor
Rpendulinus
Lnaevia
Atrivialis
Mflava
Malba
Apratensis
Acampestris
Fcoelebs
Seuropaea
Satricapilla
Sborin
Scommunis
Ppyrrhula
Ccarduelis
Scanarius
Sserinus
Cerythrinus
Cchloris
Mcinerea
Erubecula
Pdomesticus
Pmontanus
Ecalandra
Eschoeniclus
Ecirlus
Adumetorum
Aschoenobaenus
Hicterina
Hpolyglotta
Pcollybita
Ptrochilus
Scurruca
Pinornatus
Ccetti
Lnubicus
Lsenator
Lcollurio
Gcristata
Aarvensis
Cbrachydactyla
Tgraeca
Tmarginata

Cluster 3

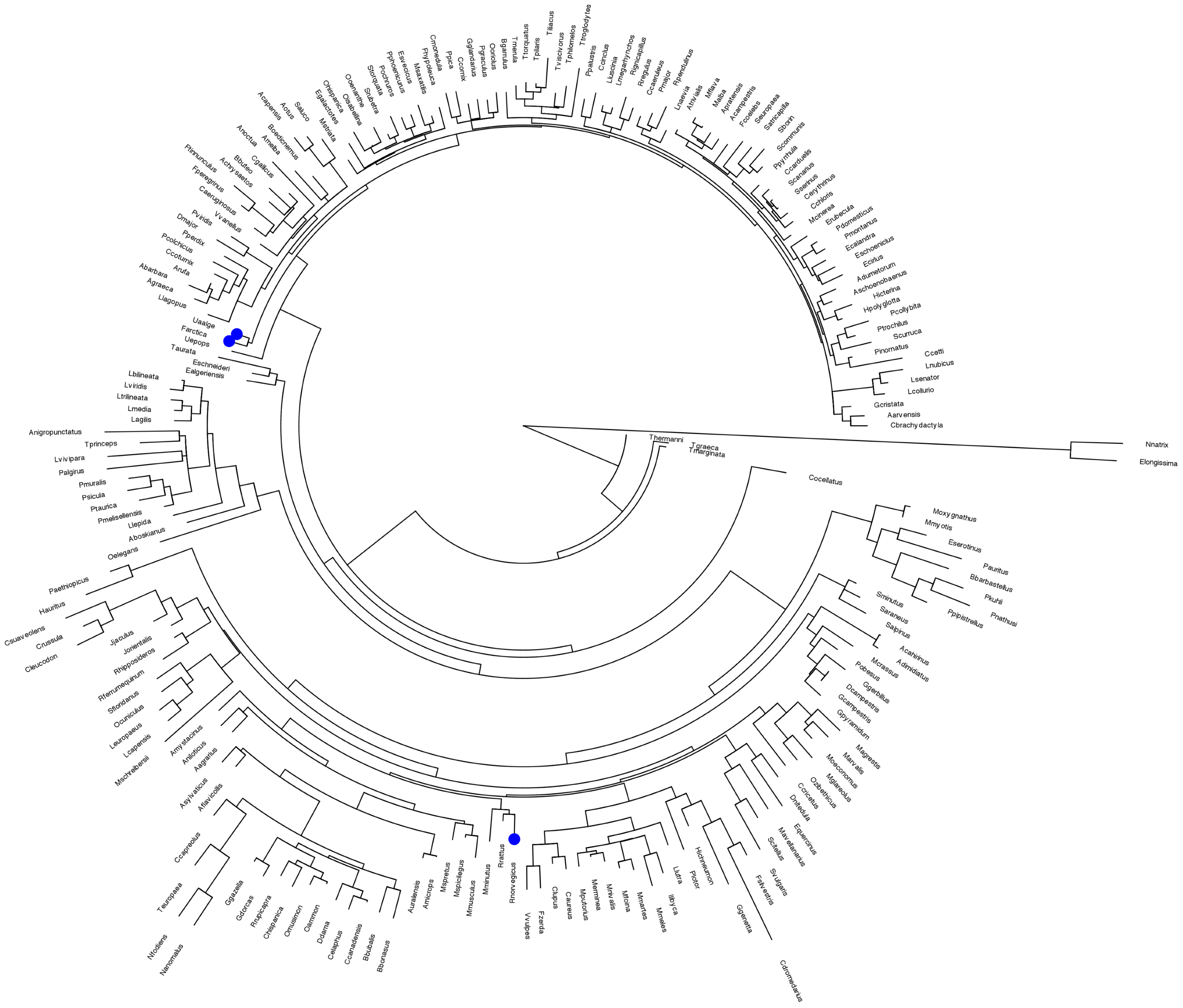
Ttroglodytes
Tiliacus
Ttorquatus
Tpilaris
Tviscivorus
Tphilomelos
Tmerula
Bgarrulus
Ooriolus
Pgraculus
Gglandarius
Ccorax
Ppica
Cmonedula
Phypoleuca
Msaxatilis
Esvecicus
Pphoenicurus
Pochruros
Storquata
Srubetra
Ooenanthe
Ohispanica
Oisabellina
Egalactotes
Mstriata
Acapensis
Aotus
Saluco
Boedicnemus
Anoctua
Amelba
Cgallicus
Bbuteo
Achrysaetos
Ftinnunculus
Fperegrinus
Caeruginosus
Vvanellus
Pviridis
Dmajor
Pperdix
Pcolchicus
Ccoturnix
Arufa
Abarbara
Agraeca
Llagopus
Uaalge
Farctica
Uepops
Taurata
Eschneideri
Ealgeriensis
Lbilineata
Lviridis
Ltrilineata
Lmedia
Lagilis
Anigropunctatus
Tprinceps
Lvivipara
Palgirus
Pmuralis
Psicula
Ptaurica
Pmelisellensis
Llepida
Aboskianus
Oelegans
Paethiopicus
Hauritus
Csuaveolens
Crussula
Cleucodon
Jjaculus
Jorientalis
Rhipposideros
Rferrumequinum
Sfloridanus
Ocuniculus
Leuropaeus
Lcapensis
Mschreibersii
Amystacinus
Aniloticus
Aagrarius
Asylvaticus
Aflavicollis
Ccapreolus
Teuropaea
Nfodiens
Nanomalus
Ggazella
Gdorcas
Rrupicapra
Chispanica
Omusimon
Oammon
Ddama
Celaphus
Ccanadensis
Bbubalis
Bbonasus
Auralensis
Amicrops
Mspretus
Mspicilegus
Mmusculus
Mminutus
Rrattus
Rnorvegicus
Vvulpes
Fzerda
Clupus
Caureus
Mputorius
Merminea
Mnivalis
Mfoina
Mmartes
Mmeles
Llutra
Flibyca
Hichneumon
Plotor
Ggenetta
Fsilvestris
Cdromedarius
Svulgaris
Scitellus
Mavellanarius
Equercinus
Dnitedula
Ccricetus
Ozibethicus
Mglareolus
Moeconomus
Marvalis
Magrestis
Gpyramidum
Gcampestris
Dcampestris
Ggerbillus
Pobesus
Mcrassus
Adimidiatus
Acahirinus
Salpinus
Saraneus
Sminutus
Ppipistrellus
Pnathusi
Pkuhli
Bbarbastellus
Pauritus
Eserotinus
Mmyotis
Moxygnathus
Cocellatus
Thermanni
Tgraeca
Tmarginata
Nnatrix
Elongissima
Cbrachydactyla
Aarvensis
Gcristata
Lcollurio
Lsenator
Lnubicus
Ccetti
Pinornatus
Scurruca
Ptrochilus
Pcollybita
Hpolyglotta
Hicterina
Aschoenobaenus
Adumetorum
Ecirlus
Eschoeniclus
Ecalandra
Pmontanus
Pdomesticus
Erubecula
Mcinerea
Cchloris
Cerythrinus
Sserinus
Scanarius
Ccarduelis
Ppyrrhula
Scommunis
Sborin
Satricapilla
Seuropaea
Fcoelebs
Acampestris
Apratensis
Malba
Mflava
Atrivialis
Lnaevia
Rpendulinus
Pmajor
Ccaeruleus
Rregulus
Rignicapillus
Lmegarhynchos
Lsvecica
Ccinclus
Ppalustris

Cluster 4

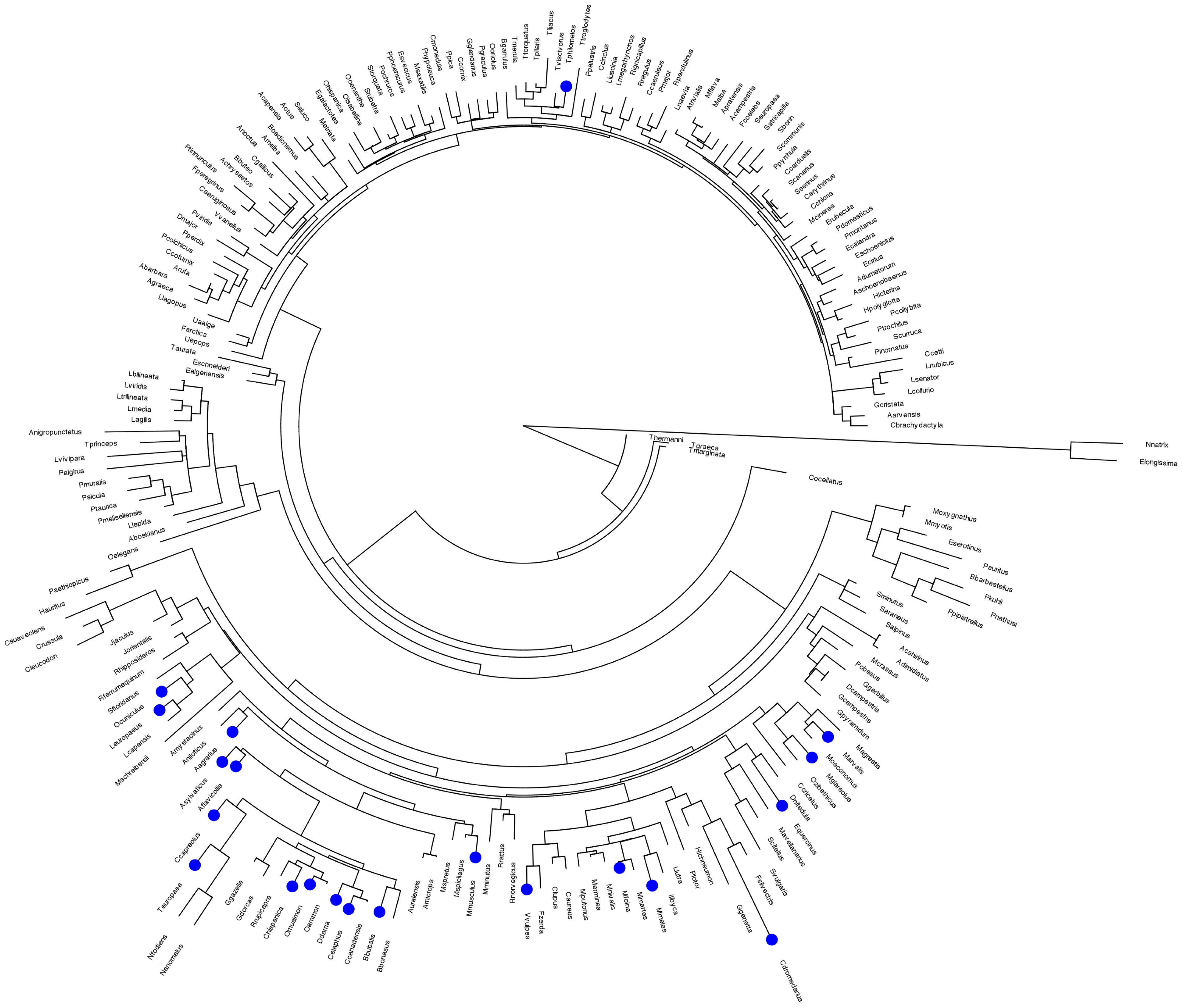

Tiliacus
Ttroglodytes
Tphilomelos
Tviscivorus
Tpilaris
Ttorquatus
Tmerula
Bgarrulus
Ooriolus
Pgraculus
Gglandarius
Ccorax
Ppica
Cmonedula
Phypoleuca
Msaxatilis
Esyriacus
Pphoenicurus
Pochruros
Storquata
Srubetra
Oenanthe
Ohispanica
Osseab
Egalactotes
Mstriata
Acapensis
Aotus
Saluco
Boedicnemus
Anoctua
Ameiba
Cgallicus
Btinnunculus
Bbuteo
Achrysaetos
Fperegrinus
Caeruginosus
Vvanellus
Pviridis
Dmajor
Pperdix
Pcolchicus
Ccoturnix
Arufa
Abarbara
Agraeca
Llagopus
Uaalge
Farctica
Uepops
Taurata
Eschneideri
Ealgeriensis
Lbilineata
Lviridis
Ltrilineata
Lmedia
Lagilis
Anigropunctatus
Tprinceps
Lvivipara
Palgirus
Pmuralis
Psicula
Ptaurica
Pmelisellensis
Llepida
Aboskianus
Oelegans
Paethiopicus
Hauritus
Csuaveolens
Crussula
Cleucodon
Jjaculus
Jorientalis
Rhipposideros
Rferrumequinum
Sfloridanus
Ocuniculus
Leuropaeus
Lcapensis
Mschreibersii
Amystacinus
Aniloticus
Aagrarius
Asylvaticus
Aflavicollis
Ccapreolus
Teuropaea
Nfodiens
Nanomalus
Ggazella
Gdorcas
Rrupicapra
Chispanica
Omusimon
Oammon
Ddama
Celaphus
Ccanadensis
Bbubalis
Bbonasus
Auralensis
Amicrops
Mspretus
Mspicilegus
Mmusculus
Mminutus
Rrattus
Rnorvegicus
Vvulpes
Fzerda
Clupus
Caureus
Mputorius
Merminea
Mnivalis
Mfoina
Mmartes
Mmeles
Llutra
Ilibyca
Hichneumon
Plotor
Ggenetta
Cdromedarius
Fsilvestris
Svulgaris
Scitellus
Mavellanarius
Equercinus
Dnitedula
Ccricetus
Mglareolus
Ocabethicus
Moeconomus
Marvalis
Magrestis
Gpyramidum
Gcampestris
Ocampestris
Ggerbillus
Pobesus
Mcrassus
Admidiatus
Acahirinus
Salpinus
Saraneus
Sminutus
Ppipistrellus
Pnathusi
Pkuhli
Bbarbastellus
Pauritus
Eserotinus
Mmyotis
Moxygnathus
Cocellatus
Thermanni
Tgraeca
Tmarginata
Nnatrix
Elongissima
Cbrachydactyla
Aarvensis
Gcristata
Lcollurio
Lsenator
Lnubicus
Ccetti
Pinornatus
Scurruca
Ptrochilus
Pcollybita
Hpolyglotta
Hicterina
Aschoenobaenus
Adumetorum
Ecirlus
Eschoeniclus
Ecalandra
Pmontanus
Pdomesticus
Erubecula
Mcinerea
Cchloris
Cerythrinus
Sserinus
Scanarius
Ccarduelis
Ppyrrhula
Scommunis
Sborin
Satricapilla
Seuropaea
Fcoelebs
Acampestris
Apratensis
Malba
Mflava
Atrivialis
Lnaevia
Rpendulinus
Pmajor
Ccaeruleus
Rregulus
Rignicapillus
Lmegarhynchos
Lluscinia
Ccinclus
Ppalustris

Cluster 5

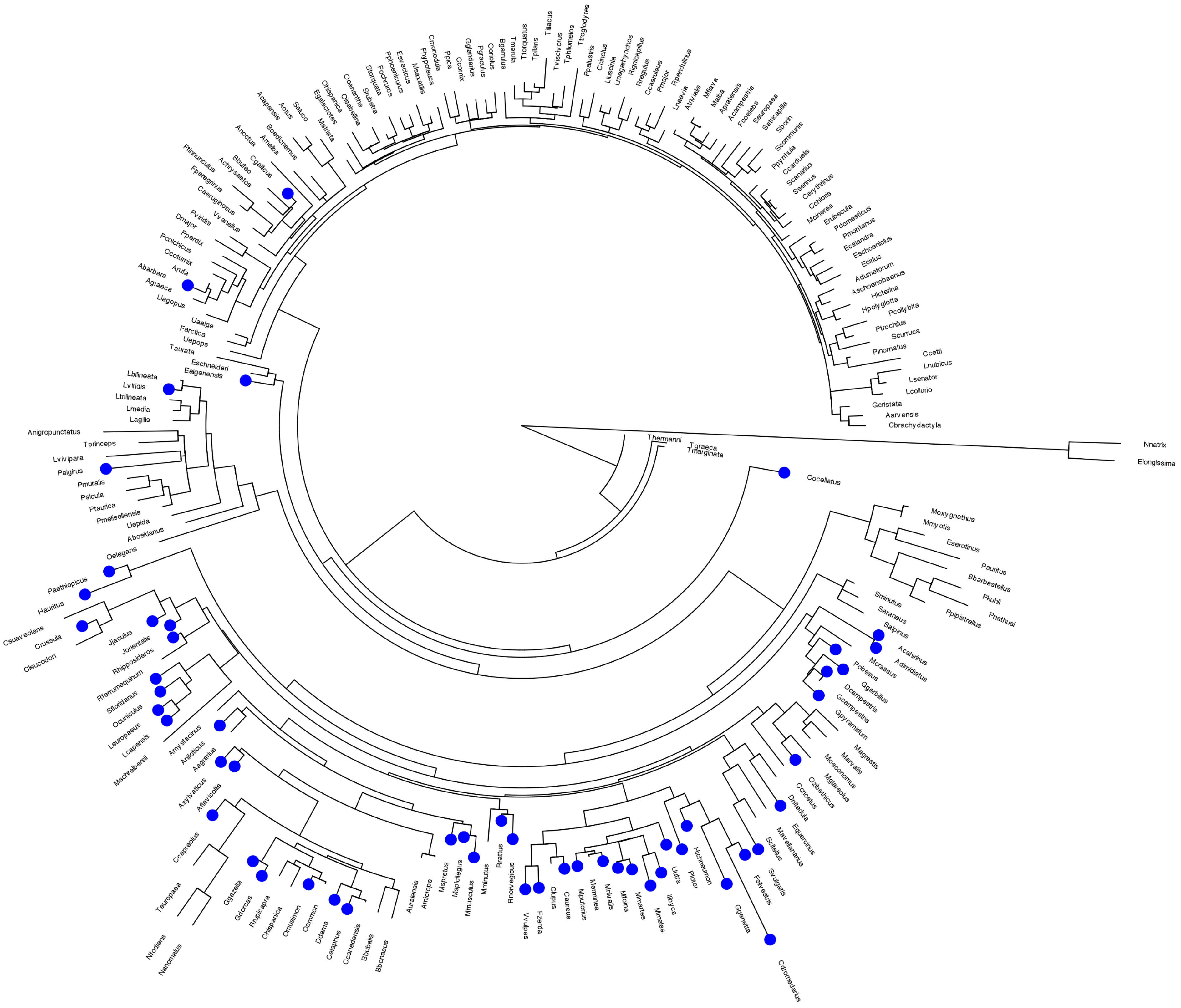

Ttroglodytes
Tiliacus
Tphilomelos
Tviscivorus
Tpilaris
Ttorquatus
Tmerula
Bgarrulus
Ooriolus
Pgraculus
Gglandarius
Ccorax
Ppica
Cmonedula
Phypoleuca
Msaxatilis
Esvecicus
Pphoenicurus
Pochruros
Srubetra
Storquata
Oisabellina
Oenanthe
Ohispanica
Egalactotes
Mstriata
Salucco
Aotus
Acapensis
Boedicnemus
Anoctua
Ameiba
Cgallicus
Bbuteo
Achrysaetos
Ftinnunculus
Fperegrinus
Caeruginosus
Vvanellus
Pviridis
Dmajor
Pperdix
Pcolchicus
Ccoturnix
Arufa
Abarbara
Agraeca
Llagopus
Uaalge
Farctica
Uepops
Taurata
Eschneideri
Ealgeriensis
Lbilineata
Lviridis
Ltrilineata
Lmedia
Lagilis
Anigropunctatus
Tprinceps
Lvivipara
Palgirus
Pmuralis
Psicula
Ptaurica
Pmelisellensis
Llepida
Aboskianus
Oelegans
Paethiopicus
Hauritus
Csuaveolens
Crussula
Cleucodon
Jjaculus
Jorientalis
Rhipposideros
Rferrumequinum
Sfloridanus
Ocuniculus
Leuropaeus
Lcapensis
Mschreibersii
Amystacinus
Aniloticus
Aagrarius
Asylvaticus
Aflavicollis
Ccapreolus
Teuropaea
Nfodiens
Nanomalus
Ggazella
Gdorcas
Rrupicapra
Chispanica
Omusimon
Oammon
Ddama
Celaphus
Ccanadensis
Bbubalis
Bbonasus
Auralensis
Amicrops
Mspretus
Mspicilegus
Mmusculus
Mminutus
Rrattus
Rnorvegicus
Vvulpes
Fzerda
Clupus
Caureus
Mputorius
Merminea
Mnivalis
Mfoina
Mmartes
Mmeles
llibyca
Llutra
Plotor
Hichneumon
Ggenetta
Cdromedarius
Fsilvestris
Svulgaris
Scitellus
Mavellanarius
Equercinus
Dnitedula
Ccricetus
Ozibethicus
Mglareolus
Moeconomus
Marvalis
Magrestis
Gpyramidum
Gcampestris
Ocampestris
Ggerbillus
Pobesus
Mcrassus
Admidiatus
Acahirinus
Salpinus
Saraneus
Sminutus
Pnathusi
Ppipistrellus
Pkuhli
Bbarbastellus
Pauritus
Eserotinus
Mmyotis
Moxygnathus
Cocellatus
Nnatrix
Elongissima
Thermanni
Tgraeca
Tmarginata
Cbrachydactyla
Aarvensis
Gcristata
Lcollurio
Lsenator
Lnubicus
Ccetti
Pinornatus
Scurruca
Ptrochilus
Pcollybita
Hpolyglotta
Hicterina
Aschoenobaenus
Adumetorum
Ecirlus
Eschoeniclus
Ecalandra
Pmontanus
Pdomesticus
Erubecula
Mcinerea
Cchloris
Cerythrinus
Sserinus
Scanarius
Ccarduelis
Ppyrrhula
Scommunis
Sborin
Satricapilla
Seuropaea
Fcoelebs
Acampestris
Apratensis
Malba
Mflava
Atrivialis
Lnaevia
Rpendulinus
Pmajor
Ccaeruleus
Rregulus
Rignicapillus
Lmegarhynchos
Lluscinia
Ccinclus
Ppalustris

Cluster 6

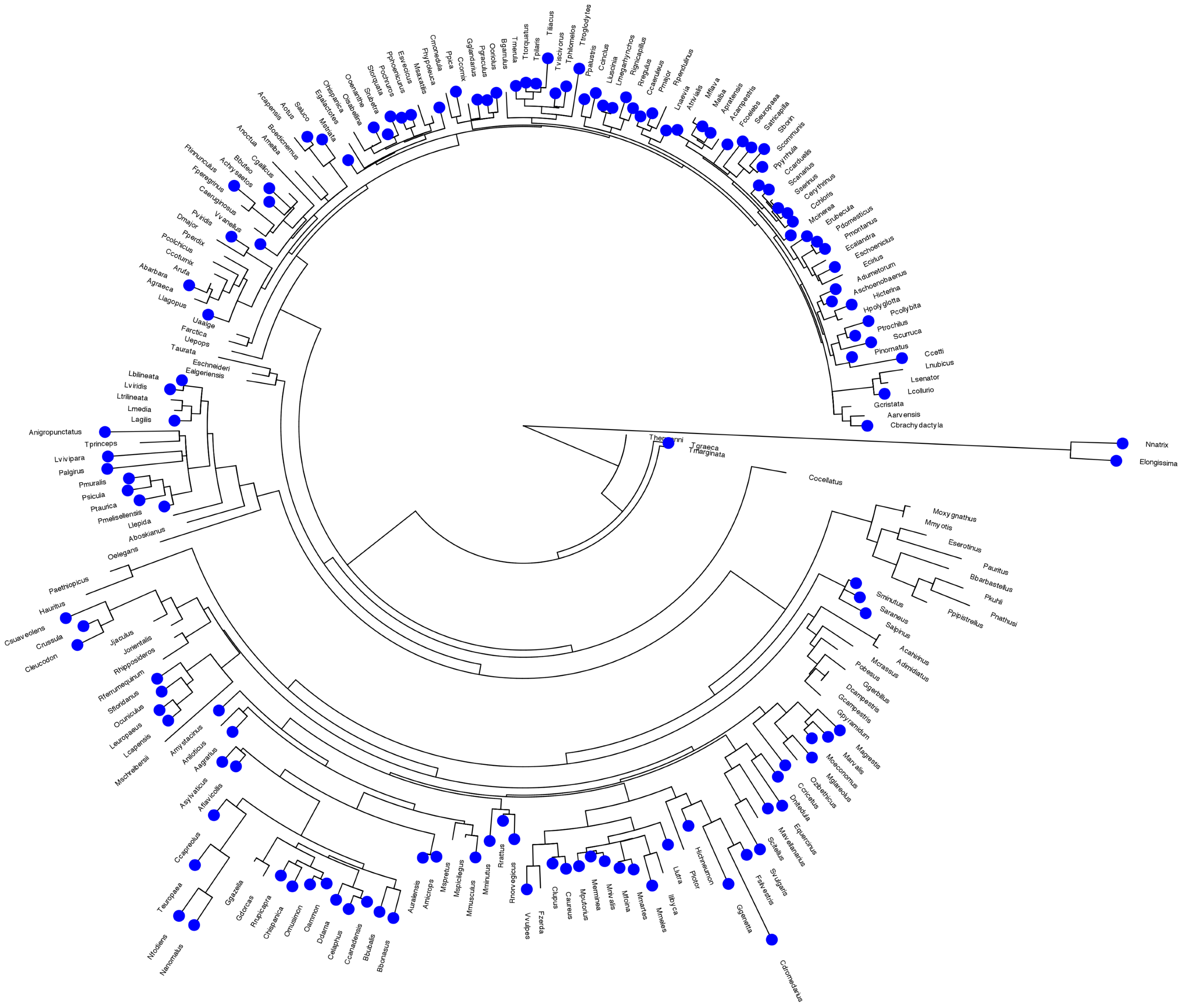

Nnatrix
Elongissima
Tgraeca
Tmarginata
Cocellatus
Moxygnathus
Mmyotis
Eserotinus
Pauritus
Bbarbastellus
Pkuhli
Pnathusi
Ppipistrellus
Sminutus
Saraneus
Salpinus
Acahirinus
Adimidiatus
Mcrassus
Pobesus
Ggerbillus
Ocampestris
Gcampestris
Gpyramidum
Magrestis
Marvalis
Moeconomus
Mglareolus
Ozibethicus
Ccricetus
Dnitedula
Equercinus
Mavellanarius
Scitellus
Svulgaris
Fsilvestris
Ggenetta
Cdromedarius
Hichneumon
Llutra
Llibyca
Mmeles
Mmartes
Mfoina
Mnivalis
Merminea
Mputorius
Caureus
Clupus
Fzerda
Vvulpes
Rnorvegicus
Rrattus
Mminutus
Mmusculus
Mspicilegus
Mspretus
Amicrops
Auralensis
Bbonasus
Bbubalis
Ccanadensis
Celaphus
Ddama
Oammon
Omusimon
Chispanica
Rrupicapra
Gdorcas
Ggazella
Nanomalus
Nfodiens
Teuropaea
Ccapreolus
Asylvaticus
Aflavicollis
Aagrarius
Aniloticus
Amystacinus
Mschreibersii
Lcapensis
Leuropaeus
Ocuniculus
Sfloridanus
Rferrumequinum
Rhipposideros
Jorientalis
Jjaculus
Cleucodon
Crussula
Csuaveolens
Hauritus
Paethiopicus
Oelegans
Aboskianus
Llepida
Pmelisellensis
Ptaurica
Psicula
Pmuralis
Palgirus
Lvivipara
Tprinceps
Anigropunctatus
Lagilis
Lmedia
Ltrilineata
Lviridis
Lbilineata
Ealgeriensis
Eschneideri
Taurata
Uepops
Farctica
Uaalge
Llagopus
Agraeca
Abarbara
Arufa
Ccoturnix
Pcolchicus
Pperdix
Dmajor
Pviridis
Vvanellus
Caeruginosus
Fperegrinus
Ftinnunculus
Achrysaetos
Bbuteo
Cgallicus
Anoctua
Amelba
Boedicnemus
Acapensis
Aotus
Saluco
Egalactotes
Ohispanica
Mstriata
Oenanthe
Storquata
Srubetra
Pochruros
Pphoenicurus
Esvecicus
Fhypoleuca
Msaxatilis
Cmonedula
Ppica
Ccornix
Gglandarius
Pgraculus
Ooriolus
Bgarrulus
Tmerula
Ttorquatus
Tpilaris
Tiliacus
Tviscivorus
Tphilomelos
Ttroglodytes
Ppalustris
Ccinclus
Lluscinia
Lmegarhynchos
Rignicapillus
Rregulus
Ccaeruleus
Pmajor
Rpendulinus
Lnaevia
Atrivialis
Mflava
Malba
Apratensis
Acampestris
Fcoelebs
Seuropaea
Satricapilla
Sborin
Scommunis
Ppyrrhula
Ccarduelis
Scanarius
Sserinus
Cerythrinus
Cchloris
Mcinerea
Erubecula
Pdomesticus
Pmontanus
Ecalandra
Eschoeniclus
Ecirlus
Adumetorum
Aschoenobaenus
Hicterina
Hpolyglotta
Pcollybita
Ptrochilus
Scurruca
Pinornatus
Ccetti
Lnubicus
Lsenator
Lcollurio
Gcristata
Aarvensis
Cbrachydactyla

Cluster 9

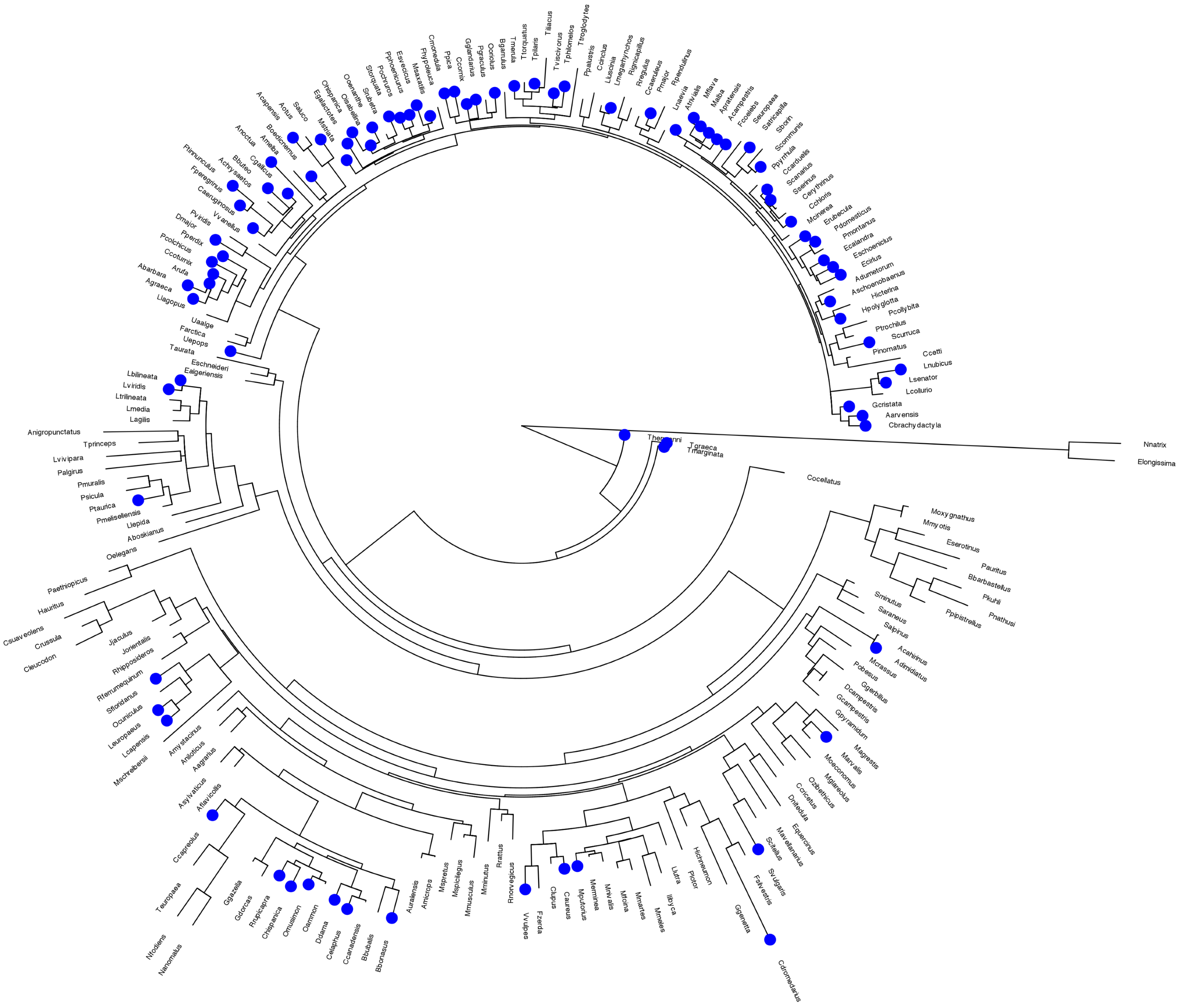

Nnatrix
Elongissima
Thermanni
Tgraeca
Tmarginata
Cocellatus
Moxygnathus
Mmyotis
Eserotinus
Pauritus
Bbarbastellus
Pkuhli
Pnathusi
Ppipistrellus
Sminutus
Saraneus
Salpinus
Acahirinus
Adimidiatus
Mcrassus
Pobesus
Ggerbillus
Ocampestris
Gcampestris
Gpyramidum
Magrestis
Marvalis
Moeconomus
Mglareolus
Ccricetus
Dnitedula
Equercinus
Mavellanarius
Svulgaris
Fsilvestris
Ggenetta
Cdromedarius
Hichneumon
Llutra
Mmeles
Mmartes
Mfoina
Mnivalis
Merminea
Mputorius
Caureus
Clupus
Fzerda
Vvulpes
Rnorvegicus
Rrattus
Mminutus
Mmusculus
Mspicilegus
Mspretus
Auralensis
Bbonasus
Bbubalis
Ccanadensis
Celaphus
Ddama
Oammon
Omusimon
Chispanica
Rrupicapra
Gdorcas
Ggazella
Nanomalus
Nfodiens
Teuropaea
Ccapreolus
Aflavicollis
Asylvaticus
Aagrarius
Amystacinus
Mschreibersii
Lcapensis
Leuropaeus
Ocuniculus
Sfloridanus
Rferrumequinum
Rhipposideros
Jorientalis
Jjaculus
Cleucodon
Crussula
Csuaveolens
Hauritus
Paethiopicus
Oelegans
Aboskianus
Llepida
Pmelisellensis
Ptaurica
Psicula
Pmuralis
Palgirus
Lvivipara
Tprinceps
Anigropunctatus
Lagilis
Lmedia
Ltrilineata
Lviridis
Lbilineata
Ealgeriensis
Eschneideri
Taurata
Uepops
Farctica
Uaalge
Llagopus
Agraeca
Abarbara
Arufa
Ccoturnix
Pcolchicus
Pperdix
Dmajor
Pviridis
Vvanellus
Caeruginosus
Fperegrinus
Ftinnunculus
Achrysaetos
Bbuteo
Cgallicus
Anoctua
Boedicnemus
Acapensis
Aotus
Saluco
Egalactotes
Ohispanica
Oisabellina
Ooenanthe
Storquata
Pphoenicurus
Msaxatilis
Phypoleuca
Cmonedula
Ppica
Ccornix
Gglandarius
Pgraculus
Ooriolus
Bgarrulus
Tmerula
Ttorquatus
Tpilaris
Tiliacus
Tviscivorus
Tphilomelos
Ttroglodytes
Ppalustris
Ccinclus
Lluscinia
Lmegarhynchos
Rignicapillus
Rregulus
Ccaeruleus
Pmajor
Rpendulinus
Lnaevia
Atrivialis
Mflava
Malba
Apratensis
Acampestris
Fcoelebs
Seuropaea
Scommunis
Ccarduelis
Scanarius
Sserinus
Cerythrinus
Cchloris
Mcinerea
Erubecula
Pdomesticus
Pmontanus
Ecalandra
Eschoeniclus
Ecirlus
Adumetorum
Aschoenobaenus
Hicterina
Hpolyglotta
Pcollybita
Ptrochilus
Scurruca
Ccetti
Lnubicus
Lsenator
Lcollurio
Gcristata
Aarvensis
Cbrachydactyla

Cluster 10

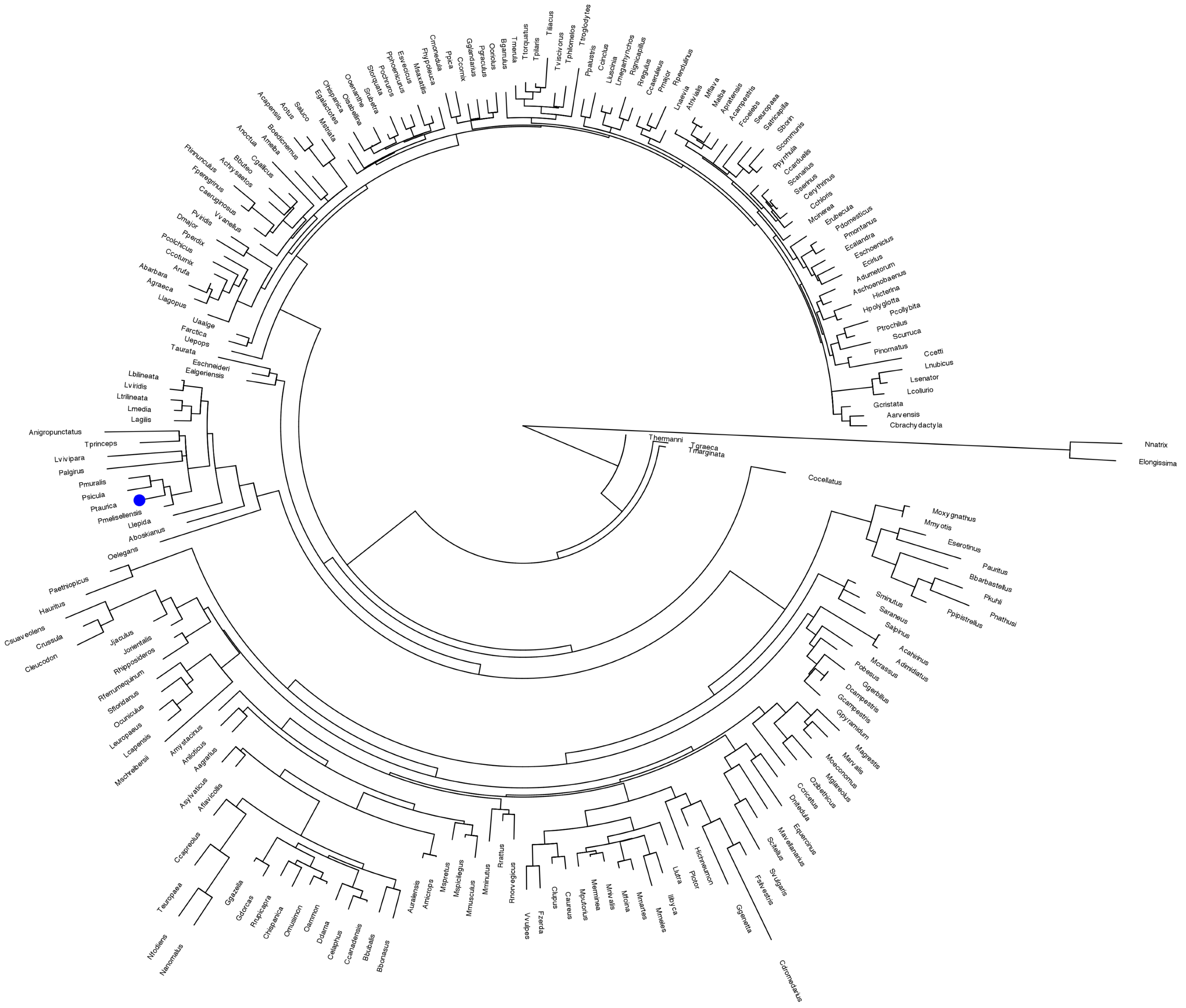

Nnatrix
Elongissima
Thermanni
Tgraeca
Tmarginata
Cocellatus
Moxygnathus
Mmyotis
Eserotinus
Pauritus
Bbarbastellus
Pkuhli
Pnathusi
Ppipistrellus
Sminutus
Saraneus
Salpinus
Acahirinus
Adimidiatus
Mcrassus
Pobesus
Ggerbillus
Dcampestris
Gcampestris
Gpyramidum
Magrestis
Marvalis
Moeconomus
Mglareolus
Ozibethicus
Ccricetus
Dnitedula
Equercinus
Mavellanarius
Sciteilus
Svulgaris
Fsilvestris
Ggenetta
Cdromedarius
Hichneumon
Ptorio
Llutra
Ilibyca
Mmeles
Mmartes
Mfoina
Mnivalis
Mermimea
Mputorius
Caureus
Clupus
Fzerda
Vvulpes
Rnorvegicus
Rrattus
Mminutus
Mmusculus
Mspicilegus
Mspretus
Amicrops
Auralensis
Bbonasus
Bbubalis
Ccanadensis
Celaphus
Ddama
Oammon
Omusimon
Chispanica
Rrupicapra
Gdorcas
Ggazella
Nanomalus
Nfodiens
Teuropaea
Ccapreolus
Aflavicollis
Asylvaticus
Aagrarius
Aniloticus
Amystacinus
Mschreibersii
Lcapensis
Leuropaeus
Ocuniculus
Sfloridanus
Rferrumequinum
Rhipposideros
Jorientalis
Jjaculus
Cleucodon
Crussula
Csuaveolens
Hauritus
Paethiopicus
Oelegans
Aboskianus
Llepida
Pmelisellensis
Ptaurica
Psicula
Pmuralis
Palgirus
Lvivipara
Tprinceps
Anigropunctatus
Lagilis
Lmedia
Ltrilineata
Lviridis
Lbilineata
Ealgeriensis
Eschneideri
Taurata
Uepops
Farctica
Uaalge
Llagopus
Agraeca
Abarbara
Arufa
Ccoturnix
Pcolchicus
Pperdix
Dmajor
Pviridis
Vvanellus
Caeruginosus
Fperegrinus
Ftinnunculus
Achrysaetos
Bbuteo
Cgallicus
Anoctua
Ameiba
Boedicnemus
Acapensis
Aotus
Saluco
Egallactotes
Ohispanica
Mstriata
Oenanthe
Otesabellina
Storquata
Srubetra
Pochruros
Pphoenicurus
Esvecicus
Msaxatilis
Phypoleuca
Cmonedula
Ppica
Ccornix
Gglandarius
Pgraculus
Ooriolus
Bgarrulus
Tmerula
Ttorquatus
Tpilaris
Tiliacus
Tviscivorus
Tphilomelos
Ttroglodytes
Ppalustris
Ccinclus
Lluscinia
Lmegarhynchos
Rignicapillus
Rregulus
Ccaeruleus
Pmajor
Rpendulinus
Lnaevia
Atrivialis
Mflava
Malba
Apratensis
Acampestris
Fcoelebs
Seuropaea
Satricapilla
Sborin
Scommunis
Ppyrrhula
Ccarduelis
Scanarius
Sserinus
Cerythrinus
Cchloris
Mcinerea
Erubecula
Pdomesticus
Pmontanus
Ecalandra
Eschoeniclus
Ecirlus
Adumetorum
Aschoenobaenus
Hicterina
Hpolyglotta
Pcollybita
Ptrochilus
Scurruca
Pinornatus
Ccetti
Lnubicus
Lsenator
Lcollurio
Gcristata
Aarvensis
Cbrachydactyla

Cluster 11

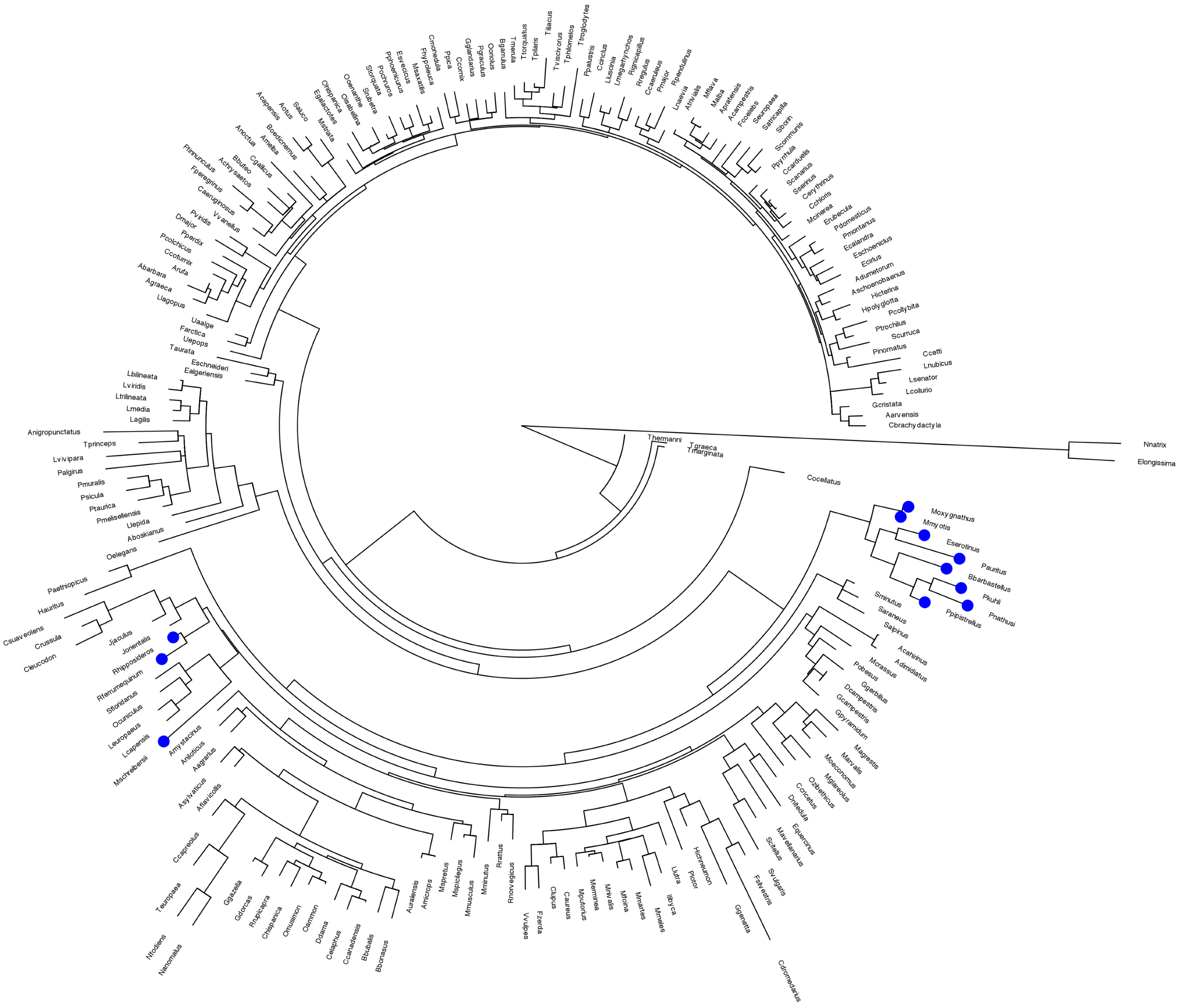

Ttroglodytes
Tphilomelos
Tiliacus
Tviscivorus
Tpilaris
Ttorquatus
Tmerula
Bgarrulus
Ooriolus
Pgraculus
Gglandarius
Ccorax
Ppica
Cmonedula
Phypoleuca
Msaxatilis
Esvecicus
Pphoenicurus
Pochruros
Storquata
Srubetra
Oisabellina
Ooenanthe
Ohispanica
Egalactotes
Mstriata
Saluco
Aotus
Acapensis
Bcedicnemus
Amelba
Anoctua
Cgallicus
Bbuteo
Achrysaetos
Ftinnunculus
Fperegrinus
Caeruginosus
Vvanellus
Pviridis
Dmajor
Pperdix
Pcolchicus
Ccoturnix
Arufa
Abarbara
Agraeca
Llagopus
Uaalge
Farctica
Uepops
Taurata
Eschneideri
Ealgeriensis
Lbilineata
Lviridis
Ltrilineata
Lmedia
Lagilis
Anigropunctatus
Tprinceps
Lvivipara
Palgirus
Pmuralis
Psicula
Ptaurica
Pmelisellensis
Llepida
Aboskianus
Oelegans
Paethiopicus
Hauritus
Csuaveolens
Crussula
Cleucodon
Jjaculus
Jorientalis
Rhipposideros
Rferrumequinum
Sfloridanus
Ocuniculus
Leuropaeus
Lcapensis
Mschreibersii
Amystacinus
Aniloticus
Aagrarius
Asylvaticus
Aflavicollis
Ccapreolus
Teuropaea
Nfodiens
Nanomalus
Ggazella
Gdorcas
Rrupicapra
Chispanica
Omusimon
Oammon
Ddama
Celaphus
Ccanadensis
Bbubalis
Bbonasus
Auralensis
Amicrops
Mspretus
Mspicilegus
Mmusculus
Mminutus
Rrattus
Rnorvegicus
Vvulpes
Fzerda
Clupus
Caureus
Mputorius
Merminea
Mnivalis
Mfoina
Mmartes
Mmeles
Llutra
Flibyca
Hichneumon
Ggenetta
Cdromedarius
Fsilvestris
Svulgaris
Scitellus
Mavellanarius
Equercinus
Dnitedula
Ccricetus
Oziebethicus
Mglareolus
Moeconomus
Marvalis
Magrestis
Gpyramidum
Gcampestris
Dcampestris
Ggerbillus
Pobesus
Mcrassus
Admidiatus
Acahirinus
Salpinus
Saraneus
Sminutus
Ppipistrellus
Pnathusi
Pkuhli
Bbarbastellus
Pauritus
Eserotinus
Mmyotis
Moxygnathus
Cocellatus
Thermanni
Tgraeca
Tmarginata
Nnatrix
Elongissima
Cbrachydactyla
Aarvensis
Gcristata
Lcollurio
Lsenator
Lnubicus
Ccetti
Pinornatus
Scurruca
Ptrochilus
Pcollybita
Hpolyglotta
Hicterina
Aschoenobaenus
Adumetorum
Ecirlus
Eschoeniclus
Ecalandra
Pmontanus
Pdomesticus
Erubecula
Mcinerea
Cchloris
Cerythrinus
Sserinus
Scanarius
Ccarduelis
Ppyrrhula
Scommunis
Sborin
Satricapilla
Seuropaea
Fcoelebs
Acampestris
Apratensis
Malba
Mflava
Atrivialis
Lnaevia
Rpendulinus
Pmajor
Ccaeruleus
Rregulus
Rignicapillus
Lmegarhynchos
Lluscinia
Ccinclus
Ppalustris

Supplementary Figure 4: Dendrograms of the environmental distances for vertebrate species available in the GBIF and involved in the network of ticks, pathogens, and vertebrates in the western Palearctic, overlaid with the species of ticks and pathogens recorded for each species (blue dots). These dendrograms complement the information in Table 2, and are produced separately for each cluster of species detected in the main network. The figures are zoomable without loss of resolution.

Cluster 0

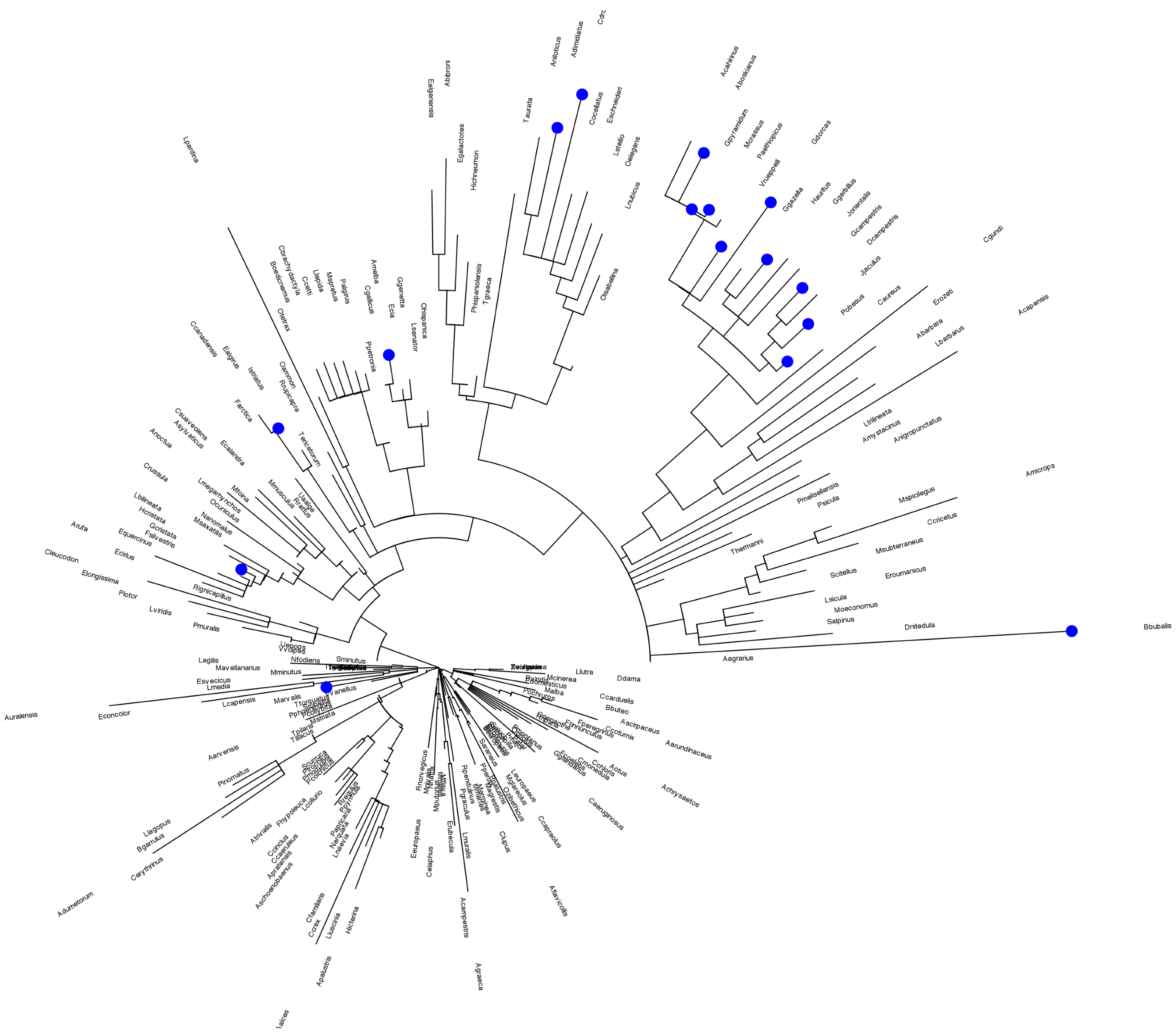

Lpardina
Ealgeriensis
Abibroni
Aniloticus
Adimidiatus
Taurata
Cocellatus
Eschneideri
Acahirinus
Aboskianus
Gpyramidum
Mcrassus
Paethiopicus
Gdorcas
Lstellio
Oelegans
Egalactotes
Hichneumon
Lnubicus
Vrueppelli
Ggazella
Hauritus
Ggerbillus
Jorientalis
Gcampestris
Dcampestris
Cgundi
Jjaculus
Oisabellina
Phispanolensis
Tgraeca
Cbrachydactyla
Boedicnemus
Ccetti
Llepida
Mspretus
Palgirus
Amelba
Cgallicus
Ggenetta
Ecia
Ohispanica
Lsenator
Otetrax
Pobesus
Caureus
Erozeti
Acapensis
Abarbara
Lbarbarus
Ccanadensis
Eatigrus
Istriatus
Oammon
Rrupicapra
Ppetronia
Farctica
Tericetorum
Csuaveolens
Asylvaticus
Anoctua
Ecalandra
Ltrilineata
Amystacinus
Anigropunctatus
Amicrops
Crussula
Lmegarhynchos
Mfoina
Ocuniculus
Mmusculus
Uaalge
Rrattus
Pmelisellensis
Psicula
Mspicilegus
Ccricetus
Lbilineata
Hcristata
Gcristata
Fsilvestris
Nanomalus
Msaxatilis
Arufa
Equercinus
Themarini
Msubterraneus
Cleucodon
Ecirlus
Scitellus
Eroumanicus
Elongissima
Rignicapillus
Lsicula
Moeconomus
Ptotor
Salpinus
Lviridis
Dnitedula
Bbubalis
Pmuralis
Uepops
Vvulpes
Aagrarius
Lagilis
Nfodiens
Sminutus
Mavellanarius
Llutra
Ddama
Esvecicus
Lmedia
Mminutus
Mcinerea
Ldomesticus
Malba
Vvanellus
Auralensis
Econcolor
Lcapensis
Marvalis
Ttorquatus
Mstriata
Ccarduelis
Bbuteo
Ascirpaceus
Aarundinaceus
Aarvensis
Pinomatus
Achrysaetos
Caeruginosus
Llagopus
Bgarrulus
Cerythrinus
Adumetorum
Atrivialis
Phypoleuca
Lcollurio
Ccinclus
Apratensis
Aschoenobaenus
Ccapreolus
Afalvicollis
Eeuropaeus
Cclaphus
Erubecula
Lmuralis
Clupus
Acampestris
Agraeca
Cfamiliaris
Ccrex
Lluscinia
Hicterina
Apalustris
Malces
Leuropaeus
Rnorvegicus

Cluster 1

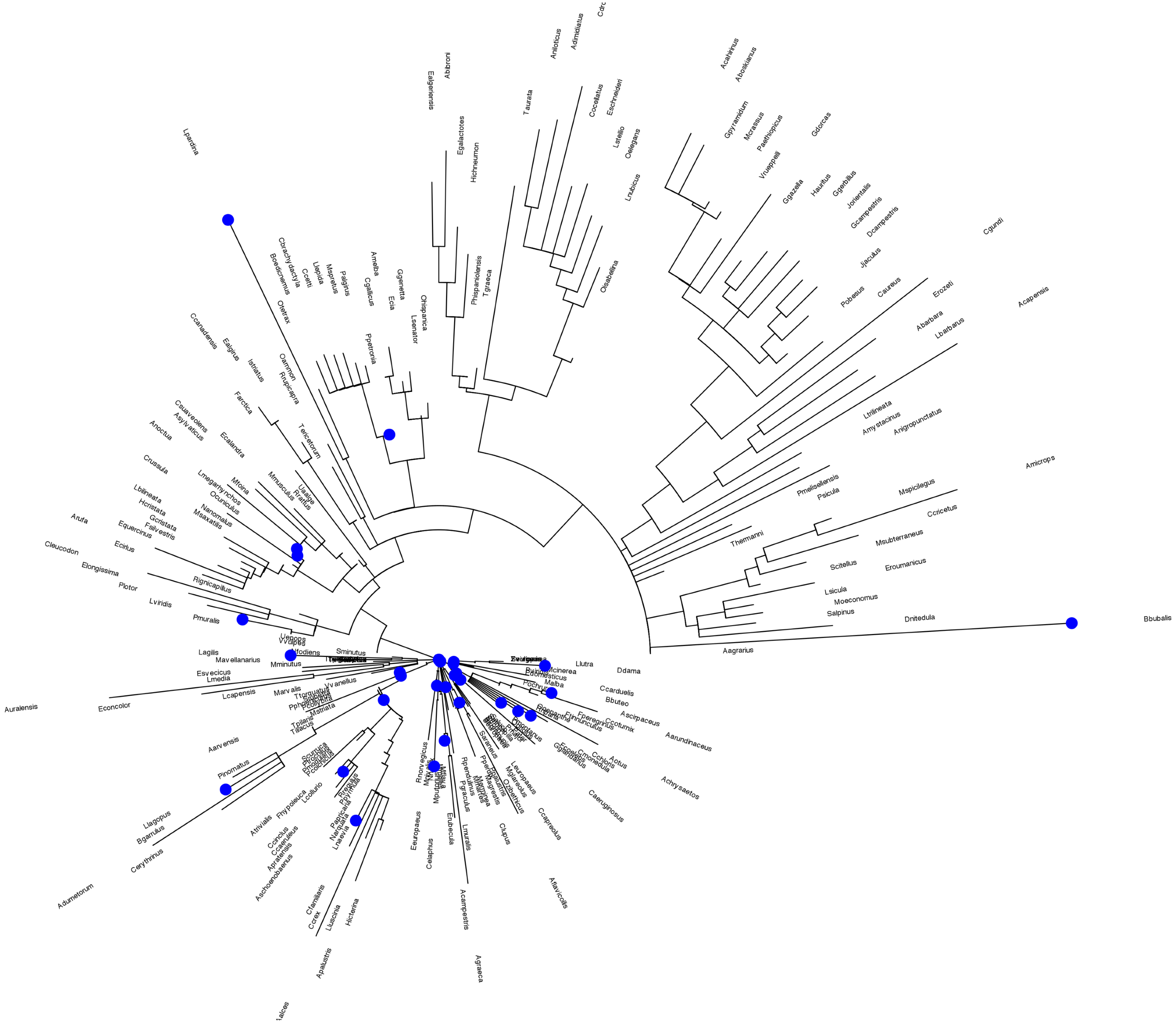

Cluster 2

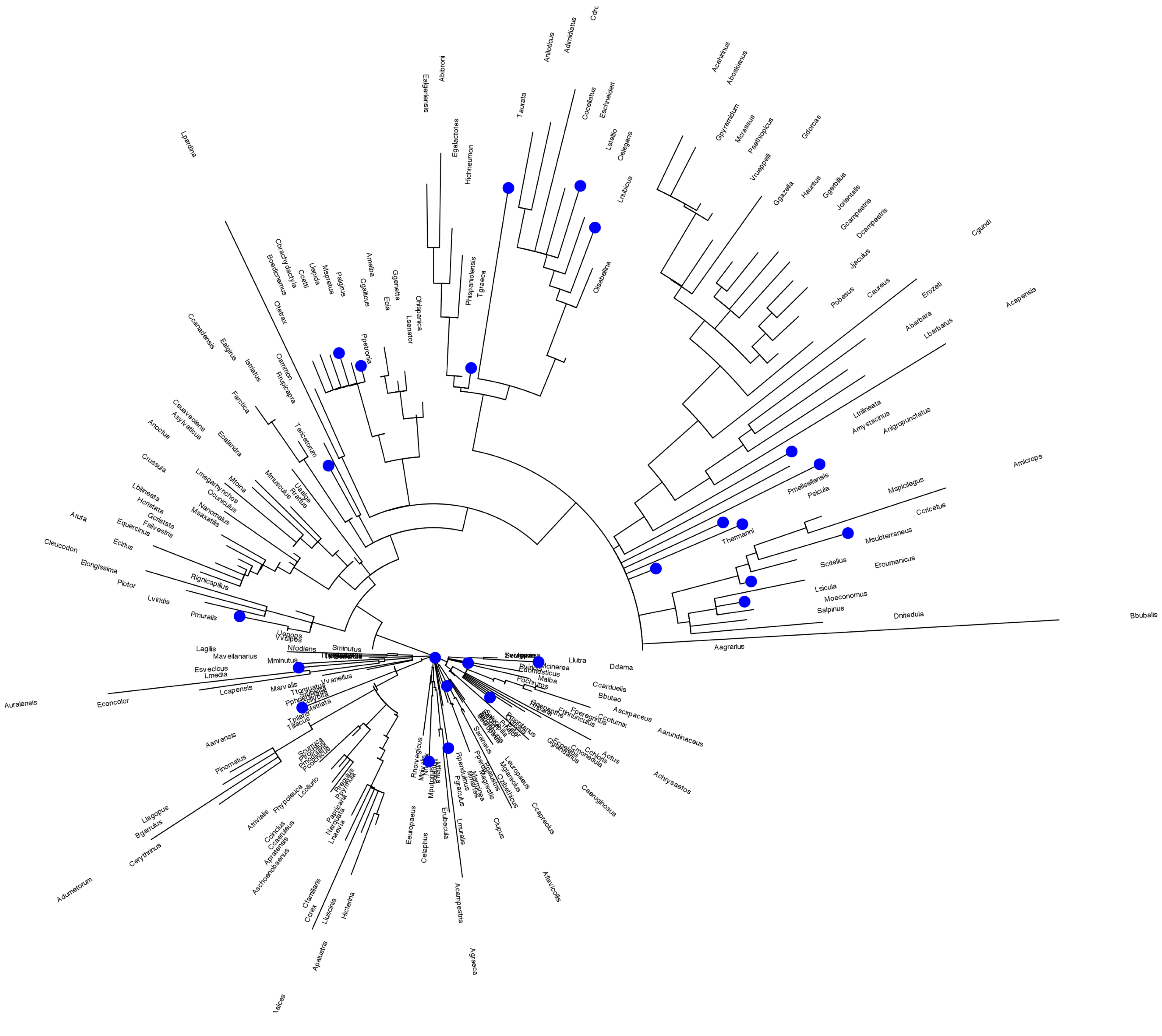

Lpardina
Ealgeriensis
Ablbroni
Aniloticus
Adimidiatus
Taurata
Cocellatus
Eschneideri
Lstellio
Oelegans
Lnubicus
Acahirinus
Aboskianus
Gpyramidum
Mcrassus
Paethiopicus
Gdorcas
Vrueppelli
Ggazella
Hauritus
Ggerbillus
Jorientalis
Gcampestris
Dcampestris
Jjaculus
Gqundi
Pobesus
Caureus
Erozeti
Acapensis
Abarbara
Lbarbarus
Egalactotes
Hichneumon
Phispanioliensis
Tgraeca
Oisabellina
Cbrachydactyla
Boedicnemus
Ccetti
Llepida
Mspretus
Palgirus
Amelba
Cgallicus
Ggenetta
Ecia
Ohispanica
Lsenator
Otetrax
Ppetronia
Ccanadensis
Ealginus
Istriatus
Oammon
Rrupicapra
Farctica
Csuaveolens
Asylvaticus
Anoctua
Ecalandra
Tericetorum
Crussula
Lmegarhynchos
Mfoina
Ocuniculus
Mmusculus
Ualgira
Lbilineata
Hcristata
Gcristata
Fsilvestris
Nanomalus
Msaxatilis
Arufa
Equercinus
Ecirlus
Cleucodon
Elongissima
Rignicapillus
Pplotor
Lviridis
Pmuralis
Vvulpes
Nfodiens
Sminutus
Lagilis
Mavellanarius
Mminutus
Esvecicus
Lmedia
Lcapensis
Marvalis
Vvanellus
Ttorquatus
Tpilaris
Tiliacus
Mstriata
Auralensis
Econcolor
Aarvensis
Pinornatus
Llagopus
Bgarrulus
Cerythrinus
Adumetorum
Atrivialis
Phypoleuca
Lcollurio
Ccinclus
Apratensis
Aschoenobaenus
Cfamiliaris
Ccrex
Lluscinia
Hicterina
Apalustris
Aalces
Rnorvegicus
Europaeus
Celaphus
Erubecula
Lmuralis
Acampestris
Agraeca
Rpendulinus
Pgraculus
Saraneus
Clupus
Ccapreolus
Aflavicollis
Leuropaeus
Mgiareola
Caeruginosus
Achrysaetos
Ldama
Ccarduelis
Bbuteo
Ascirpaceus
Aarundinaceus
Cmonedula
Cchloris
Aotus
Ccoturnix
Pperegrinus
Ftinnunculus
Pdomesticus
Malba
Llutra
Ltrilineata
Amystacinus
Anigropunctatus
Pmelisellensis
Psicula
Thermanni
Amicrops
Mspicilegus
Ccricetus
Msubterraneus
Scitellus
Eroumanicus
Lsicula
Moeconomus
Salpinus
Dnitedula
Bbubalis
Aagrarius

Cluster 3

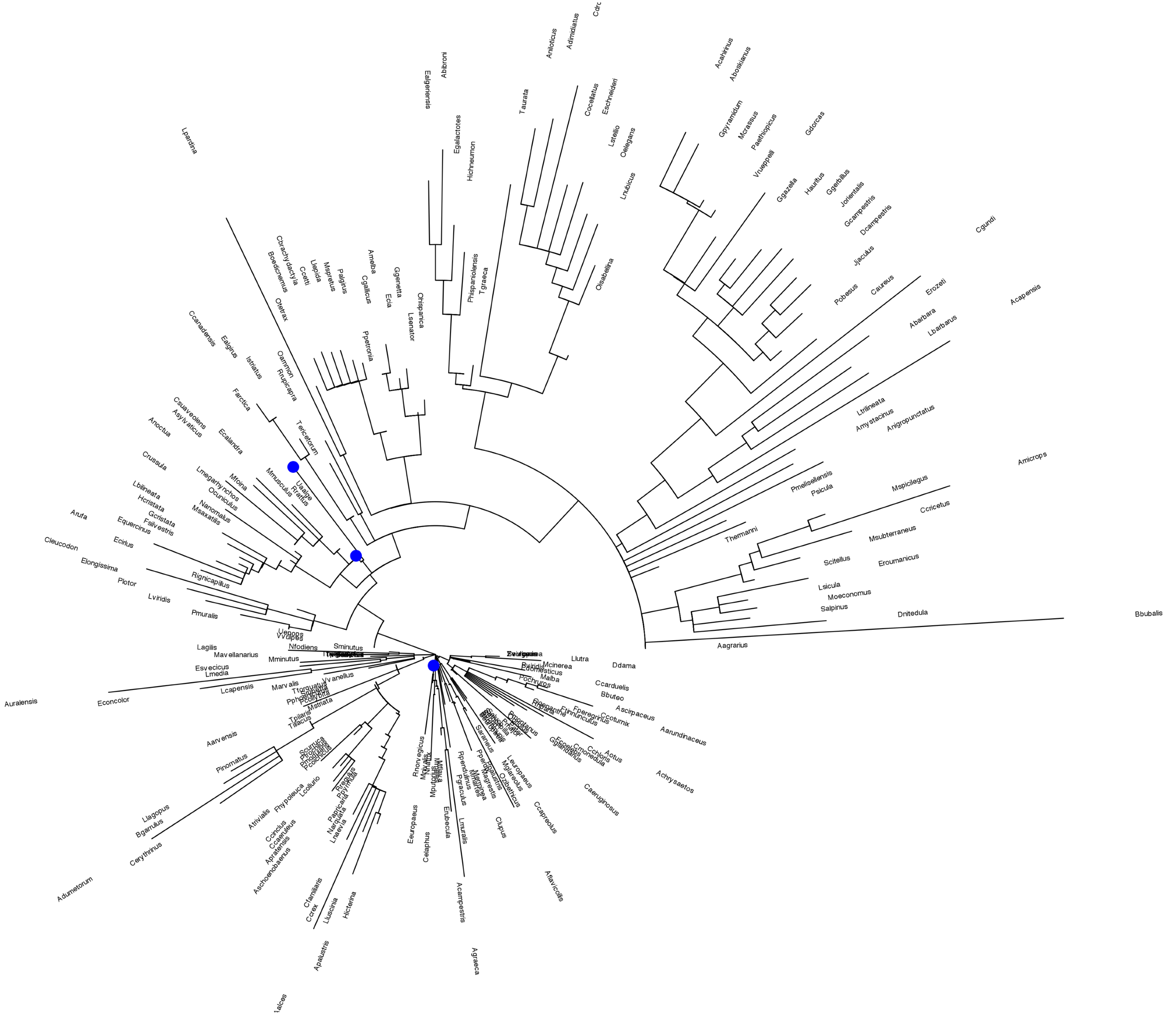

Lpardina
Ealgeriensis
Abibroni
Aniloticus
Adimidiatus
Taurata
Cocellatus
Eschneideri
Lstellio
Oelegans
Lnubicus
Acahirinus
Aboskianus
Gpyramidum
Mcrassus
Paethiopicus
Gdorcas
Vrueppelli
Ggazella
Hauritus
Ggerbillus
Jorientalis
Gcampestris
Dcampestris
Cgundi
Jjaculus
Pobesus
Caureus
Erozeti
Acapensis
Abarbara
Lbarbarus
Ltrilineata
Amystacinus
Anigropunctatus
Amicrops
Pmelisellensis
Psicula
Mspicilegus
Ccricetus
Themanni
Msubterraneus
Scitellus
Eroumanicus
Lsicula
Moeconomus
Salpinus
Dnitedula
Bbubalis
Aagrarius
Egalactotes
Hichneumon
Phispanolensis
Tgraeca
Oisabellina
Cbrachydactyla
Boedicnemus
Coetti
Llepida
Mspretus
Palgirus
Amelba
Cgallicus
Ggenetta
Ecia
Ohispanica
Lsenator
Otetrax
Ppetronia
Ccanadensis
Ealgirus
Istriatus
Oammon
Rrupicapra
Farctica
Tercetorum
Csuaveolens
Asylvaticus
Anoctua
Ecalandra
Crussula
Lmegarhynchos
Mfoina
Mmusculus
Ocuniculus
Nanomalus
Msaxatilis
Lbilineata
Hcristata
Gcristata
Fsilvestris
Arufa
Equercinus
Ecirlus
Cleucodon
Elongissima
Rignicapillus
Plotor
Lviridis
Pmuralis
Vvulpes
Nfodiens
Sminutus
Lagilis
Mavellanarius
Mminutus
Esvecicus
Lmedia
Lcapensis
Econcolor
Auralensis
Marvalis
Vvanellus
Ttorquatus
Mstriata
Tpilaris
Aarvensis
Pinornatus
Llagopus
Bgarrulus
Cerythrinus
Adumetorum
Atrivialis
Fhypoleuca
Lcollurio
Ccinclus
Apratensis
Aschoenobaenus
Cfamiliaris
Ccrex
Lluscinia
Hicterina
Apaludtris
Aalces
Europaeus
Celaphus
Erubecula
Lmuralis
Acampestris
Agraeca
Rnorvegicus
Pgraculus
Clupus
Ccapreolus
Aflavicollis
Llutra
Ddama
Mcinerea
Malba
Ccarduelis
Bbuteo
Ascirpaceus
Aarundinaceus
Cjunco
Achrysaetos
Caeruginosus
Leuropaeus
Saraneus
Fperegrinus
Ftinnunculus
Ccoturnix

Cluster 4

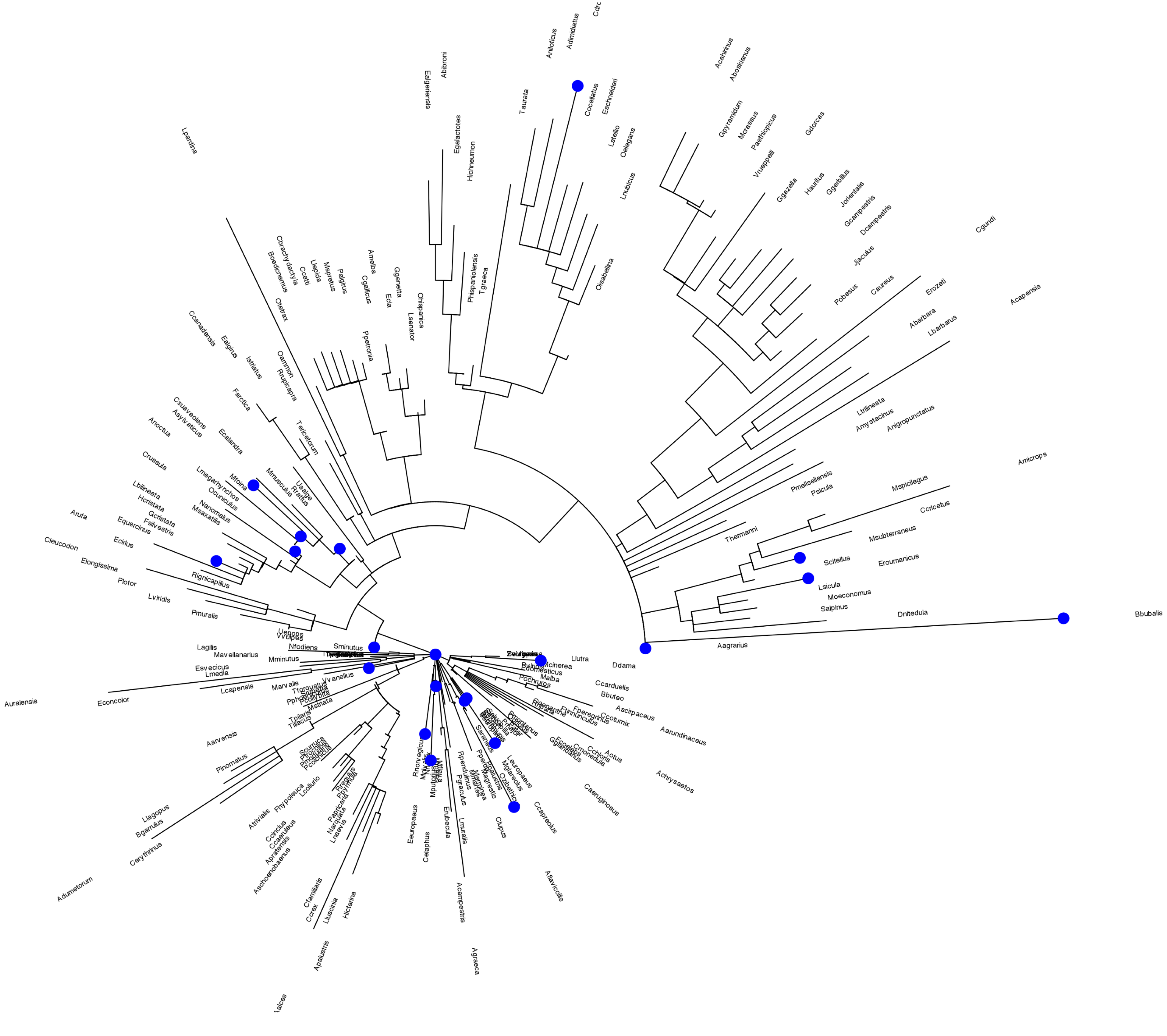

Lpardina
Ealgeriensis
Abibroni
Aniloticus
Adimidiatus
Taurata
Cocellatus
Eschneideri
Lstellio
Oelegans
Lnubicus
Egalactotes
Hichneumon
Phispaniolensis
Tgraeca
Oisabellina
Acahirinus
Aboskianus
Gpyramidum
Mcrassus
Paethiopicus
Gdorcas
Vrueppelli
Ggazella
Hauritus
Ggerbillus
Jorientalis
Gcampestris
Dcampestris
Cgundi
Jjaculus
Pobesus
Caureus
Erozeti
Acapensis
Abarbara
Lbarbarus
Ltrilineata
Amystacinus
Anigropunctatus
Amicrops
Pmelisellensis
Psicula
Mspicilegus
Ccricetus
Thermanni
Msubterraneus
Scitellus
Eroumanicus
Lsicula
Moeconomus
Salpinus
Dnitedula
Bbubalis
Aagrarius
Ddama
Llutra
Ccarduelis
Bbuteo
Ascirpaceus
Aarundinaceus
Achrysaetos
Caeruginosus
Ccapreolus
Aflavicollis
Clupus
Acampestris
Agraeca
Lmuralis
Erubecula
Celaphus
Eeuropaeus
Cbrachydactyla
Boedicnemus
Ccetti
Llepida
Mspretus
Palgirus
Amelba
Cgallicus
Ggenetta
Ecia
Ohispanica
Lsenator
Otetrax
Ppetronia
Ccanadensis
Ealginus
Istriatus
Oammon
Rrupicapra
Farctica
Tercetorum
Csuaveolens
Asylvaticus
Anoctua
Ecalandra
Crussula
Mfoina
Mmusculus
Lmegarhynchos
Ocuniculus
Rrattus
Lbilineata
Hcristata
Gcristata
Fsilvestris
Nanomalus
Msaxatilis
Arufa
Equercinus
Cleucodon
Ecirlus
Elongissima
Ptotor
Rignicapillus
Lviridis
Pmuralis
Uepops
Vvulpes
Nfodiens
Sminutus
Lagilis
Mavellanarius
Mminutus
Esvecicus
Lmedia
Auralensis
Econcolor
Lcapensis
Marvalis
Vvanellus
Ttorquatus
Mstriata
Tpilaris
Tiliacus
Aarvensis
Pinornatus
Llagopus
Bgarrulus
Cerythrinus
Adumetorum
Atrivialis
Fhypoleuca
Lcollurio
Ccinclus
Apratensis
Aschoenobaenus
Cfamiliaris
Ccrex
Lluscinia
Hicterina
Apalustris
Rnorvegicus
Rpendulinus
Pgraculus
Ccoturnix
Fperegrinus
Ftinnunculus
Pochruros
Malba
Edomesticus
Leuropaeus

Cluster 5

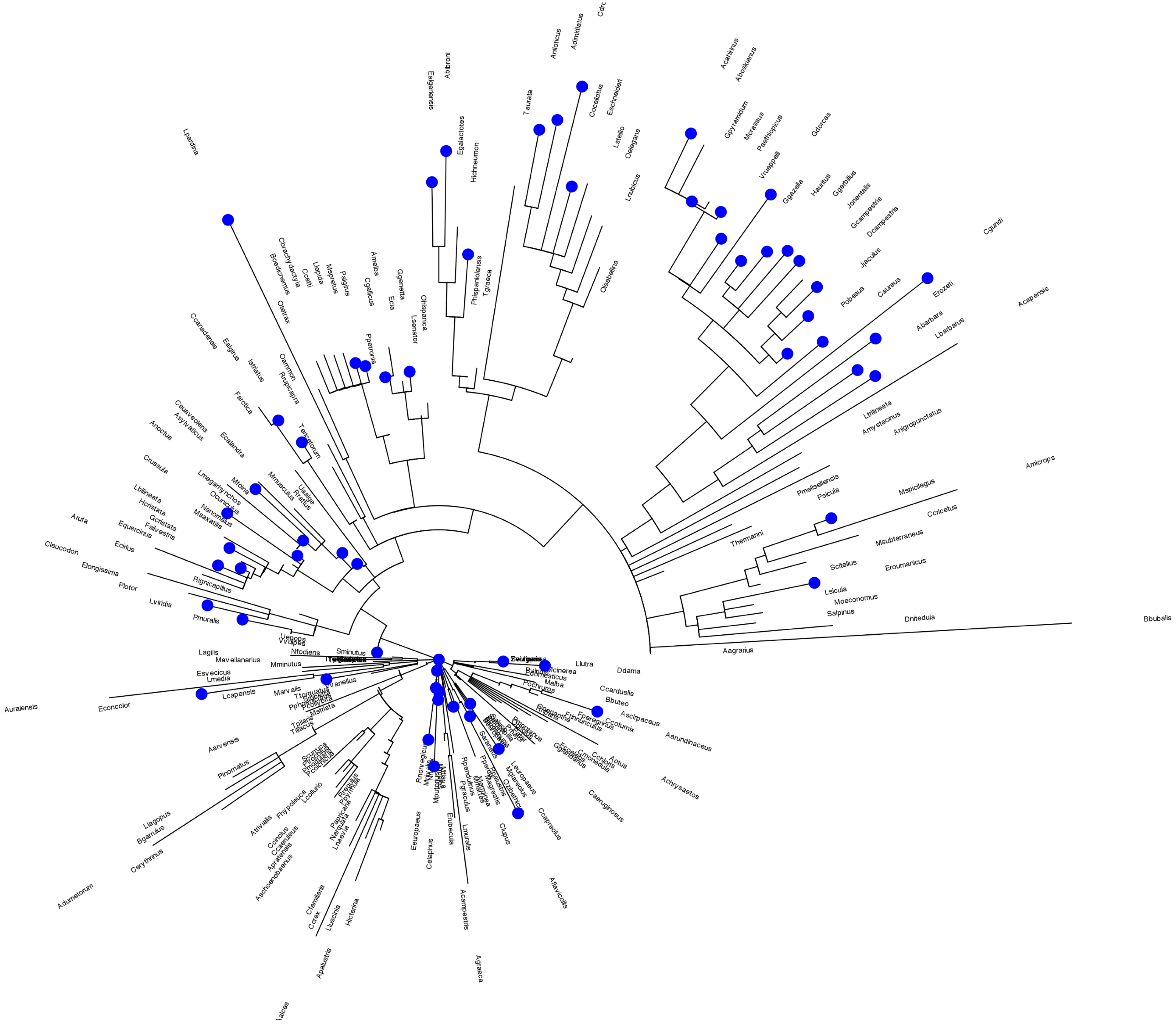

Lpardina
Ealgeriensis
Aalbironi
Egalactotes
Hichneumon
Phispaniolensis
Tgraeca
Aniloticus
Adimidiatus
Taurata
Cocellatus
Eschneideri
Lstellio
Oelegans
Lnubicus
Oolsabellina
Acahirinus
Aboskianus
Gpyramidum
Mcrassus
Paethiopicus
Gdorcas
Vrueppelli
Ggazella
Haurtus
Ggerbillus
Jorientalis
Gcampestris
Dcampestris
Jjaculus
Pobesus
Caureus
Erozeti
Cgundi
Abarbara
Lbarbarus
Acapensis
Ltrilineata
Amystacinus
Anigropunctatus
Amicrops
Pmelisellensis
Psicula
Mspicilegus
Ccricetus
Thermanni
Msubterraneus
Scitellus
Eroumanicus
Lsicula
Moeconomus
Salpinus
Dnitedula
Bbubalis
Aagrarius
Cbrachydactyla
Boedicnemus
Ccetti
Llepida
Mspretus
Palgirus
Ameliba
Cgallicus
Ggenetta
Ecia
Ohispanica
Lsenator
Otetrax
Ppetronia
Ccanadensis
Eatigrus
Istriatus
Oammon
Rrupicapra
Farctica
Tenuirostrum
Csuaveolens
Asylvaticus
Anoctua
Ecalandra
Crussula
Lmegarhynchos
Mfoina
Mmusculus
Ualgirus
Rrattus
Ocuniculus
Lbilineata
Hcristata
Gcristata
Fsilvestris
Nanomalus
Msaxatilis
Arufa
Equercinus
Ecirlus
Cleucodon
Elongissima
Rignicapillus
Plotor
Lviridis
Pmuralis
Uepops
Vvulpes
Nfodiens
Sminutus
Lagilis
Mavellanarius
Mminutus
Esvecicus
Lmedia
Lcapensis
Vvanellus
Auralensis
Econcolor
Marvalis
Ttorquatus
Mstriata
Ttilaria
Aarvensis
Pinominatus
Llagopus
Bgarrulus
Cerythrinus
Adumetorum
Atrivialis
Fhypoleuca
Lcollurio
Ccinclus
Apratensis
Aschoenobaenus
Cfamiliaris
Ccrex
Lluscinia
Hicterina
Apalustris
Aalces
Europaeus
Celaphus
Erubecula
Lmuralis
Acampestris
Agraeca
Llutra
Ddama
Ccarduelis
Bbuteo
Ascirpaceus
Ccoturnix
Aarundinaceus
Achrysaetos
Caeruginosus
Ccapreolus
Lupus
Aflavicollis

Cluster 6

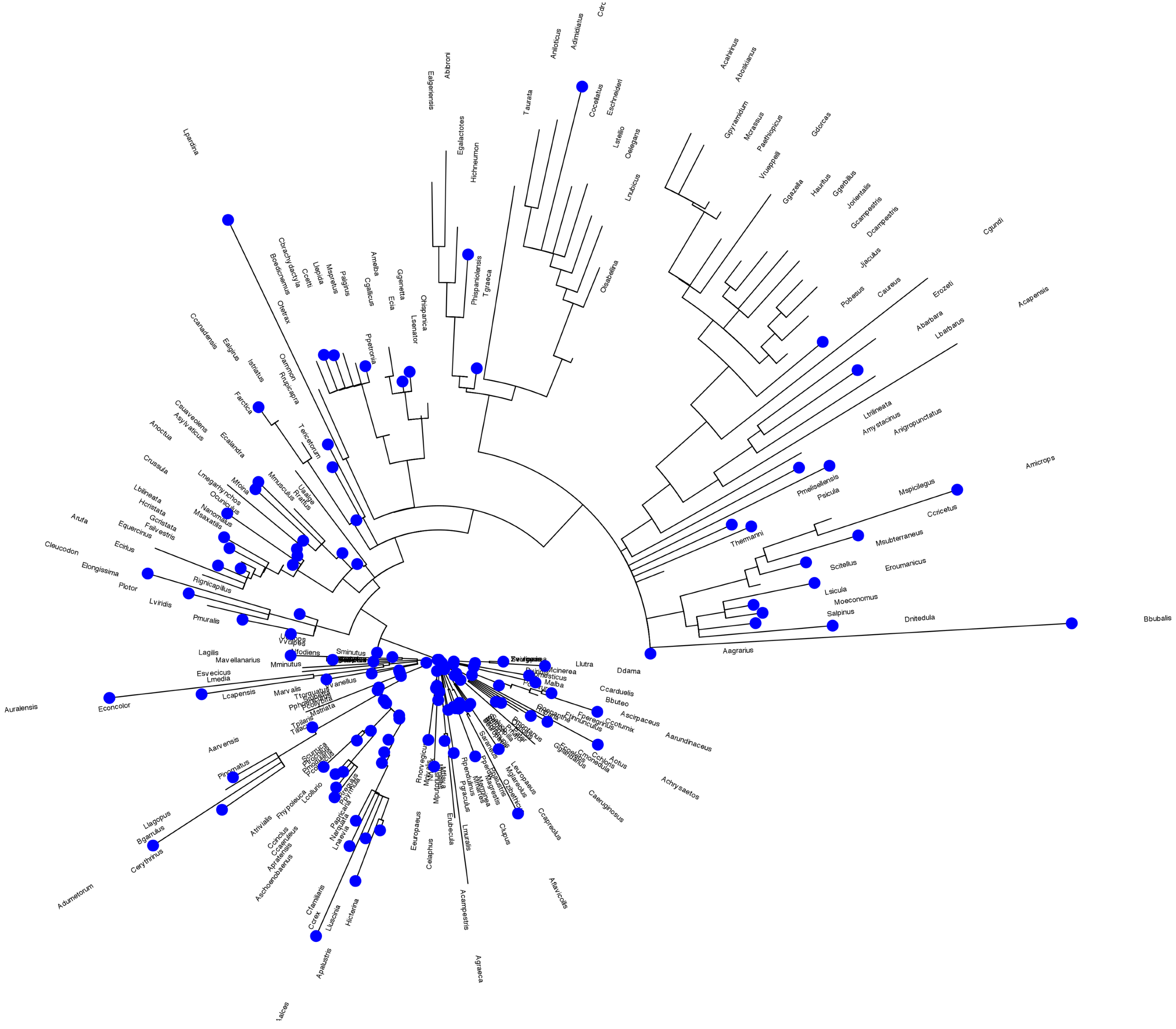

Lpardina
Ealgeriensis
Abibroni
Egalactotes
Hichneumon
Phispanicolensis
Tgraeca
Taurata
Aniloticus
Adimidiatus
Cocellatus
Eschneideri
Lstellio
Oelegans
Lnubicus
Oisabellina
Acahirinus
Aboskianus
Gpyramidum
Mcrassus
Paethiopicus
Gdorcas
Vrueppelli
Ggazella
Hauritus
Ggerbillus
Jorientalis
Gcampestris
Dcampestris
Jjaculus
Cgundi
Pobesus
Caureus
Erozeti
Abarbara
Lbarbarus
Acapensis
Ltrilineata
Amystacinus
Anigropunctatus
Pmeliellensis
Psicula
Amicrops
Mspicilegus
Ccricetus
Themanini
Msubterraneus
Scitellus
Eroumanicus
Lsicula
Moeconomus
Salpinus
Dnitedula
Bbubalis
Aagrarius
Ddama
Llutra
Ccarduelis
Bbuteo
Ascirpaceus
Aarundinaceus
Achrysaetos
Caeruginosus
Ccapreolus
Aflavicollis
Agraeca
Acampestris
Lmuralis
Erubecula
Celaphus
Eeuropaeus
Cfamiliaris
Ccrex
Lluscinia
Hicterina
Apalustris
Adumetorum
Ceythrinus
Llagopus
Bgarrulus
Atrivialis
Phypoleuca
Aarvensis
Auralensis
Econcolor
Lcapensis
Esvecicus
Lmedia
Mminutus
Mavellanarius
Lagilis
Sminutus
Vvulpes
Pmuralis
Lviridis
Ptor
Elongissima
Cleucodon
Ecirlus
Rignicapillus
Arufa
Equercinus
Fsilvestris
Gcristata
Hcristata
Lbilineata
Crussula
Anoctua
Asylvaticus
Csuaveolens
Ccanadensis
Ealgirus
Istriatus
Farctica
Ecalandra
Oammon
Rrupicapra
Tercetorum
Lmegarhynchos
Mfoina
Ocuniculus
Nanomalus
Msaxatilis
Mmusculus
Rrattus
Usalpe
Otetrax
Boedicnemus
Cbrachydactyla
Ccetti
Llepida
Mspretus
Palgirus
Amelba
Cgallicus
Ggenetta
Ecia
Ohispanica
Lsenator
Ppetronia

Cluster 9

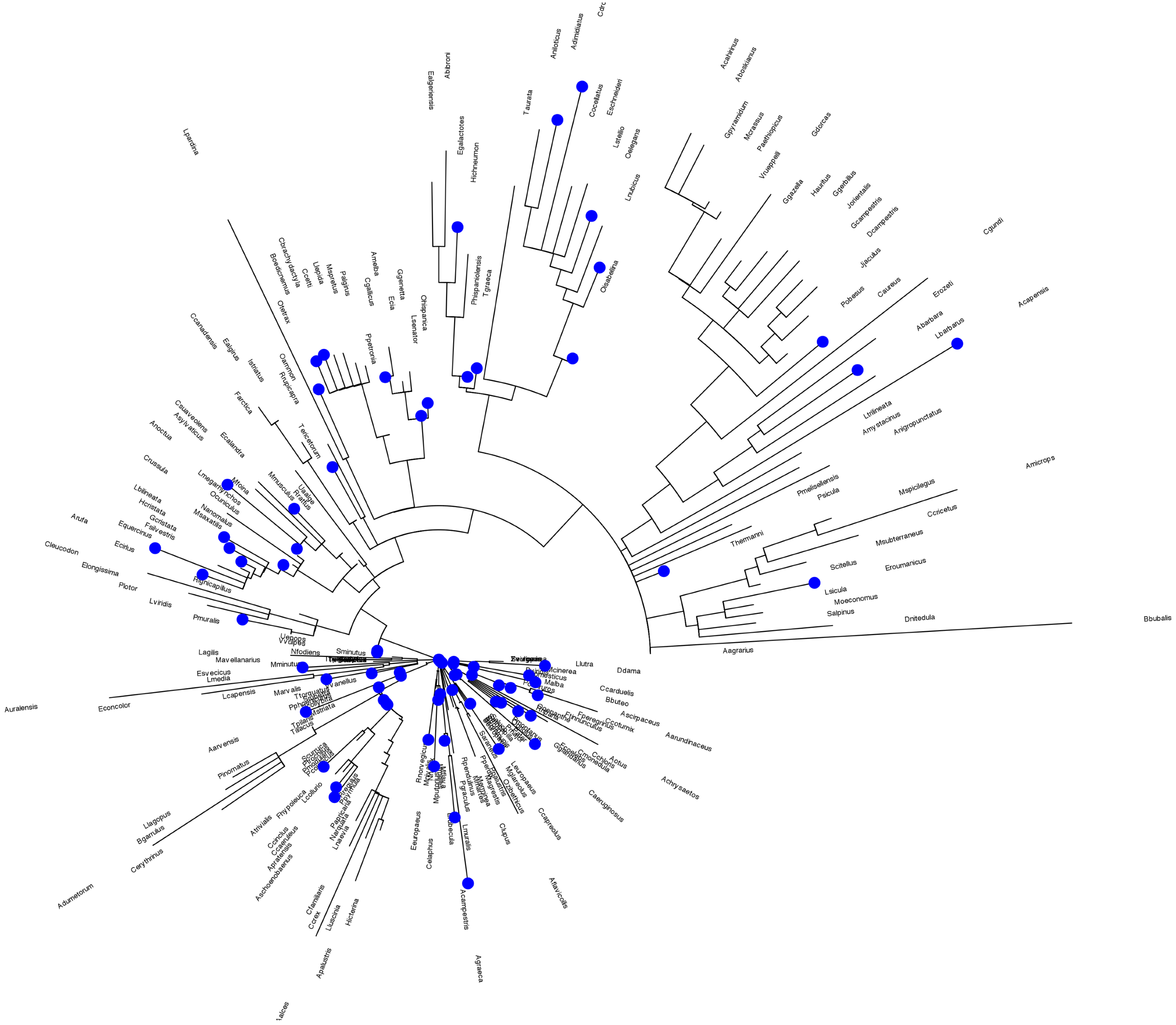
Lpardina
Ealgeriensis
Abiboni
Egalactotes
Hichneumon
Taurata
Aniloticus
Adimidiatus
Cocellatus
Eschneideri
Lstellio
Oelegans
Lnubicus
Acahirinus
Aboskianus
Gpyramidum
Mcrassus
Paethiopicus
Vrueppelli
Gdorcas
Ggazella
Hauritus
Ggerbillus
Jorientalis
Gcampestris
Dcampestris
Jjaculus
Pobesus
Caureus
Erozeti
Abarbara
Lbarbarus
Acapensis
Cgundi
Ltrilineata
Amystacinus
Anigropunctatus
Amicrops
Pmelisellensis
Psicula
Mspicilegus
Ccricetus
Thermanni
Msubterraneus
Scitellus
Eroumanicus
Lsicula
Moeconomus
Salpinus
Dnitedula
Bbubalis
Aagrarius
Ddama
Llutra
Ccarduelis
Bbuteo
Ascirpaceus
Aarundinaceus
Achrysaetos
Caeruginosus
Ccapreolus
Aflavicollis
Agraeca
Acampestris
Lmuralis
Clupus
Eeuropaeus
Celaphus
Apalustris
Hicterina
Lluscinia
Ccrex
Cfamiliaris
Aschoenobaenus
Atrivialis
Adumetorum
Cerythrinus
Bgarrulus
Llagopus
Pinornatus
Aarvensis
Auralensis
Econcolor
Lcapensis
Lmedia
Esvecicus
Mavellanarius
Lagilis
Mminutus
Nfodiens
Sminutus
Vvulpes
Pmuralis
Lviridis
Plotor
Elongissima
Cleucodon
Ecirlus
Arufa
Equercinus
Fsilvestris
Gcristata
Hcristata
Lbilineata
Crussula
Anoctua
Asylvaticus
Csuaveolens
Ecalandra
Farctica
Ccanadensis
Ealgirus
Istriatus
Oammon
Rrupicapra
Tercetorum
Ofetrax
Boedicnemus
Cbrachydactyla
Ccetti
Llepida
Mspretus
Palgirus
Amelba
Cgallicus
Ggenetta
Ecia
Ohispanica
Lsenator
Ppetronia
Phispaniolensis
Tgraeca
Oisabellina
Mmusculus
Rrattus
Lmegarhynchos
Ocuniculus
Nanomalus
Msaxatilis
Rignicapillus
Marvalis
Ttorquatus
Vvanellus
Mstriata
Tpilaris
Tiliacus
Hrupestris
Lcollurio
Ccinclus
Cerulus
Apratensis
Rpendulinus
Pgraculus
Cccoturnix
Fperegrinus
Ctinnunculus
Cchloris
Actus
Emonedula
Ggandarius
Leuropaeus
Malba
Cdomesticus
Mcinerea
Lnatalia
Rnorvegicus

Supplementary Table 1: Complete taxonomic information about the vertebrates included in each cluster of the studied network.

Cluster	Taxon	Genus	Family	Order
0	Aalbiventris	Atelerix	Erinaceidae	Erinaceomorpha
0	Aboskianus	Acanthodactylus	Lacertidae	Squamata
0	Amelba	Apus	Apodidae	Apodiformes
0	Cdromedarius	Camelus	Camelidae	Artiodactyla
0	Ealgirus	Erinaceus	Erinaceidae	Erinaceomorpha
0	Gcalurus	Gerbillus	Muridae	Rodentia
0	Gcampestris	Gerbillus	Muridae	Rodentia
0	Gdorcas	Gazella	Bovidae	Artiodactyla
0	Gpyramidum	Gerbillus	Muridae	Rodentia
0	Hauritus	Hemiechinus	Erinaceidae	Erinaceomorpha
0	Jjaculus	Jaculus	Dipodidae	Rodentia
0	Larabicus	Lepus	Leporidae	Lagomoprha
0	Mcrassus	Meriones	Muridae	Rodentia
1	Bgarrulus	Bombycilla	Bombycillidae	Passerifomes
1	Ccaeruleus	Cyanistes	Paridae	Passeriformes
1	Cfamiliaris	Certhia	Certhidae	Passeriformes
1	Cmonedula	Corvus	Corvidae	Passeriformes
1	Lmegarhynchos	Luscinia	Muscicapidae	Passeriformes
1	Lpardina	Lynx	Felidae	Carnivora
1	Nnatrix	Natrix	Natricidae	Squamata
1	Pdomesticus	Passer	Passeridae	Passeriformes
1	Pmajor	Parus	Paridae	Passeriformes
1	Pmontanus	Passer	Passeridae	Passeriformes
1	Ppetronia	Petronia	Passeridae	Passeriformes
1	Rpendulinus	Remiz	Remizidae	Passeriformes
1	Satricapilla	Sylvia	Sylviidae	Passeriformes
1	Scurruca	Sylvia	Sylviidae	Passeriformes
1	Tviscivorus	Turdus	Turdidae	Passeriformes
2	Aarvensis	Alauda	Alaudidae	Passeriformes
2	Acappadocia	Apathya	Lacertidae	Squamata
2	Anigropunctatus	Algyroides	Lacertidae	Squamata
2	Ccricetus	Cricetus	Cricetidae	Rodentia
2	Chispanica	Capra	Ovidae	Artiodactyla
2	Eschneideri	Eumeces	Scincidae	Squamata
2	Llepida	Lacerta	Lacertidae	Squamata
2	Lmedia	Lacerta	Lacertidae	Squamata
2	Lmuralis	Lacerta	Lacertidae	Squamata
2	Lsicula	Lacerta	Lacertidae	Squamata
2	Ltrilineata	Lacerta	Lacertidae	Squamata
2	Oelegans	Ophisops	Lacertidae	Squamata
2	Palgirus	Psammodromus	Lacertidae	Squamata
2	Pmelisellensis	Podarcis	Lacertidae	Squamata
2	Psicula	Podarcis	Lacertidae	Squamata
2	Qtrachyblepharus	Quedentfeldtia	Gekkonidae	Squamata
2	Scitellus	Spermophilus	Sciuridae	Rodentia

2 Taurata	Trachylepis	Scincidae	Squamata
2 Tnatrix	Tropidonotus	Colubridae	Squamata
2 Tprinceps	Timon	Scincidae	Squamata
2 Zviridiflavus	Zamenis	Colubridae	Squamata
3 Farctica	Fratercula	Alcidae	Charadriiformes
3 Uaalge	Uria	Alcidae	Charadriiformes
4 Bbubalis	Bubalus	Bovidae	Artiodactyla
4 Equercinus	Eliomys	Gliridae	Rodentia
4 Sscrofa barbarus	Sus	Suidae	Artiodactyla
4 Sscrofa ferus	Sus	Suidae	Artiodactyla
5 Aalgirus	Aetechinus	Erinaceidae	Erinaceomorpha
5 Aalqirus	Aethechinus	Erinaceidae	Erinaceomorpha
5 Aarundinaceus	Acrocephalus	Acrocephalidae	Passeriformes
5 Abarbara	Alectoris	Phasianidae	Galliformes
5 Abibroni	Agama	Agamidae	Squamata
5 Acahirinus	Acomys	Muridae	Rodentia
5 Adimidiatus	Acomys	Muridae	Rodentia
5 Aniloticus	Arvicanthis	Muridae	Rodentia
5 Caureus	Canis	Canidae	Carnivora
5 Cgallicus	Circaetus	Accipitridae	Accipitrifomres
5 Cgundi	Ctenodactylus	Ctenodactyilidae	Rodentia
5 Cocellatus	Chalcides	Scincidae	Squamata
5 Crussula	Crocidura	Soricidae	Soricomorpha
5 Dcampestris	Dipodillus	Muridae	Rodentia
5 Ealgeriensis	Eumeces	Scincidae	Squamata
5 Econcolor	Erinaceus	Erinaceidae	Erinaceomorpha
5 Eeuropaeus	Erinaceus	Erinaceidae	Erinaceomorpha
5 Emumbyanus	Eliomys	Gliridae	Rodentia
5 Erozeti	Elephantulus	Macroscelidae	Macroscelidea
5 Flybica	Felis	Felidae	Carnivora
5 Fsilvestris	Felis	Felidae	Carnivora
5 Fzerda	Fennecus	Canidae	Carnivora
5 Ggazella	Gazella	Bovidae	Artiodactyla
5 Ggenetta	Genetta	Viverridae	Carnivora
5 Ggerbillus	Gerbillus	Muridae	Rodentia
5 Hcristata	Hystrix	Hystricidae	Rodentia
5 Hichneumon	Herpestes	Herpestidae	Carnivora
5 Ilibyca	Ictonyx	Mustelidae	Carnivora
5 Ilybica	Ictonyx	Mustelidae	Carnivora
5 Istriatus	Ictonyx	Mustelidae	Carnivora
5 Jorientalis	Jaculus	Dipodidae	Rodentia
5 Lbarbarus	Lemniscomys	Muridae	Rodentia
5 Lbarcaeus	Lepus	Leporidae	Lagomoprha
5 Lcapensis	Lepus	Leporidae	Lagomoprha
5 Llutra	Lutra	Mustelidae	Carnivora
5 Mfoina	Martes	Mustelidae	Carnivora

5	Mmartes	Martes	Mustelidae	Carnivora
5	Mmeles	Meles	Mustelidae	Carnivora
5	Mnumidica	Mustela	Mustelidae	Carnivora
5	Mputorius	Mustela	Mustelidae	Carnivora
5	Mshawii	Meriones	Muridae	Rodentia
5	Mspicilegus	Mus	Muridae	Rodentia
5	Mspretus	Mus	Muridae	Rodentia
5	Ocuniculus	Oryctolagus	Leporidae	Lagomoprha
5	Ozibethicus	Ondatra	Cricetidae	Rodentia
5	Paethiopicus	Paraechinus	Erinaceidae	Erinaceomorpha
5	Pdeserti	Paraechinus	Erinaceidae	Erinaceomorpha
5	Pdorsalis	Paraechinus	Erinaceidae	Erinaceomorpha
5	Plotor	Procyon	Procyonidae	Carnivora
5	Pobesus	Psammomys	Muridae	Rodentia
5	Rrattus	Rattus	Muridae	Rodentia
5	Sfloridanus	Sylvilagus	Leporidae	Lagomoprha
5	Vrueppelli	Vulpes	Canidae	Carnivora
5	Vvulpes	Vulpes	Canidae	Carnivora
6	Aagrarius	Apodemus	Muridae	Rodentia
6	Aalces	Alces	Cervidae	Artiodactyla
6	Achrysaetos	Aquila	Accipitridae	Accipitrifomres
6	Adumetorum	Acrocephalus	Acrocephalidae	Passeriformes
6	Aflavicolis	Apodemus	Muridae	Rodentia
6	Aflavicollis	Apodemus	Muridae	Rodentia
6	Ahypoleucos	Actitis	Scolopacidae	Charadriiformes
6	Amelanopogon	Acrocephalus	Acrocephalidae	Passeriformes
6	Amicrops	Apodemus	Muridae	Rodentia
6	Amystacinus	Apodemus	Muridae	Rodentia
6	Aotus	Asio	Strigidae	Strigiformes
6	Apalustris	Acrocephalus	Acrocephalidae	Passeriformes
6	Aschoenobaenus	Acrocephalus	Acrocephalidae	Passeriformes
6	Ascirpaceus	Acrocephalus	Acrocephalidae	Passeriformes
6	Asherman	Arvicola	Cricetidae	Rodentia
6	Asylvaticus	Apodemus	Muridae	Rodentia
6	Aterrestris	Arvicola	Cricetidae	Rodentia
6	Atrivialis	Anthus	Motacillidae	Passeriformes
6	Auralensis	Apodemus	Muridae	Rodentia
6	Ccanadensis	Cervus	Ovidae	Artiodactyla
6	Ccapreolus	Capreolus	Ovidae	Artiodactyla
6	Ccarduelis	Carduelis	Fringillidae	Passeriformes
6	Ccetti	Cettia	Sylviidae	Passeriformes
6	Ccinclus	Cinclus	Cinclidae	Passeriformes
6	Ccrex	Crex	Rallidae	Gruiformes
6	Celaphus	Cervus	Ovidae	Artiodactyla
6	Cerythrinus	Carpodacus	Certhidae	Passeriformes
6	Cleucodon	Crocidura	Soricidae	Soricomorpha

6	Clupus	Canis	Canidae	Carnivora
6	Cminuta	Calidris	Charadriiformes	Aves
6	Csuaveolens	Crocidura	Soricidae	Soricomorpha
6	Ddama	Dama	Ovidae	Artiodactyla
6	Dnitedula	Dryomys	Gliridae	Rodentia
6	Ecia	Emberiza	Emberizidae	Passeriformes
6	Elongissima	Elaphe	Colubridae	Squamata
6	Eroumanicus	Erinaceus	Erinaceidae	Erinaceomorpha
6	Erubecula	Erithacus	Muscicapidae	Passeriformes
6	Eschoeniclus	Emberiza	Emberizidae	Passeriformes
6	Fcoelebs	Fringilla	Fringillidae	Passeriformes
6	Fhypoleuca	Ficedula	Muscicapidae	Passeriformes
6	Gglis	Glis	Gliridae	Rodentia
6	Hicterina	Hippolais	Acrocephalidae	Passeriformes
6	Hrustica	Hirundo	Hirundinidae	Passeriformes
6	Lagilis	Lacerta	Lacertidae	Squamata
6	Lbilineata	Lacerta	Lacertidae	Squamata
6	Lcollurio	Lanius	Laniidae	Passeriformes
6	Ldugesii	Lacerta	Lacertidae	Squamata
6	Llagopus	Lagopus	Phasianidae	Galliformes
6	Lluscinia	Luscinia	Muscicapidae	Passeriformes
6	Llusciniοides	Locustella	Locustellidae	Passeriformes
6	Lnaevia	Locustella	Locustellidae	Passeriformes
6	Lridibundus	Larus	Laridae	Charadriiformes
6	Lviridis	Lacerta	Lacertidae	Squamata
6	Lvivipara	Lacerta	Lacertidae	Squamata
6	Magrestis	Microtus	Cricetidae	Rodentia
6	Marvalis	Microtus	Cricetidae	Rodentia
6	Mavellanarius	Muscardinus	Gliridae	Rodentia
6	Mcinerea	Motacilla	Motacillidae	Passeriformes
6	Merminea	Mustela	Mustelidae	Carnivora
6	Mglareolus	Myodes	Cricetidae	Rodentia
6	Mminutus	Micromys	Muridae	Rodentia
6	Mmusculus	Mus	Muridae	Rodentia
6	Mnivalis	Mustela	Mustelidae	Carnivora
6	Moeconomus	Microtus	Cricetidae	Rodentia
6	Mstriata	Muscicapa	Muscicapidae	Passeriformes
6	Msubterraneus	Microtus	Cricetidae	Rodentia
6	Nanomalus	Neomys	Soricidae	Soricomorpha
6	Narquata	Numenius	Scolopacidae	Charadriiformes
6	Nfodiens	Neomys	Soricidae	Soricomorpha
6	Oammon	Ovis	Ovidae	Artiodactyla
6	Omusimon	Ovis	Ovidae	Artiodactyla
6	Papricaria	Pluvialis	Charadriidae	Charadriiformes
6	Pater	Parus	Paridae	Passeriformes
6	Pcollybita	Phylloscopus	Phylloscopidae	Passeriformes

6 Perolius	Phylloscopus	Phylloscopidae	Passeriformes
6 Pgraculus	Pyrrhocorax	Corvidae	Passerifomes
6 Pinornatus	Phylloscopus	Phylloscopidae	Passeriformes
6 Pmodularis	Prunella	Prunellidae	Passeriformes
6 Pmuralis	Podarcis	Lacertidae	Squamata
6 Pochruros	Phoenicurus	Muscicapidae	Passeriformes
6 Ppalustris	Parus	Paridae	Passeriformes
6 Pphoenicurus	Phoenicurus	Muscicapidae	Passeriformes
6 Ppugnax	Philomachus	Scolopacidae	Charadriiformes
6 Ppyrrhula	Pyrrhula	Fringillidae	Passeriformes
6 Psubterraneus	Pitymys	Cricetidae	Rodentia
6 Ptaurica	Podarcis	Lacertidae	Squamata
6 Ptrochilus	Phylloscopus	Phylloscopidae	Passeriformes
6 Pviridis	Picus	Picidae	Piciformes
6 Rignicapillus	Regulus	Corvidae	Passeriformes
6 Rnorvegicus	Rattus	Muridae	Rodentia
6 Rregulus	Regulus	Corvidae	Passeriformes
6 Rrupicapra	Rupicapra	Bovidae	Artiodactyla
6 Salpinus	Sorex	Soricidae	Soricomorpha
6 Saraneus	Sorex	Soricidae	Soricomorpha
6 Sborin	Sylvia	Sylviidae	Passeriformes
6 Scommunis	Sylvia	Sylviidae	Passeriformes
6 Scoronatus	Sorex	Soricidae	Soricomorpha
6 Seuropaea	Sitta	Sittidae	Passeriformes
6 Sminutus	Sorex	Soricidae	Soricomorpha
6 Srubetra	Saxicola	Muscicapidae	Passeriformes
6 Sserinus	Serinus	Fringillidae	Passeriformes
6 Svulgaris	Sciurus	Sciuridae	Rodentia
6 Sxanthoprymnus	Spermophilus	Sciuridae	Rodentia
6 Talba guttata	Tyto	Tytonidae	Strigiformes
6 Tericetorum	Turdus	Turdidae	Passeriformes
6 Terythropus	Tringa	Scolopacidae	Charadriiformes
6 Teuropaea	Talpa	Talpidae	Soricomorpha
6 Tiliacus	Turdus	Turdidae	Passeriformes
6 Tmerula	Turdus	Turdidae	Passeriformes
6 Tphilomelos	Turdus	Turdidae	Passeriformes
6 Tpilaris	Turdus	Turdidae	Passeriformes
6 Ttorquatus	Turdus	Turdidae	Passeriformes
6 Ttotanus	Tringa	Scolopacidae	Charadriiformes
6 Ttroglodytes	Troglodytes	Trogloditidae	Passeriformes
6 Vvanellus	Vanellus	Charadriidae	Charadriiformes
6 Zvivipara	Zootoca	Lacertidae	Squamata
7 Bibis	Bubulcus	Ardeidae	Pelecaniformes
8 Clivia	Columba	Columbidae	Columbiformes
9 Aarboreus	Anthus	Motacillidae	Passeriformes
9 Acampestris	Anthus	Motacillidae	Passeriformes

9	Acapensis	Asio	Strigidae	Strigiformes
9	Agraeca	Alectoris	Phasianidae	Galliformes
9	Anoctua	Athene	Strigidae	Strigiformes
9	Apratensis	Anthus	Motacillidae	Passeriformes
9	Arufa	Alectoris	Phasianidae	Galliformes
9	Bbonasus	Bison	Bovidae	Artiodactyla
9	Bbuteo	Buteo	Accipitridae	Accipitrifomres
9	Boedicnemus	Burhinus	Charadriiformes	Aves
9	Caeruginosus	Circus	Accipitridae	Accipitrifomres
9	Cbrachydactyla	Calandrella	Alaudidae	Passeriformes
9	Cchloris	Carduelis	Fringillidae	Passeriformes
9	Ccoccathraustes	Coccothraustes	Fringillidae	Passeriformes
9	Ccornix	Corvus	Corvidae	Passeriformes
9	Ccoturnix	Coturnix	Phasianidae	Galliformes
9	Dmajor	Dryobates	Picidae	Piciformes
9	Ecalandra	Emberiza	Emberizidae	Passeriformes
9	Ecirlus	Emberiza	Emberizidae	Passeriformes
9	Egalactotes	Erythropygia	Muscicapidae	Passeriformes
9	Esvecicus	Erithacus	Muscicapidae	Passeriformes
9	Fperegrinus	Falco	Falconidae	Falconiformes
9	Ftinnunculus	Falco	Falconidae	Falconiformes
9	Gcristata	Galerida	Alaudidae	Passeriformes
9	Gglandarius	Garrulus	Corvidae	Passeriformes
9	Hpolyglotta	Hypolais	Acrocephalidae	Passeriformes
9	Leuropaeus	Lepus	Leporidae	Lagomoprha
9	Lnubicus	Lanius	Laniidae	Passeriformes
9	Lsenator	Lanius	Laniidae	Passeriformes
9	Lstellio	Laudakia	Agamidae	Squamata
9	Malba	Motacilla	Muscicapidae	Passeriformes
9	Mflava	Motacilla	Muscicapidae	Passeriformes
9	Msaxatilis	Monticola	Muscicapidae	Passeriformes
9	Ohispanica	Oenanthe	Muscicapidae	Passeriformes
9	Oisabellina	Oenanthe	Muscicapidae	Passeriformes
9	Ooenanthe	Oenanthe	Muscicapidae	Passeriformes
9	Ooriolus	Oriolus	Oriolidae	Passeriformes
9	Otetrax	Otis	Otididae	Gruiformes
9	Pcinerea	Perdix	Phasianidae	Galliformes
9	Pcolchicus	Phasianus	Phasianidae	Galliformes
9	Phispaniolensis	Passer	Passeridae	Passeriformes
9	Pperdix	Perdix	Phasianidae	Galliformes
9	Ppica	Pica	Corvidae	Passeriformes
9	Prubra	Perdix	Phasianidae	Galliformes
9	Saluco	Strix	Sciuridae	Rodentia
9	Scanarius	Serinus	Fringillidae	Passeriformes
9	Storquata	Saxicola	Muscicapidae	Passeriformes
9	Tgraeca	Testudo	Testudinidae	Testudines

9	Thermanni	Testudo	Testudinidae	Testudines
9	Tmarginata	Testudo	Testudinidae	Testudines
9	Tmauritanica	Testudo	Testudinidae	Testudines
9	Uepops	Upupa	Upupidae	Coraciiformes
11	Bbarbastellus	Barbastella	Vespertilionidae	Chiroptera
11	Eserotinus	Eptesicus	Vespertilionidae	Chiroptera
11	Mbechsteinii	Myotis	Vespertilionidae	Chiroptera
11	Mdaubentonii	Myotis	Vespertilionidae	Chiroptera
11	Mmyotis	Myotis	Vespertilionidae	Chiroptera
11	Moxygnathus	Myotis	Vespertilionidae	Chiroptera
11	Mschreibersii	Miniopterus	Miniopteridae	Chiroptera
11	Pauritus	Plecotus	Vespertilionidae	Chiroptera
11	Pkuhli	Pipistrellus	Vespertilionidae	Chiroptera
11	Pnathusi	Pipistrellus	Vespertilionidae	Chiroptera
11	Ppipistrellus	Pipistrellus	Vespertilionidae	Chiroptera
11	Rferrumequinum	Rhinolophus	Rhinolophidae	Chiroptera
11	Rhipposideros	Rhinolophus	Rhinolophidae	Chiroptera
12	Rriparia	Riparia	Hirundinidae	Passeriformes

Supplementary Table 2: Taxonomic list of vertebrates used to calculate the distance matrix based on cytochrome b sequences. GenBank identifiers are also included.

#,Species and sequence
1,A.agrarius.AB303226
2,A.arvensis.JX236372
3,A.barbara.Z48771
4,A.cahirinus.NC_020758
5,A.campestris.U46771
6,A.capensis.EU348960
7,A.cappadocica.GQ142127
8,A.chrysaetos.NC_024087
9,A.dimidiatus.Z96062
10,A.dumetorum.AJ004773
11,A.flavicollis.AB032853
12,A.graeca.Z48772
13,A.hypoleucos.AY894229
14,A.melanopogon.AJ004282
15,A.melba.AY526108
16,A.microps.AF159393
17,A.mystacinus.AJ311146
18,A.nigropunctatus.GQ142132
19,A.niloticus.KF478426
20,A.noctua.AJ003948
21,A.otus.AF082067
22,A.sinensis.EU016090
23,A.pratensis.U46774
24,A.rufa.HG940431
25,A.scherman.JX457751
26,A.schoenobaenus.Z73475
27,A.sylvaticus.AB033695
28,A.terrestris.AF159400
29,A.trivialis.U46775
30,A.uralensis.AB096837
31,B.barbastellus.JQ683212
32,B.bonasus.AY689186
33,B.bubalis.D88637
34,B.buteo.NC_003128
35,B.garrulus.AF285796
36,B.ibis.AF193823
37,C.aeruginosus.AY987305
38,C.aureus.AY291433
39,C.dukhunensis.KF060417
40,C.cornix.HE805701
41,C.nelsoni.AY347753
42,C.caeruleus.AF347961
43,C.capreolus.KJ558333
44,C.carduelis.AY495383
45,C.cetti.JX236380
46,C.chloris.AY495384
47,C.cinclus.AM502167
48,C.coccothraustes.DQ792780
49,C.coturnix.L08377
50,C.cricetus.AJ490310
51,C.dromedarius.U06426
52,C.elaphus.AB001612
53,C.erythrinus.KJ456212

54,C.familiaris.JX236379
55,C.galactotes.KJ173613
56,C.gallicus.AY987253
57,C.leucodon.EF417545
58,C.livia.KC811464
59,C.minuta.KC969163
60,C.monedula.KJ456237
61,C.hispanica.EU081041
62,C.russula.NC_006893
63,C.suaveolens.EU742613
64,D.campestris.KF496218
65,D.dama.AJ000022
66,D.major.DQ479259
67,D.nitedula.AJ225116
68,E.calandra.EF529949
69,E.cirlus.AF284081
70,E.quercinus.AJ225030
71,E.rozeti.KF742641
72,E.rubecula.AY491533
73,E.schoeniclus.EF529944
74,E.serotinus.AF376837
75,F.arctica.DQ385228
76,F.coelebs.AF447368
77,F.hypoleuca.HM633303
78,F.peregrinus.U83307
79,F.silvestris.EF689046
80,F.tinnunculus.EU233131
81,G.campestris.AJ851271
82,G.cristata.AY769746
83,G.dorcas.KC188752
84,G.gazella.KC188776
85,G.genetta.AF511054
86,G.gerbillus.KF496219
87,G.glandarius.AB242559
88,G.glis.NC001892
89,G.pyramidum.KF496283
90,H.auritus.HQ857522
91,H.ichneumon.AF511059
92,H.icterina.AJ004796
93,H.polyglotta.AJ004797
94,H.r.transitiva.JN642423
95,I.libyca.EF987739
96,J.jaculus.JX885206
97,J.orientalis.JN652664
98,L.agilis.GQ142118
99,L.collurio.EU167000
100,L.dugesii.GQ142121
101,L.europaeus.HQ596474
102,L.scotica.EF571187
103,L.luscinia.HM633318
104,L.luscinioides.HQ706148
105,L.lutra.AF057124
106,L.media.U88603
107,L.megarhynchos.HM633319

108,L.naevia.HQ706147
109,L.nubicus.GU253560
110,L.ridibundus.FM209923
111,L.senator.EF635036
112,L.sinensis.AJ279421
113,L.svecica.HM633323
114,L.galatiensis.KC897021
115,L.viridis.EU116514
116,L.vivipara.U69834
117,M.agrestis.GQ352470
118,M.alba.EU167005
119,M.arvalis.GU187386
120,M.bechsteini.AF376843
121,M.cinerea.AF447370
122,M.crassus.AJ851267
123,M.daubentoni.AF376847
124,M.erminea.EF689077
125,M.foina.HQ386888
126,M.glareolus.HQ288418
127,M.martes.HQ386892
128,M.meles.EF689066
129,M.minutus.FJ827494
130,M.musculus.HM222709
131,M.myotis.AF376860
132,M.nivalis.AF457461
133,M.oeconomus.AB372207
134,M.putorius.EF987746
135,M.saxatilis.EF434531
136,M.schreibersii.AY208139
137,M.spicilegus.AB125775
138,M.spretus.AB033700
139,M.striata.EF081348
140,M.subterraneus.AJ717745
141,N.anomalus.DQ991055
142,N.fodiens.AB175071
143,N.natrix.HF680011
144,O.musimon.FR873152
145,O.ammon.AJ867276
146,O.cuniculus.HQ596486
147,O.elegans.FJ416172
148,O.hispanica.GU055474
149,O.isabellina.GU055475
150,O.oenanthe.GU055483
151,O.oriolus.JQ864511
152,O.zibethicus.KC563206
153,P.aethiopicus.HQ857538
154,P.algirus.DQ150367
155,P.auritus.AB085734
156,P.colchicus.AY368060
157,P.collybita.HQ608821
158,P.domesticus.AY495393
159,P.graculus.JQ864522
160,P.inornatus.DQ792800
161,P.kuhli.AJ504445

162,P.lotor.GU175439
163,P.major.KJ456375
164,P.melisellensis.AY185097
165,P.montanus.AY030118
166,P.muralis.JX065628
167,P.nathusii.AJ504446
168,P.obesus.AY934540
169,P.ochruros.HM633365
170,P.pyrrhula.HQ284620
171,P.palustris.DQ792789
172,P.perdix.GU214276
173,P.phoenicurus.HM633364
174,P.pica.JQ393982
175,P.pipistrellus.KF874521
176,P.pugnax.KC969173
177,P.sicula_a
178,P.sicula_Y
179,P.trochilus.AJ004326
180,P.v.viridis.KF765970
181,R.ferrumequinum.AB085731
182,R.hipposideros.DQ297586
183,R.ignicapillus.AY894888
184,R.norvegicus.HM222710
185,R.tatrica.AB050506
186,R.rattus.AB033702
187,R.regulus.AJ004328
188,S.alpinus.AB175120
189,S.aluco.AJ004045
190,S.araneus.DQ417733
191,S.atricapilla.AM889140
192,S.borin.AJ534549
193,S.canaria.AY914136
194,S.citellus.AM691640
195,S.communis.AJ534538
196,S.coronatus.AJ000419
197,S.curruca.AJ534536
198,S.europaea.DQ792801
199,S.floridanus.AY292724
200,S.minutus.AB175133
201,S.rubetra.GU237097
202,S.serinus.L76263
203,S.torquata.KJ456456
204,S.vulgaris.JQ395053
205,S.xanthoprymnus.AF157909
206,T.guttata.EU349003
207,T.erythropus.AY894226
208,T.europaea.AB037601
209,T.graeca.HF954156
210,T.h.hermanni.AJ888364
211,T.iliacus.EU154624
212,T.lepidus.GQ142119
213,T.m.intermedius.EU154638
214,T.marginata.AJ888310
215,T.philomelos.AY495411

216,T.pilaris.EU154656
217,T.torquatus.DQ910996
218,T.totanus.AY894237
219,T.troglodytes.KJ456501
220,T.viscivorus.DQ910997
221,U.aalge.DQ485892
222,U.epops.EU167030
223,V.vanellus.JQ342156
224,V.vulpes.JX013647
226,V.zerda.KJ603240
,Z.vivipara.GQ142120

Supplementary Table 3: Newick tree of genetic distances calculated for vertebrates in Supplementary Table 2.

((((((((((((((((Cfamiliaris:0.124,((Cbrachydactyla:0.07192,Aarvensis:0.05935)0.
89:0.03575,Gcristata:0.0392)0.51:0.01586)0.57:0.0204,(Lcollurio:0.04562,(Lsen
ator:0.03009,Lnubicus:0.1013)0.47:0.03423)1:0.1791)0:0,((((Ccetti:0.2279,Pino
rnatus:0.0164)0.88:0.05929,(Scurruca:0.1344,(Ptrochilus:0.03561,Pcollybita:0.1
083)0.79:0.03913)0:0.03591)0.77:0.01483,((Hpolyglotta:0.01999,Hicterina:0.07
903)0.95:0.05655,((Aschoenobaenus:0.01685,Amelanopogon:0.08569)0.84:0.02
138,Adumetorum:0.07406)0.84:0.02228)0.76:0.01645)0.76:0.00717,(((((Ecirlus
:0.09682,Eschoeniclus:0.08209)0.4:0.00761,Ecalandra:0.06817)0.75:0.0148,(Pm
ontanus:0.04308,Pdomesticus:0.02993)0.94:0.06153)0.49:0.01593,Erubecula:0.
08366)0.46:0.02033,(Mcinerea:0.0372,(((Cchloris:0.04313,(Cerythrinus:0.0380
4,((Sserinus:0.00622,Scanarius:0.01055)0.58:0.00894,Ccarduelis:0.03938)0.5:0.
00595)0.56:0.00966)0.79:0.01188,Ppyrrhula:0.04877)0.43:0.01233,(((((Scomm
unis:0.03278,Sborin:0.09548)0.75:0.02552,Satricapilla:0.09081)0.93:0.04975,(S
europaea:0.1187,Ccoccothraustes:0.03138)0.67:0.01872)0.18:0.01508,Fcoelebs:
0.1027)0:0,((Acampestris:0.05302,(Apratensis:0.05029,(Malba:0.00568,Mflava:
0.02304)0.96:0.04995)0.56:0.00678)0.86:0.02162,Atrivialis:0.01783)0.91:0.024
43)0.77:0.00644)0.81:0.01196)0.73:0.01152)0.85:0.01722)0.77:0.01093)0.75:0.
00599,(((Lnaevia:0.02084,Apalustri:0.09014)0.49:0.01081,Lluscinioides:0.0739
3)0.91:0.02668,((Rpendulinus:0.1049,Pmajor:0.05478)0.65:0.02058,Ccaeruleus:
0.04368)0.88:0.02671)0.91:0.02393)0.85:0.014,(((Rregulus:0.05742,Rignicapill
us:0.09286)0.93:0.04933,(Lmegarhynchos:0.02698,Lluscinia:0.02607)0.97:0.05
469)0.65:0.02433,(Ccinclus:0.1104,Ppalustris:0.07443)0.78:0.04056)0:0)0.23:0.
01267,((Ttroglodytes:0.2108,((Tphilomelos:0.09191,Tviscivorus:0.05686)0:0.00
515,(((Tiliacus:0.128,Tpilaris:0.01557)0.72:0.02049,Ttorquatus:0.03958)0.8:0.0
3117,Tmerula:0.05699)0.86:0.03072)0.11:0.02972)0.69:0.02052,Bgarrulus:0.11
15)0.77:0.02336)0.76:0.0091,(((Ooriolus:0.08407,Pgraculus:0.05618)0.47:0.022
19,(Gglandarius:0.08116,Ccornix:0.06839)0:0.00481)0:0.00026,(Ppica:0.1067,C
monedula:0.1086)0.55:0.0292)0.95:0.03837)0.69:0.00599,(((Fhypoleuca:0.094
1,(((Msaxatilis:0.03055,(Hrustica:0.1812,Esvecicus:0.05024)0.74:0.04213)0.81:
0.0302,(Pphoenicurus:0.05198,Pochruros:0.05485)0.94:0.04133)0:0.00123,((St
orquata:0.08782,Srubetra:0.02194)0.85:0.02617,((Ooenanthe:0.07079,Oisabelli
na:0.00466)0:0,Ohispanica:0.09323)0.73:0.03067)0.74:0.0065)0.7:0.00555)0.88
:0.02094,Egalactotes:0.1257)0:0.00387,Mstriata:0.0707)0.74:0.01086)0.99:0.04
922,(Lridibundus:0.08305,(((Ttotanus:0.06239,Terythropus:0.03981)0.92:0.045
09,(Ppugnax:0.09822,Cminuta:0.06537)0.83:0.03533)0.86:0.02339,((((Clivia:0.1
137,(Saluco:0.09815,(Aotus:0.04297,Acapensis:0.0737)0.93:0.1023)0.95:0.0963
1)0.74:0.0346,(Boedicnemus:0.1009,Amelba:0.1685)0.45:0.02578)0:0,(((Ahypol
eucos:0.1436,Anoctua:0.2185)0.8:0.04166,((Cgallicus:0.05034,(Bbuteo:0.1265,A
chrysaetos:0.08707)0:0.00142)0.72:0.00947,((Ftinnunculus:0.1181,Fperegrinus
:0.03984)0.98:0.1242,Caeruginosus:0.06026)0.42:0.01651)0.94:0.05313)0.75:0.
02028,Vvanellus:0.08866)0.68:0.00839)0.84:0.0217,((Bibis:0.07075,(Pviridis:0.
09003,Dmajor:0.136)0.97:0.1206)0.72:0.0115,((Pperdix:0.1178,(Pcolchicus:0.1
219,(Ccoturnix:0.0617,((Arufa:0,Abarbara:0.08311)0.14:0.01698,Agraeca:0.056
1)0.91:0.04174)0.81:0.03128)0.49:0.02601)0.043:0.01887,Llagopus:0.09515)0.
95:0.0677)0.83:0.01886)0:0.00065)0.78:0.01013)0.57:0.00697)0.72:0.01879,(U
aalge:0.05663,Farctica:0.07977)0.68:0.017)0.58:0.06109,Uepops:0.1349)0.46:0.
06715,Talbaguttata:0.1702)0.92:0.1286,((((Taurata:0.2299,Eschneideri:0.07284
)0.61:0.03444,Ealgeriensis:0.1243)0.42:0.03798,(((((((Lbilineata:0.01059,Lvirid
is:0.05664)0.95:0.08126,((Ltrilineata:0.0496,Lmedia:0.0323)0.9:0.04109,Lagilis:

0.07205)0.83:0.03895)0.79:0.03134,(((Anigropunctatus:0.3216,Tprinceps:0.170
4)0.74:0.02619,(Zvivipara:0.3207,Palgirus:0.3283)0:0.01908)0:0,(((Pmuralis:0.
09089,Psicula:0.1037)0.82:0.053,Ptaurica:0.1139)0.11:0.0266,Pmelisellensis:0.0
409)0.93:0.08867)0.32:0.09547)0.75:0.02268,(Llepida:0.1925,Acappadocica:0.1
93)0.74:0.026)0.88:0.08943,Aboskianus:0.2582)0.68:0.06279,(Oelegans:0.3661,
Ldugesii:0.07107)0.8:0.09144)0.91:0.09856,(((((Paethiopicus:0.08342,Hauritus:
0.2163)0.99:0.283,((((((Csuaveolens:0.1856,(Crussula:0.06813,Cleucodon:0.126
9)0.65:0.06153)0.98:0.2188,Jjaculus:0.06584)0.2:0.04784,Jorientalis:0.05545)0.
96:0.09899,(Rhipposideros:0.03883,Rferrumequinum:0.1304)0.98:0.1303)0.63:
0.02315,((Sfloridanus:0.1228,(Ocuniculus:0.1046,(Leuropaeus:0.07276,Lcapensi
s:0.07208)0.91:0.08752)0.054:0.03478)0.91:0.1274,Mschreibersii:0.3908)0.86:
0.1027)0:0.00557,(((((Amystacinus:0.1245,((Aniloticus:0.1082,Aagrarius:0.093
26)0.76:0.03015,((((Asylvaticus:0.08872,Aflavicollis:0.06451)0:0.01529,((Ccapr
eolus:0.08777,(Teuropaea:0.1131,(Nfodiens:0.1474,Nanomalus:0.1432)0.93:0.1
847)0.97:0.1959)0.29:0.04668,((((Ggazella:0.01699,Gdorcas:0.05185)0.99:0.12
3,((Rrupicapra:0.07215,Chispanica:0.09071)0.14:0.02006,(Omusimon:0.04077,
Oammon:0.0077)0.78:0.02924)0.87:0.04432)0.85:0.0237,(Ddama:0.07635,(Cela
phus:0.06177,Ccanadensis:0.00619)0.82:0.03272)0.95:0.07846)0.75:0.01068,(B
bubalis:0.1139,Bbonasus:0.1232)0.84:0.03988)0.52:0.02269)0.99:0.1694)0.72:0
.03957,(Auralensis:0.02009,Amicrops:0.00309)0.93:0.09045)0.91:0.07886,(Msp
retus:0.07519,(Mspicilegus:0.01103,Mmusculus:0.09291)0.89:0.04471)0.62:0.0
1187)0.77:0.02681)0.77:0.02478)0.92:0.06109,(Mminutus:0.1401,(Rrattus:0.02
94,Rnorvegicus:0.107)0.76:0.01929)0.87:0.04565)0.72:0.01083,((((Vvulpes:0.1
007,Fzerda:0.09575)0.92:0.0927,(Clupus:0.0269,Caureus:0.05445)0.64:0.05874
)0.84:0.05665,(((Mputorius:0.05585,(Merminea:0.00827,Mnivalis:0.03844)0.44:
0.01309)0.77:0.01848,((Mfoina:0.02643,Mmartes:0.04706)0.96:0.0835,(Mmeles
:0.1331,Ilibyca:0.09297)0.74:0.08512)0:0.00157)0.73:0.03163,Llutra:0.1026)0.
65:0.05873)0.9:0.06873,(Plotor:0.2205,(Hichneumon:0.07833,(Ggenetta:0.2722,
(Cdromedarius:0.4394,Fsilvestris:0.06217)0.87:0.1309)0.043:0.1056)0.64:0.055
1)0.71:0.05169)0.92:0.08108)0.64:0.00833,(((Svulgaris:0.2211,(Sxanthoprymn
us:0.1299,Scitellus:0)0.83:0.09704)0.94:0.1408,(Mavellanarius:0.1388,Gglis:0.1
922)0.83:0.08889)0.66:0.05798,(Equercinus:0.2366,Dnitedula:0.1234)0.82:0.07
278)0.66:0.08108)0.72:0.01141,(Ccricetus:0.2282,(((Ozibethicus:0.09421,Mglar
eolus:0.1282)0.88:0.06535,(Aterrestris:0.01709,Ascherman:0.03782)0.97:0.102
1)0.16:0.02444,(((Moeconomus:0.04957,Marvalis:0.09339)0:0.02269,Magrestis:
0.1325)0.52:0.04156,(Msubterr_a:0.00712,Msubterr_e:0.02802)0.91:0.05784)0.
77:0.04177)0.9:0.05738)0.64:0.03975)0.75:0.0201)0.73:0.0395)0.43:0.05212,((
(((Gpyramidum:0.0924,(Gcampestris:0,Dcampestris:0)0.94:0.0518)0:0.00616,G
gerbillus:0.1082)0.84:0.05538,Pobesus:0.08575)0.76:0.02469,Mcrassus:0.1572)
0.86:0.05098,(Adimidiatus:0.0239,Acahirinus:0.00509)1:0.2794)0.76:0.03048)0
.85:0.04656,((Salpinus:0.1319,(Scoronatus:0.02426,Saraneus:0.00488)0.95:0.07
24)0:0,Sminutus:0.03545)0.95:0.127)0.78:0.0448,((((Ppipistrellus:0.07018,(Pna
thusi:0.1803,Pkuhli:0.127)0.14:0.06622)0.83:0.1117,Bbarbastellus:0.2172)0.62:
0.1105,(Pauritus:0.2905,Eserotinus:0.1161)0.34:0.07737)0.51:0.04848,((Mdaub
entoni:0.0484,Mbechsteini:0.1101)0.85:0.07448,(Mmyotis:0,Moxygnathus:0.022
87)0.81:0.05559)0.58:0.06745)0.92:0.1788)1:0.2911)0.77:0.0445)0.74:0.04708,
Cocellatus:0.1515)0.75:0.03118)0.99:0.3764,Tmarginata:0.0276)0.76:0.02828,T
graeca:0.0647)0.68:0.1205,Thermanni:0)1:0.4468,(Elongissima:0.1996,Nnatrix:
0.2235)1:2.371);

Supplementary Table 4: Taxonomic list of vertebrates used to calculate the distance matrix based on overlapping environmental niches. The list includes all species of vertebrates present in the network for which reliable data on distribution exist in the GBIF (>50 records for each species).

#,Species
1,A.agrarius
2,A.alces
3,A.arundinaceus
4,A.arvensis
5,A.barbara
6,A.bibroni
7,A.boskianus
8,A.cahirinus
9,A.campestris
10,A.capensis
11,A.chrysaetos
12,A.dimidiatus
13,A.dumetorum
14,A.flavicollis
15,A.graeca
16,A.hypoleucos
17,A.melanopogon
18,A.melba
19,A.microps
20,A.mystacinus
21,A.nigropunctatus
22,A.niloticus
23,A.noctua
24,A.otus
25,A.palustris
26,A.pratensis
27,A.rufa
28,A.schoenobaenus
29,A.scirpaceus
30,A.stellio
31,A.sylvaticus
32,A.terrestris
33,A.trivialis
34,A.uralensis
35,B.bonasus
36,B.bubalis
37,B.buteo
38,B.garrulus
39,B.ibis
40,B.oedicnemus
41,C.aeruginosus
42,C.aureus
43,C.brachydactyla
44,C.caeruleus
45,C.canadensis
46,C.capreolus
47,C.carduelis
48,C.cetti
49,C.chloris
50,C.cinclus
51,C.coturnix
52,C.crex
53,C.cricetus

54,C.dromedarius
55,C.dromedarius_Linnaeus
56,C.elaphus
57,C.erythrinus
58,C.familiaris
59,C.gallicus
60,C.gundi
61,C.leucodon
62,C.livia
63,C.lupus
64,C.minuta
65,C.monedula
66,C.ocellatus
67,C.russula
68,C.suaveolens
69,D.campestris
70,D.dama
71,D.nitedula
72,E.algeriensis
73,E.algirus
74,E.calandra
75,E.cia
76,E.cirlus
77,E.concolor
78,E.europaeus
79,E.galactotes
80,E.longissima
81,E.quercinus
82,E.roumanicus
83,E.rozeti
84,E.rubecula
85,E.schneideri
86,E.svecicus
87,F.arctica
88,F.coelebs
89,F.hypoleuca
90,F.libyca
91,F.peregrinus
92,F.silvestris
93,F.speculigera
94,F.tinnunculus
95,G.campestris
96,G.cristata
97,G.dorcas
98,G.gazella
99,G.genetta
100,G.gerbillus
101,G.glandarius
102,G.glis
103,G.pyramidum
104,H.auritus
105,H.cristata
106,H.ichneumon
107,H.icterina

108,I.striatus
109,J.jaculus
110,J.orientalis
111,L.agilis
112,L.barbarus
113,L.bilineata
114,L.capensis
115,L.collurio
116,L.europaeus
117,L.lagopus
118,L.lepida
119,L.luscinia
120,L.luscinioides
121,L.lutra
122,L.media
123,L.megarhynchos
124,L.muralis
125,L.naevia
126,L.nubicus
127,L.ocellata
128,L.pardina
129,L.senator
130,L.sicula
131,L.stellio
132,L.trilineata
133,L.viridis
134,M.agrestis
135,M.alba
136,M.arvalis
137,M.avellanarius
138,M.cinerea
139,M.crassus
140,M.erminea
141,M.flava
142,M.foina
143,M.glareolus
144,M.martes
145,M.meles
146,M.minutus
147,M.musculus
148,M.nivalis
149,M.oeconomus
150,M.putorius
151,M.saxatilis
152,M.spicilegus
153,M.spretus
154,M.striata
155,M.subterraneus
156,N.anomalus
157,N.arquata
158,N.fodiens
159,N.natrix
160,O.ammon
161,O.cuniculus

162,O.elegans
163,O.hispanica
164,O.isabellina
165,O.oenanthe
166,O.oriolus
167,O.tetrax
168,O.zibethicus
169,P.aethiopicus
170,P.algirus
171,P.apricaria
172,P.ater
173,P.colchicus
174,P.collybita
175,P.domesticus
176,P.graculus
177,P.hispaniolensis
178,P.inornatus
179,P.lotor
180,P.major
181,P.melisellensis
182,P.modularis
183,P.montanus
184,P.muralis
185,P.obesus
186,P.ochruros
187,P.palustris
188,P.perdix
189,P.petronia
190,P.phoenicurus
191,P.pugnax
192,P.pyrrhula
193,P.sicula
194,P.subterraneus
195,P.trochilus
196,P.viridis
197,R.ignicapillus
198,R.norvegicus
199,R.pendulinus
200,R.rattus
201,R.regulus
202,R.riparia
203,R.rupicapra
204,S.alpinus
205,S.aluco
206,S.araneus
207,S.atricapilla
208,S.borin
209,S.citellus
210,S.communis
211,S.coronatus
212,S.curruca
213,S.europaea
214,S.minutus
215,S.rubetra

216,S.scrofa
217,S.serinus
218,S.vulgaris
219,S.xanthoprymnus
220,T.alba
221,T.aurata
222,T.ericetorum
223,T.erythropus
224,T.europaea
225,T.graeca
226,T.hermanni
227,T.iliacus
228,T.marginata
229,T.merula
230,T.philomelos
231,T.pilaris
232,T.tetrax
233,T.torquatus
234,T.totanus
235,T.troglodytes
236,T.viscivorus
237,U.aalge
238,U.epops
239,V.rueppelli
240,V.vanellus
241,V.vulpes
242,Z.vivipara

Supplementary Table 5: Newick tree of environmental distances calculated for vertebrates in Supplementary Table 4.

(Zvivipara:0.0000,(((((((((((Aagrarius:0.0000,Bbubalis:0.5850):0.0000,(((Dnite
dula:0.1854,Salpinus:0.0796):0.0000,(Moeconomus:0.0515,(Lsicula:0.0408,(Bbo
0.000sus:0.3077,Eroumanicus:0.0984):0.0310):0.0000):0.0396):0.0165,(Scitellu
s:0.0159,(Msubterraneus:0.0662,(Ccricetus:0.1307,(Amicrops:0.2031,Mspicilegu
s:0.0253):0.0774):0.0151):0.0187):0.0958):0.0524):0.0000,(Thermanni:0.0417,(
Psicula:0.1774,(Pmelisellensis:0.1537,(Anigropunctatus:0.3110,(Amystacinus:0.
2623,(Ltrilineata:0.2240,((Acapensis:0.3802,(Lbarbarus:0.1956,(Abarbara:0.157
5,Erozeti:0.2032):0.0223):0.0646):0.0583,(Flibyca:0.3393,(Cgundi:0.3623,(Caur
eus:0.1188,(((Pobesus:0.0203,(Jjaculus:0.0499,(Dcampestris:0.0599,Gcampestri
s:0.0526):0.0266):0.0268):0.0176,(Jorientalis:0.1060,(Ggerbillus:0.1058,(Haurit
us:0.0694,(Astellio:0.1128,Ggazella:0.0297):0.0074):0.0174):0.0000):0.0341):0.
0000,(Gdorcas:0.1681,(Vrueppelli:0.0778,(Paethiopicus:0.0117,(Mcrassus:0.011
2,(Gpyramidum:0.0000,(Aboskianus:0.0766,Acahirinus:0.0832):0.0000):0.0000)
:0.0038):0.0970):0.0008):0.0190):0.0330):0.0751):0.0758):0.0182):0.0485):0.0
509):0.0100):0.0000):0.0000):0.0000):0.0000):0.0000,(((Oisabellina:0.0063,(Ln
ubicus:0.0594,(Oelegans:0.0979,(Lstellio:0.0839,(Eschneideri:0.1058,(Cocellatus
:0.0963,(Cdromedarius:0.2339,(Adimidiatus:0.1255,Aniloticus:0.1072):0.0555):
0.0000):0.0000):0.0183):0.0224):0.0162):0.0774):0.0567,(Sxanthoprymnus:0.0
791,Taurata:0.2128):0.0580):0.0206,(Tgraeca:0.0288,(Phispaniolensis:0.0000,(
Hichneumon:0.0876,(Fspeculigera:0.0634,(Egalactotes:0.0772,(Abibroni:0.1323,
Ealgeriensis:0.0891):0.0499):0.0120):0.0353):0.0816):0.0154):0.0053):0.0799):
0.0799,(((Ttetrax:0.0000,(Ohispanica:0.0208,(Lsenator:0.0037,(Ggenetta:0.0272
,(Ecia:0.0149,(Amelba:0.0403,Cgallicus:0.0000):0.0245):0.0000):0.0388):0.0000
):0.0553):0.0000,(Ppetronia:0.0000,(Palgirus:0.0230,(Mspretus:0.0296,(Llepida:
0.0470,(Ccetti:0.0475,(Cbrachydactyla:0.0527,(Boedicnemus:0.0160,(Amelanop
ogon:0.0808,Bibis:0.0000):0.0344):0.0322):0.0000):0.0009):0.0006):0.0009):0.
0771):0.0395):0.0658,(Otetrax:0.1027,(Locellata:0.3093,Lpardina:0.2373):0.131
3):0.0932):0.0006):0.0335,(Oammon:0.1127,Rrupicapra:0.0818):0.0431):0.000
0,(Tmargi0.000ta:0.0008,(Tericetorum:0.0000,(Istriatus:0.0255,(Ealgirus:0.000
0,(Ccanadensis:0.0114,Cdromedarius_Lin0.000eus:0.0000):0.0194):0.0687):0.10
58):0.0191):0.0256):0.0357,(Farctica:0.1629,Uaalge:0.0111):0.0216):0.0000,((R
rattus:0.0000,(Mmusculus:0.0000,(Ecalandra:0.0616,(Csuaveolens:0.1229,(Asyl
vaticus:0.0974,(Mfoina:0.0006,(Anoctua:0.1306,Lmegarhynchos:0.0000):0.0000
):0.0000):0.0215):0.0000):0.0288):0.0249):0.0000,(Talba:0.0000,((Ocuniculus:0
.0111,(Crussula:0.1244,Nanomalus:0.0093):0.0000):0.0000,(Msaxatilis:0.0000,(
Lbilineata:0.0713,(Hcristata:0.0564,(Gcristata:0.0218,(Fsilvestris:0.0136,(Equer
cinus:0.0440,(Arufa:0.1337,Ecirlus:0.0593):0.0000):0.0000):0.0050):0.0113):0.0
011):0.0191):0.0201):0.0577):0.0000):0.0301):0.0151,((Gglis:0.1377,Rignicapill
us:0.0266):0.0000,((Scoro0.000tus:0.0000,((Cleucodon:0.1696,Psubterraneus:0.
0400):0.0075,(Elongissima:0.1150,Plotor:0.0857):0.0000):0.0207):0.0460,(Lviri
dis:0.0696,Pmuralis:0.0000):0.0312):0.0005):0.0472):0.0430,(Uepops:0.0000,V
vulpes:0.0000):0.0000):0.0861,(Sminutus:0.0000,((Nfodiens:0.0136,(Lagilis:0.1
398,Mavellanarius:0.0816):0.0146):0.0210,(Mminutus:0.0607,((Esvecicus:0.120
3,(Lmedia:0.1126,(Auralensis:0.2845,Econcolor:0.1571):0.0977):0.0085):0.0388
,(Lcapensis:0.1281,Marvalis:0.0650):0.0000):0.0000):0.0000):0.0283):0.0016):0
.0000,((Vvanellus:0.0000,(Ttotanus:0.0000,((Ttorquatus:0.0000,(Srubetra:0.000
0,(Pphoenicurus:0.0000,(Pcollybita:0.0000,(Aarvensis:0.1409,Mstriata:0.0000):
0.0000):0.0000):0.0000):0.0000):0.0000,(Tpilaris:0.0000,(Tiliacus:0.0000,((Ter
ythropus:0.0000,(Ppug0.000x:0.0000,(Pinornatus:0.0000,(Llagopus:0.0686,(Bga

rrulus:0.0861,(Adumetorum:0.2125,Cerythrinus:0.1055):0.0000):0.0000):0.061
2):0.1039):0.0000):0.0000,(Scurruca:0.0000,((Ptrochilus:0.0000,(Pmodularis:0.
0000,(Pcolchicus:0.0000,((Atrivialis:0.0738,Fhypoleuca:0.0153):0.0080,(Lcolluri
o:0.0000,(Ccinclus:0.0270,(Ccaeruleus:0.0176,(Apratensis:0.0405,Aschoenobaen
us:0.0526):0.0000):0.0000):0.0504):0.0000):0.0415):0.0000):0.0000):0.0000,(R
regulus:0.0000,(Ppyrrhula:0.0000,(Papricaria:0.0000,(Narquata:0.0000,(Lnaevia
:0.0000,(Cfamiliaris:0.0482,(Ccrex:0.0820,(Aalces:0.2146,(Lluscinia:0.0629,(Apa
lustris:0.0919,Hicterina:0.0143):0.0312):0.0001):0.0000):0.0016):0.0395):0.014
5):0.0178):0.0327):0.0036):0.0000):0.0000):0.0000):0.0000):0.0030):0.0346):0.
0362):0.0031):0.0177,(Ttroglodytes:0.0000,(Tviscivorus:0.0000,(Tphilomelos:0.
0000,(Tmerula:0.0000,((Sserinus:0.0000,(Sscrofa:0.0000,((Rnorvegicus:0.0000,(
Eeuropaeus:0.0843,Mnivalis:0.0118):0.0120):0.0000,((Nnatrix:0.0000,(Celaphus
:0.0921,Mputorius:0.0000):0.0197):0.0000,(Mmeles:0.0000,(Mflava:0.0000,(Eru
becula:0.0097,(Acampestris:0.0858,Agraeca:0.1786):0.0311):0.0552):0.0035):0.
0081):0.0205):0.0157):0.0000):0.0000,((Lmuralis:0.1309,Pgraculus:0.0707):0.0
000,(Svulgaris:0.0000,(((Rpendulinus:0.0220,(Cminuta:0.1311,Llusciniodes:0.0
720):0.0160):0.0173,((Teuropaea:0.0000,(Mmartes:0.0253,(Merminea:0.0000,(
Clupus:0.0700,Magrestis:0.0000):0.0020):0.0279):0.0431):0.0000,((Pperdix:0.0
000,(Ppalustris:0.0000,(Aflavicollis:0.1546,(Aterrestris:0.1653,Ozibethicus:0.00
46):0.0000):0.0189):0.0200):0.0309,(Saraneus:0.0000,(Mglareolus:0.0363,(Ccap
reolus:0.1090,Leuropaeus:0.0350):0.0000):0.0249):0.0000):0.0152):0.0000):0.0
000,(Seuropaea:0.0000,(Scommunis:0.0000,(Sborin:0.0000,(Satricapilla:0.0000,
(Saluco:0.0000,(((Pmajor:0.0000,(Pater:0.0089,(Caeruginosus:0.1452,Ooriolus:0
.0000):0.0000):0.0012):0.0000,(Pmontanus:0.0000,((Gglandarius:0.0475,(Fcoel
ebs:0.0545,(Cmonedula:0.0804,(Cchloris:0.0987,(Aotus:0.1227,(Ahypoleucos:0.
1452,(Achrysaetos:0.1991,Rriparia:0.0000):0.0000):0.0000):0.0000):0.0000):0.
0000):0.0000):0.0000,(Ooenanthe:0.0000,(Ftinnunculus:0.0000,(Fperegrinus:0.
0000,(Clivia:0.0000,(Ccoturnix:0.0124,(Aarundinaceus:0.0817,Ascirpaceus:0.01
35):0.0161):0.0061):0.0203):0.0163):0.0400):0.0000):0.0198):0.0000):0.0099,(
(Pochruros:0.0000,((Bbuteo:0.1120,Malba:0.0239):0.0046,(Ccarduelis:0.1059,P
domesticus:0.0000):0.0000):0.0000):0.0000,(Pviridis:0.0000,(Mcinerea:0.0000,(
Ddama:0.0771,Llutra:0.0191):0.0199):0.0295):0.0000):0.0000):0.0147):0.0029)
:0.0000):0.0000):0.0000):0.0033):0.0000):0.0000):0.0000):0.0000):0.0000):0.0
000):0.0000):0.0000):0.0000);}

Supplementary Table 6: Relatedness of the species of ticks and pathogens (exclusive of domesticated vertebrates) to vertebrate dendrograms calculated according to molecular distances or to environmental distances. The number of species of vertebrates in each cluster available for calculation is indicated. Positive values in the column mpd.obs.z and high quantiles (mpd.obs.p > 0.95) indicate phylogenetic evenness. Negative values in the column mpd.obs.z and low quantiles (mpd.obs.p < 0.05) indicate clustering to molecular or environmental features shared with the hosts. NA, not available (fewer than two species available).

	Molecular distances			Environmental distances		
	ntaxa	mpd.obs.z	mpd.obs.p	ntaxa	mpd.obs.z	mpd.obs.p
Apersicus	1	NA	NA	1	NA	NA
Areflexus	1	NA	NA	1	NA	NA
Avespertilionis	3	-0.86	0.14	0	NA	NA
Dmarginatus	20	-2.14	0.01	21	-1.57	0.08
Dreticulatus	10	-1.98	0.01	9	-0.92	0.21
Haegyptium	9	-0.50	0.29	8	-0.65	0.25
Hanatolicum	4	1.23	0.97	3	2.29	1.00
Hconcinna	14	4.02	1.00	13	-0.74	0.25
Hdromedarii	5	0.30	0.81	6	1.38	0.93
Herinacei	3	0.26	0.61	3	0.39	0.62
Hexcavatum	5	-0.95	0.13	5	-0.20	0.41
Himpeltatum	6	0.41	0.73	6	2.18	1.00
Hlusitanicum	5	-0.40	0.28	4	0.95	0.85
Hmarginatum	55	-4.36	0.01	50	-2.60	0.01
Hparva	1	NA	NA	0	NA	NA
Hpunctata	39	-1.07	0.10	37	-3.26	0.01
Hrufipes	12	-2.66	0.02	12	-0.29	0.36
Hsulcata	25	0.32	0.76	24	-0.04	0.48
Iacuminatus	5	-1.71	0.01	6	-2.38	0.03
Iapronophorus	3	-1.48	0.05	3	-0.52	0.29
Iarboricola	14	-2.10	0.01	16	-5.07	0.01
Icanisuga	2	-1.30	0.10	2	-1.32	0.10
Icrenulatus	2	-0.80	0.29	3	-0.95	0.19
Ieldaricus	1	NA	NA	2	-0.90	0.23
Ifestai	1	NA	NA	1	NA	NA
Ifrontalis	19	-4.56	0.01	19	-4.83	0.01
Igibbosus	3	-0.34	0.29	3	-1.47	0.07
Ihexagonus	12	-3.16	0.01	14	-2.02	0.05
Ilaguri	2	-0.67	0.24	2	-1.21	0.13
Ilividus	0	NA	NA	1	NA	NA
Ipersulcatus	2	1.00	0.92	1	NA	NA
Iredikorzevi	15	-0.96	0.12	14	-2.33	0.03
Iricinus	124	-1.13	0.16	134	-7.65	0.01
Irugicollis	1	NA	NA	1	NA	NA
Isimplex	2	-0.59	0.33	0	NA	NA
Itrianguliceps	14	-2.60	0.01	15	-1.91	0.04
Iuriae	3	-0.77	0.11	3	-1.12	0.17
Iventalloi	14	-1.03	0.11	17	0.10	0.58
Ivespertilionis	10	-1.65	0.02	0	NA	NA
Oerraticus	3	-1.13	0.10	4	0.76	0.75
Otholozani	1	NA	NA	1	NA	NA
Rannulatus	9	3.02	0.99	10	-0.09	0.45
Rbursa	11	-0.68	0.16	11	0.18	0.53
Rpusillus	3	-1.08	0.12	3	-0.99	0.18
Rrossicus	0	NA	NA	1	NA	NA
Rsanguineus group	41	-3.29	0.01	45	2.76	1.00
Amarginale	1	NA	NA	1	NA	NA
Aovis	1	NA	NA	1	NA	NA
Aphagocytophilum	14	-6.19	0.01	14	-0.03	0.49

Bafzelii	11	-3.67	0.01	11	-0.81	0.24
Bbigemina	2	-1.42	0.14	2	-0.49	0.38
Bbovis	2	-1.50	0.08	2	-0.40	0.39
Bburgdorferi s.l.	28	-3.41	0.02	27	-1.77	0.07
Bburgdorferi s.s.	8	-4.02	0.02	8	-0.75	0.25
Bcapreoli	3	-2.73	0.03	3	0.11	0.67
Bdivergens	2	-1.45	0.08	2	-0.50	0.36
BEU1	2	-1.56	0.06	2	-0.29	0.44
Bgarinii	9	-0.95	0.13	9	-1.12	0.16
Bhenselae	1	NA	NA	1	NA	NA
Blusitaniae	4	-2.39	0.06	2	0.44	0.70
Bmicroti	7	-4.11	0.01	7	-0.20	0.47
Bturdi	1	NA	NA	1	NA	NA
Bvalaisiana	1	NA	NA	1	NA	NA
Bvinsonii	2	-0.87	0.24	2	0.30	0.69
Cburnetti	4	-2.30	0.04	4	1.11	0.85
CCHFv	1	NA	NA	1	NA	NA
Ftularensis	7	-4.25	0.01	7	-0.34	0.46
Hcanis	1	NA	NA	1	NA	NA
Nmikurensis	4	-2.46	0.05	4	0.06	0.56
Rhelvetica	4	-0.99	0.16	2	-0.33	0.50
Rmonacensis	1	NA	NA	0	NA	NA
Rslovaca	2	-2.07	0.01	2	0.67	0.75
Tannulata	1	NA	NA	1	NA	NA
TBEv	7	-4.98	0.01	7	-0.61	0.25
